# Endometriosis-related alterations in the endometrium revealed by integrated single-cell and AI-powered approaches

**DOI:** 10.1038/s41467-026-73020-4

**Published:** 2026-05-20

**Authors:** Lea Duempelmann, Shaoline Sheppard, Brett McKinnon, Angelo Duo, Jitka Skrabalova, Thomas Andrieu, Ryan Lusby, Wiebke Solass, Dennis Goehlsdorf, Sukalp Muzumdar, Cinzia Donato, Hans Bösmüller, Sarah Carl, Peter Nestorov, Michael D. Mueller

**Affiliations:** 1 Endometriosis & Gynaecological Oncology Laboratory, Department for BioMedical Research, University of Bern, Bern, Switzerland; 2 Department of Obstetrics and Gynecology, Inselspital, Bern University Hospital, University of Bern, Bern, Switzerland; 3 Scailyte AG, Basel, Switzerland; 4 Institute for Molecular Bioscience, The University of Queensland, Brisbane, QLD Australia; 5 Institute of Tissue Medicine and Pathology, University of Bern, Bern, Switzerland; 6 Hera Biotech, Inc., 1475 college park, San Antonio, TX USA; 7 Department of Pathology, University Hospital Tuebingen, Tuebingen, Germany

**Keywords:** Transcriptomics, Molecular medicine, Diagnostic markers, Computational models, Cellular signalling networks

## Abstract

Endometriosis, affecting 1 in 9 women, presents treatment and diagnostic challenges. To address these issues, we generated a comprehensive single-cell atlas of endometrial tissue, comprising 466,371 cells from 35 endometriosis and 25 non-endometriosis donors without exogenous hormonal treatment. Detailed analysis reveals significant gene expression changes and altered receptor-ligand interactions present in the endometrium of endometriosis patients, including increased inflammation, adhesion, proliferation, cell survival, and angiogenesis in various cell types. These alterations may enhance endometriosis lesion formation and identify potential therapeutic targets. Using ScaiVision, we trained neural network models to predict endometriosis of varying disease severity (median AUC = 0.83), including one model based solely on a set of 11 genes confirmed as dysregulated in endometriosis patients through differential expression analysis. In conclusion, our findings reveal numerous pathway and ligand-receptor changes in the endometrium of endometriosis patients, offering insights into pathophysiology, potential targets for improved treatments, and predictive models for enhanced outcomes in endometriosis management. Our models, while not yet externally validated, can serve as a tool for hypothesis generation and starting point for further clinical development.

## Abstract (web summary)

Endometriosis affects 1 in 9 women, yet its biology and diagnosis remain poorly understood. Here, the authors show that a single-cell atlas of the eutopic endometrium uncovers inflammatory and signaling changes linked to endometriosis and supports gene-based models that predict disease.

## Introduction

Endometriosis is a prevalent gynecological disease affecting approximately 11% of women of reproductive age worldwide^1^ and imposing substantial societal and economic burdens due to its chronic nature and debilitating symptoms^2^. Characterized by the abnormal growth of endometrial tissue outside the uterus, endometriosis manifests through chronic pelvic pain, intense pain during periods and intercourse, and infertility^3,4^. The diagnosis of endometriosis is challenging due to the variability in clinical symptoms and the absence of confirmed in vitro diagnostics (IVDs)^5^. This results in a 6 to 11-year delay on average between the onset of symptoms and diagnosis with surgical laparoscopy^6^. The delay not only prolongs patients’ suffering from inadequate management strategies but also lets the untreated disease advance to more severe stages^7^.

Current limited treatment options and diagnostic challenges underscore the need for a comprehensive understanding of endometriosis pathophysiology and for accurate in vitro diagnostic tools. Sampson’s widely accepted 1927 theory postulated that shed endometrial tissue during menstruation is transported to the peritoneal cavity, where it forms endometriotic lesions^8^. Since most women experience retrograde menstruation, other risk factors have been suggested to contribute to the development of endometriosis, such as differences in the endometrium^9,10^.

Monthly hormonal influences drive extensive endometrial remodeling, encompassing proliferation, lineage specialization, and vascularization, resulting in marked functional and morphological changes^11,12^ that render the endometrium an exceptionally complex tissue to study. The largest endometrial bulk-sequencing studies with over 200 donors and stratified by menstrual cycle phases reported no significant gene expression changes between women with and without endometriosis^13,14^. Nonetheless, bulk sequencing may mask influential gene expression changes of rare yet critical cell types in such highly complex tissue. The advent of single-cell sequencing has revolutionized our ability to dissect complex tissues at an unprecedented resolution^15^. However, recent single-cell endometriosis studies including the eutopic endometrium faced limitations, such as small eutopic sample size, cell type exclusion, exogenous hormone treatment, or did not consider menstrual cycle phase changes^16–21^. A single-cell study of entire endometrium biopsies from non-hormonally treated patients throughout the menstrual cycle is currently lacking.

In this study, we present a comprehensive single-cell atlas of endometrial tissue, comprising 466,371 cells from 35 endometriosis and 25 control donors, all without exogenous hormones, across different menstrual cycle phases. Through detailed cell annotation, we identify significant gene expression alterations in various endometrial cell types among donors with endometriosis, particularly in pathways related to inflammation, adhesion, proliferation, cell survival, and angiogenesis. Furthermore, several receptor-ligand interactions across various cell types were altered, suggesting a pivotal role for macrophages and, in turn, stimulating inflammation, angiogenesis, proliferation, and cell survival, which may favor endometriosis susceptibility. Leveraging convolutional neural networks via ScaiVision, we have developed models that effectively predict mild and severe endometriosis in our cohort, with high AUCs (median AUC = 0.83 across 3 cross-validation splits) based on either all endometrial cells or exclusively cycling mesenchymal cells. Additionally, we have successfully distilled a concise signature comprising only 11 genes, which, when used as input features to retrain ScaiVision models, remarkably retains the same level of predictive performance on held-out samples (median AUC = 0.83). This 11-gene signature shows promise as both a foundation for further predictive modeling efforts, which should be validated in additional external cohorts, and as a basis for deeper biological investigation. In conclusion, the findings from our single-cell atlas reveal several altered pathways and ligand-receptor interactions in different cell types, identifying potential targets for more effective non-hormonal treatments and have led to the generation of predictive models that are currently being further developed into in vitro diagnostic assays for endometriosis.

## Results

### Generation of the menstrual cycle stage annotated single-cell endometrial atlas for the investigation of endometriosis

**Fig. 1:**
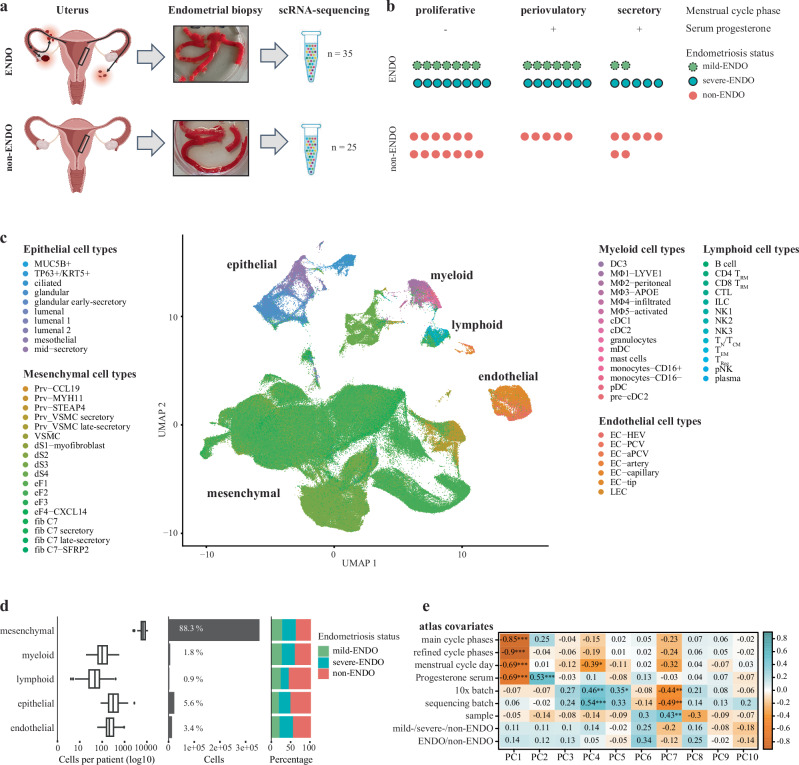
Single-cell atlas of the endometrium from women with and without endometriosis. **a** Schematic illustration of the uterus (left), image of endometrial biopsies (middle), followed by single-cell RNA (scRNA)-capture with 10x Genomics and scRNA-sequencing with Illumina (right). The endometrial single-cell atlas includes 35 endometrial biopsies from women with endometriosis (ENDO) and 25 endometrial biopsies from women without endometriosis (non-ENDO). The black box in the uterus schematic delineates parts of the endometrium. The most widely accepted theory of endometriosis origin (Sampson, 1927) postulates the migration of shed endometrial tissue during menstruation to the peritoneal cavity (uterus schematic, black errors), where it forms endometriotic lesions (uterus schematic, red cell aggregates). *Created in BioRender. Duempelmann, L. (2026)* https://BioRender.com/uo59ho0. **b** Distribution of samples in regard to menstrual cycle phase (proliferative, periovulatory and secretory) and endometriosis status (mild-ENDO, severe-ENDO and non-ENDO). Women with proliferative menstrual cycle phase did not have measurable serum progesterone ( < 2nmol/l). **c** UMAP of the entire endometrium single-cell atlas from women with and without endometriosis, colored by cell types (*n* = 63). **d** Major cell type frequencies per donor (*n* = 60, left) shown as box plots (Center line, median; box limits, 25th–75th percentiles (IQR); whiskers, most extreme points within 1.5×IQR; points, outliers); overall cell type frequencies (middle) as bar plots, with mesenchymal cells being the most abundant cell type; total cell numbers across non-ENDO, mild-ENDO, and severe-ENDO groups (right) are balanced. **e** Eigencor plot displaying Pearson correlation coefficients between atlas dataset covariates and principal components (PCs), with significance calculated using two-tailed Student’s tests and adjusted for multiple testing (Benjamini–Hochberg). Menstrual cycle phase parameters significantly correlate with PC1, PC2 and PC4. In contrast, no significant correlation was identified between the endometriosis status and any of the initial ten principal components. Statistical significance symbols represent: *** <0.001, ** <0.01, * <0.05.

To confidently characterize cells within the endometrium, identify transcriptomic changes across the menstrual cycle, and reveal endometriosis-specific differences, we performed single-cell sequencing (10x Genomics) on endometrial biopsies collected from 60 women prior to laparoscopic surgery. Of these women, 35 were confirmed to have endometriosis through histopathological evaluation (ENDO), and 25 donors were endometriosis-free (non-ENDO) (Fig. 1a). The menstrual cycle phase of each donor was assessed from menstrual cycle day, serum progesterone levels, histological endometrium evaluation (if available) by two independent pathologists, and the transcriptional profile. Samples from different menstrual cycle phases were included, as were mild (rASRM stages I-II, mild-ENDO) and severe (rASRM stages III-IV, severe-ENDO) endometriosis cases (Fig. 1b). Any donors taking exogenous hormones within 3 months before surgery were excluded, due to the heterogeneous impact of hormones on the transcriptional landscape of the endometrium^22^ (Supplementary Fig. 3g, h). Additional exclusion criteria encompassed other inflammatory diseases, malignancy, pregnancy, and lactation.

We successfully created a comprehensive single-cell atlas of the endometrium, comprising 283,789 ENDO and 182,582 non-ENDO donor cells. The samples had, on average, 7773 cells, with 4,604 counts and 2,419 features per cell after SCT normalization, displaying no discernible difference between endometriosis status in terms of quality or gross characteristics (Supplementary Fig. 1a, b). Using Symphony^23^, we identified 57 cell types by alignment to a reference atlas specifically for endometrium and endometriosis^18^. The characteristic cell type marker genes were expressed as in the reference atlas^18^ (Supplementary Fig. 2). The cell type annotation was further refined to 63 cell types by graph-based clustering to accommodate changes during the menstrual cycle (Fig. 1c, Supplementary Fig. 1c, d, Methods). The cell types can be categorized into five major cell types: mesenchymal (88.3%), epithelial (5.6%), endothelial (3.4%), lymphoid (0.9%) and myeloid (1.8%) (Fig. 1d). The transcriptional variance within the dataset was captured through sample-wise Principal Component Analysis (PCA), revealing a significant correlation between the first Principal Components (PC1, PC2 and PC4) and key menstrual cycle phase parameters, including menstrual cycle phase, cycle day and serum progesterone levels (Fig. 1e). Surprisingly, no statistically significant correlation was identified between the endometriosis status and any of the initial ten Principal Components (Fig. 1e). This observation also held true when PCA was performed on each major cell type separately, except for myeloid cells, for which endometriosis status and PC3 significantly correlated (Supplementary Fig. 1e, f and 3a). This suggests a minor impact of the endometriosis status on the endometrial transcriptional landscape, consistent with previous bulk-sequencing studies^13,14^. The nuanced influence of endometriosis on the molecular characteristics of endometrial tissue underscores the necessity of accounting for menstrual cycle phase, minimizing confounding factors, and exploring subsets of cells for a more comprehensive understanding of the relationship between endometriosis and the endometrial transcriptome. To mitigate confounding factors, we generated our single-cell atlas within the same laboratory, employing unvarying processing methods and encompassing detailed clinical data with menstrual cycle staging (Supplementary data 1). This approach provides a powerful, unmatched resource for investigating endometrial biology and diagnostic signatures for endometrial pathologies like endometriosis.

### Cellular changes during the menstrual cycle drive transcriptional changes in the endometrium

**Fig. 2:**
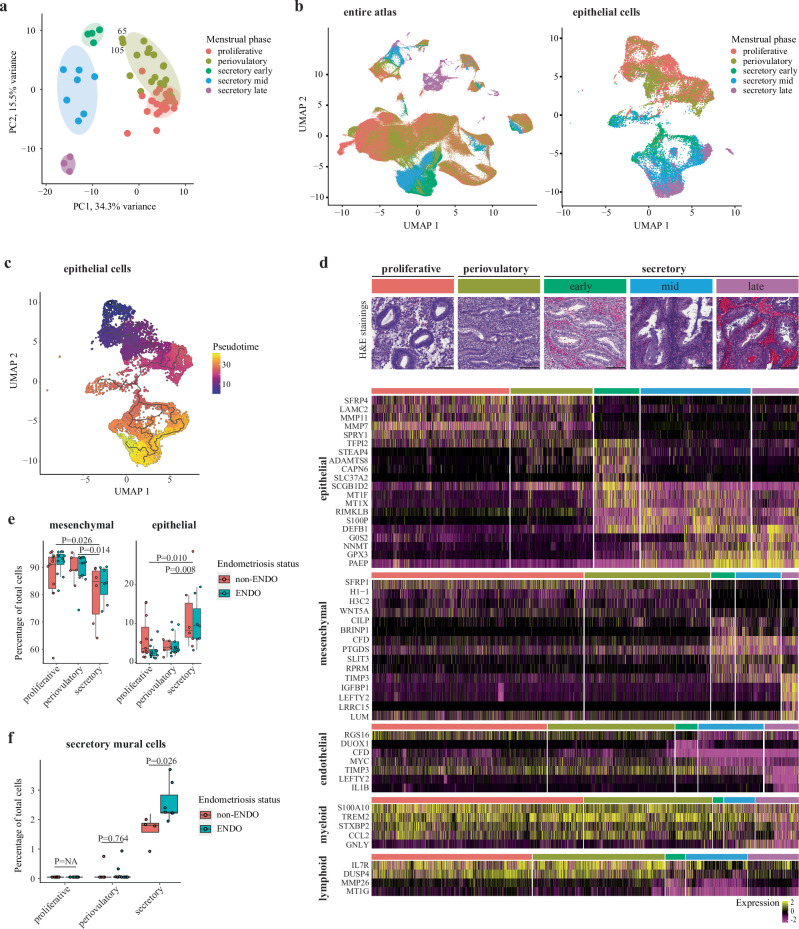
Cellular changes during Menstrual Cycle drive transcriptional changes in the endometrium. **a** Scores plot of the first two principal components from PCA of sample-wise whole transcriptome. PC1 accounts for 34.3% of the total variance, while PC2 explains 15.5% (PCAtools). The proliferative and periovulatory samples do not cluster distinctly, whereas early-, mid- and late-secretory samples cluster separately, marked by ellipses (t’ distribution, 0.90 confidence interval, late-secretory ellipse added manually). Samples 65 and 105 (labeled) are in the transition between periovulatory and early secretory phases. **b** UMAPs of the entire dataset (left) and the re-integrated epithelial cells (right), colored by menstrual cycle phase, show a clear visual separation between proliferative/periovulatory, early-, mid-, and late-secretory samples, particularly in mesenchymal (endometrial fibroblasts, C7 fibroblasts, and mural cells), epithelial, and endothelial cells. **c** UMAP of the re-integrated epithelial cells, colored by pseudo time prediction from monocle3. **d** Top: Representative H&E staining from each key menstrual cycle phase (proliferative, periovulatory, early-, mid-, and late-secretory; scale bar: 0.1 mm). Bottom: Heatmap showing expression of selected marker genes with high log fold changes across the key menstrual cycle phases in the major cell types. Samples were ordered by menstrual cycle phase and pseudo time. **e** Box plots show sample-wise mesenchymal and epithelial cell frequencies (% of total cells) by menstrual cycle phase. Center line, median; box limits, 25th–75th percentiles (IQR); whiskers, most extreme points within 1.5×IQR; points, individual samples. Stringent donor exclusion criteria were applied (32 ENDO, and 22 non-ENDO samples). P-values were calculated using a two-tailed Student’s t-test, adjusted for multiple testing (Benjamini-Hochberg), and annotated on the plot. **f** Box plots show sample-wise secretory mural cell frequencies (% of total cells) by menstrual cycle phase. Center line, median; box limits, 25th–75th percentiles (IQR); whiskers, most extreme points within 1.5×IQR; points, individual samples. Stringent donor exclusion criteria were applied, and late secretory samples were excluded due to the imbalance in endometriosis status (32 ENDO, and 20 non-ENDO). P-values were calculated using a two-tailed Student’s t-test, adjusted for multiple testing (Benjamini-Hochberg) and annotated on the plot.

Significant cellular content, morphology, and transcriptional changes accompany the different menstrual cycle stages^11,24^. In the PCA plot of PC1/PC2 (Fig. 2a), proliferative/periovulatory and early-, mid-, and late-secretory samples cluster distinctly, underscoring the substantial impact of the menstrual cycle on the endometrial transcriptional landscape^24^, with PC1 and PC2 together accounting for 49.9% of the total variance. Moreover, a clear visual separation in the UMAP between proliferative/periovulatory, early-, mid-, and late-secretory samples is evident, particularly in mesenchymal, epithelial, and endothelial cells (Fig. 2b). Interestingly, proliferative and periovulatory samples overlapped by PCA and UMAP (Fig. 2a, b), suggesting a similar transcriptional profile and a delayed effect of progesterone on endometrial transcription. Epithelial cells from early-, mid-, and late-secretory phases exhibited a clear gradient on the UMAP, prompting a pseudotime trajectory analysis with Monocle to infer a more detailed order of the samples within the menstrual cycle phases (Fig. 2c, Supplementary Fig. 3b).

To characterize the transcriptomic alterations across the major cell types throughout the menstrual cycle, a comparative analysis with Seurat^25^ was conducted of the transcriptome across the key menstrual cycle phases. This investigation, stratified by the primary five cell types, uncovered 3643 genes significantly changing in expression over the menstrual cycle phases (adjusted p-value < 0.05, avg_log2FC > 2) (Fig. 2d, Supplementary data 2), underscoring the substantial transcriptomic variations during the menstrual cycle (Fig. 1e). The observed expression changes of menstrual marker genes mirrored the sequence outlined in a comprehensive C1 menstrual cycle atlas from healthy volunteers with a regular cycle^24^. Notably, retrospective alignment of our atlas samples to their menstrual cycle phases^24^, using Symphony^23^, resulted in perfect correspondence (Supplementary Fig. 3c–f). This alignment instills a high level of confidence in the accuracy of our menstrual cycle staging. Our single-cell atlas, with almost 50 times more cells per donor and detailed annotation, establishes a comprehensive reference for future datasets and provides detailed insights into the intricacies of menstrual cycle dynamics.

An investigation into changes in cell type frequencies across menstrual cycle phases reveals significant shifts. Generally, epithelial cells increase, while mesenchymal cells decrease from proliferative to secretory phase (Fig. 2e, Supplementary Fig. 4a). Approximately a third of the refined cell types show significant abundance changes during the menstrual cycle (Supplementary data 3). Examining cell type frequency differences between ENDO and non-ENDO, stratified by the main menstrual cycle phases, the only notable abundance change was observed in secretory mural cells (Prv_VSMC secretory) (Supplementary Fig. 4b). Mural cells are important for vascular development and stability. The significant 1.6-fold increase of secretory mural cells in ENDO in comparison to non-ENDO (adjusted p-value = 0.026, Fig. 2f) suggests increased vascularization in the secretory endometrium of endometriosis patients. This is consistent with the detected increase of perivascular endometrial mesenchymal stem cells in menstrual fluid in endometriosis^26^, supporting the ‘stem cell hypothesis’ of endometriosis pathogenesis^27^.

### Transcriptional alterations in the endometrium of women with endometriosis

Next, we investigated whether transcriptional changes can be observed in the endometrium of women with endometriosis and if these changes can be associated with processes suggested to contribute to endometriosis susceptibility^9^. To detect robust alterations in transcription between endometriosis status, we focused on the relatively homogeneous proliferative menstrual cycle phase (Fig. 2)^11,24^.

**Fig. 3:**
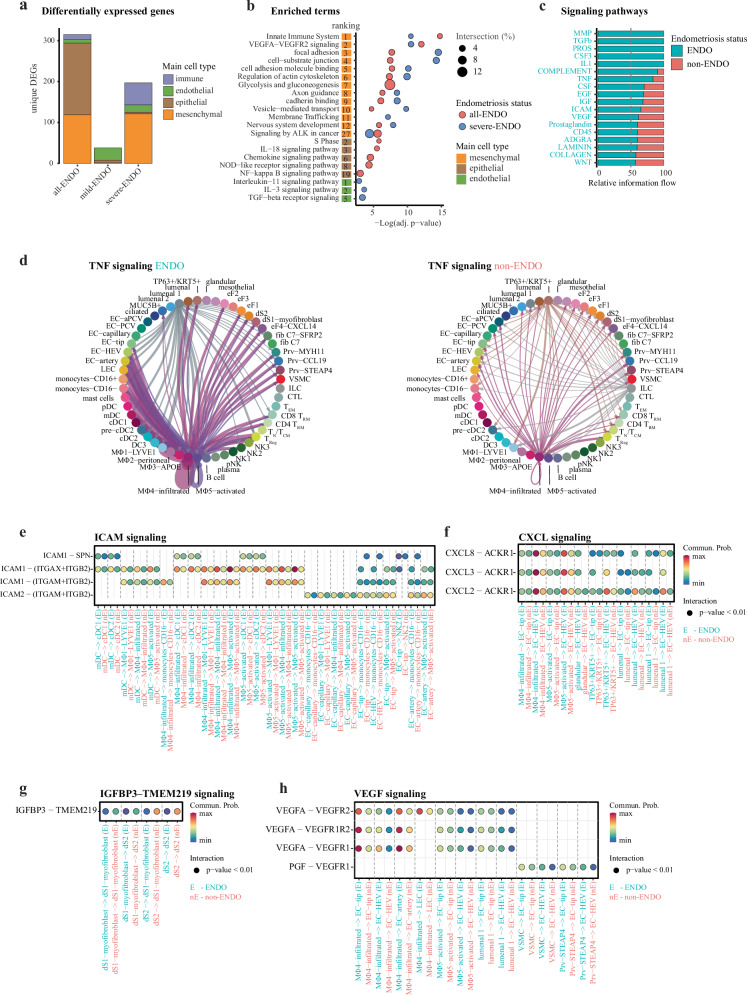
Inflammation, Adhesion, Proliferation, and Angiogenesis pathways and ligand-receptor interactions are upregulated in endometriosis endometrium of the proliferative phase. **a** Bar plot showing number of unique differentially expressed genes (DEGs, adjusted p-value < 0.05, average expression > 18.53202) between non-ENDO (*n* = 11) and all-ENDO (*n* = 12), mild-ENDO (*n* = 4) and severe-ENDO (*n* = 8) samples within immune, endothelial, epithelial, and mesenchymal cell types, analyzed by Muscat. DEG analysis identified overall 427 unique DEGs between non-ENDO and mild-, severe- or all-ENDO (38, 192, and 309 DEGs, respectively). Compared to non-ENDO, there were 5 times more DEGs in severe-ENDO than in mild-ENDO. The lymphoid and myeloid immune cells of severe-ENDO displayed 54 DEGs, in stark contrast to the absence of any immune DEGs in mild-ENDO. **b** Selected terms enriched in the cell-type- and stage-wise functional enrichment analysis of DEGs, with term sizes between 25 and 750. The rank by adjusted p-value is indicated under ranking. Statistical significance was determined using a one-sided cumulative hypergeometric test, with p-values adjusted for multiple testing (g:SCS method, R gprofiler2). **c** Selected signaling pathways with overall enriched activity in ENDO (*n* = 12) in comparison to non-ENDO (*n* = 11). **d** Circle plot of TNF signaling pathway between all cell types in ENDO (left, *n* = 12) and non-ENDO (right, *n* = 11). **e–h** Bubble plots of selected communications with significant interaction probabilities (unadjusted p-value < 0.01) within the ICAM (**e**), CXCL (**f**), IGFBP (**g**), and VEGF (**h**) signaling pathways for ENDO (*n* = 12) and non-ENDO (*n* = 11). Statistical significance was determined using a one-sided permutation test. Further p-value adjustment is not recommended by the CellChat author.

Cell-type-wise differential gene expression analysis revealed 427 unique differentially expressed genes (DEGs, adjusted p-value < 0.05) between non-ENDO and mild-, severe- or all-ENDO using Muscat^28^ (Fig. 3a, Supplementary data 4). Notably, severe-ENDO exhibited a fivefold increase in DEGs compared to mild-ENDO (Fig. 3a). Cell-type- and stage-wise functional enrichment analysis identified significant perturbations in inflammation, cellular adhesion, cell proliferation and survival, angiogenesis, and the nervous system, as detailed in the following sections (Fig. 3b, Supplementary data 5).

Using CellChat^29^, we quantitatively analyzed intercellular communication networks, revealing overall enriched activity of several ligand-receptor interactions in the endometrium of women with ENDO, including TGFb, IL1, TNF, ICAM, VEGF, and WNT (Fig. 3c, Supplementary Fig. 5b). These interactions suggest stimulated inflammation, angiogenesis, proliferation, and cell survival as discussed in more detail below.

These findings show that regulatory disturbances are present in the endometrium of women with endometriosis and are more pronounced in severe endometriosis cases. Functional enrichment and ligand-receptor analysis highlight dysregulation of inflammation, cell proliferation, survival, adhesion, angiogenesis, axonal guidance and WNT/NOTCH pathways, consistent with the processes thought to be required for endometrial cells to establish viable lesions at ectopic sites. Enhanced interactions in ENDO between macrophages and other cell types point to a pivotal role for macrophages (Fig. 3, Supplementary Fig. 5). These findings advance understanding of the molecular intricacies of endometriosis, which occur early in disease development and offer potential targets for therapeutic intervention.

### Inflammation and adhesion are enhanced in ENDO endometrium

Macrophages play a central role in normal endometrial tissue remodeling and endometriosis lesions, contributing to growth, development, vascularization, and lesion innervation^30^. Endometrial macrophages were proposed to favor endometriosis lesion formation; however, the mechanism is convoluted^31^. We observed a significantly enhanced interaction between macrophages and stromal, endothelial, epithelial and immune cells through TNF – TNFR1/2 signaling (Fig. 3d, Supplementary Fig. 5d). Binding of tumor necrosis factor (TNF) to TNF receptor 1 (TNFR1) activates NFκB and mitogen-activated protein kinase (MAPK) signaling pathways, responsible for regulating transcription of pro-inflammatory mediators including cytokines, chemokines and the atypical chemokine receptor ACKR1^32–34^. TNF is also dysregulated in endometriosis lesions^35^. Furthermore, there is a strongly enhanced interaction in ENDO between macrophages, dendritic cells, and endothelial cells with multiple immune cells mediated through ICAM1/2 and its receptors (Fig. 3e). ICAM expression is strongly triggered in epithelial and immune cells under inflammatory stimulation, and TNF-inducible ICAM1 regulates the recruitment of circulating leukocytes to sites of inflammation^36,37^. Moreover, we observed an enhanced interaction between macrophages and epithelial cells with endothelial cells through the chemokines CXCL2, CXCL3, and CXCL8 with ACKR1 (Fig. 3f), known to regulate inflammatory response.

The complex interplay among different cell types via secreted cytokines is critical in the inflammatory microenvironment^38^. Our analyses further revealed increased cytokine expression (*CXCL2*, *CXCL3*, *TGFB1*) and upregulated IL-3, IL-11 and IL-18 signaling pathways in ENDO (Fig. 3b, Supplementary data 5). Genes in the NFκB pathway and *JAK1*, previously implicated in endometriosis lesions^35,39^, were both significantly upregulated at the RNA level in ENDO endometrium (Fig. 3b, Supplementary data 4). NFκB orchestrates inflammatory responses by promoting the synthesis of pro-inflammatory cytokines and chemokines, such as IL1, IL6, IL8, TNFa, RANTES, MIF, and ICAM1, while JAK1 regulates the immune system and proinflammatory response^32,40^. Moreover, the deregulation of *CXCL12* (*SDF-1*) in mesenchymal cells, which in bone marrow supports the maintenance of hematopoietic stem cell niche^41^ and may play a role in the stem/progenitor nature of endometrial mesenchymal cells^42^, could increase immature endometrial cells and thereby endometriosis susceptibility.

Upon entry into the peritoneal cavity, adhesion of refluxed endometrial cells facilitates attachment to the underlying peritoneal tissue and contributes to migration and invasion^9^. Several genes involved in focal adhesion and cytoskeleton reorganization, including *ACTB*, *RHOA*, *COL4A2*, *COL5A2*, *JAK1*, and *MYH9*, were upregulated in mesenchymal cells (Fig. 3b, Supplementary Fig. 5a), in line with stromal cells leading invasion^43^. Further upregulated in ENDO mesenchymal cells was *TGFB1* | *1* (Supplementary data 4), shown to promote focal adhesion, migration, and EMT in carcinoma^44^. Integrin beta-1 (*ITGB1*), upregulated in mesenchymal cells of endometriosis patients, has been reported to enhance invasiveness and adhesion of endometrial fibroblasts, possibly contributing to the disease’s pathophysiology^45^.

### Proliferation and cell survival are enhanced in ENDO endometrium

We observed increased expression of genes associated with cell proliferation and survival pathways, including NFκB signaling and ALK signaling in ENDO epithelial and mesenchymal cells (Fig. 3b) as well as decreased cell death receptor signaling (Fig. 3g)^32,46,47^. NFκB, known for its role in cell proliferation, apoptosis suppression, and adhesion molecule expression, is overactive in endometriotic lesions^35^. Its activation by NOD-like receptors, also significantly enriched (Fig. 3b), underscores its importance^32^. NFκB inhibitors show potential as non-hormonal drugs against endometriosis, reducing inflammation, angiogenic factors, and MMPs in vitro and lesion size in animal models^35,48^. The ALK signaling pathway, among the top 30 enriched terms in mesenchymal cells (Fig. 3b), mediates signaling through various pathways, leading to increased cell proliferation, cell survival, and cell cycle progression^49^. Ligand-receptor analysis further indicates reduced apoptosis in ENDO by decreased signaling between the cell death receptor TMEM219 and IGFBP3^50^ in several mesenchymal cell-cell interactions in ENDO (Fig. 3g). Hematopoietic cell kinase (*HCK*), upregulated in myeloid cells (Supplementary data 4), has been shown to inhibit proliferation and promote apoptosis of ectopic endometrial cells in vivo and in vitro^51^. This is particularly interesting, as HCK was identified as a potential therapeutic target for endometriosis^51^. Enhanced proliferation, enhanced cell survival and decreased apoptosis may contribute to effective endometriosis lesion formation^32,46,47^.

### Angiogenesis is enhanced in ENDO endometrium

Angiogenesis is essential for sustaining endometriosis lesions by ensuring nutrient supply^9^. VEGFA-VEGFR2 signaling (Fig. 3b) and several angiogenesis-mediating ligand-receptor interactions (Fig. 3h) were enhanced in the ENDO. The interaction between macrophages and endothelial cell types is increased through VEGFA-VEGFR1/-VEGFR1R2/-VEGFR2 in ENDO. VEGFA-VEGFR2 is the most prominent ligand-receptor complex in the VEGF system^52^, and genetic variants in the *VEGFR2* gene (*KDR*) have previously been associated with endometriosis susceptibility^53^. Furthermore, an enhanced interaction in ENDO between mural cells (perivascular (Prv-STEAP4), vascular cells (VSMC)) and endothelial cells (EC-tip, EC-HEV) through angiogenesis-promoting PGF-VEGFR1 ligand-receptor binding^54^ was observed (Fig. 3h). VEGF signaling through VEGFR1/VEGFR2 ultimately promotes endothelial cell proliferation, migration, survival, and vascular permeability^52^. Compellingly, the abundance of secretory mural cells is significantly higher in ENDO than in non-ENDO (Fig. 2f), which aligns with an increase in endothelial cell proliferation through enhanced VEGF signaling.

Our analysis further unveiled significant enrichment of TGFb signaling in endothelial cells, by upregulation of genes, such as *TGFB1* and *JAK1* (Supplementary Fig. 5a, c). TGFb is an inflammatory growth factor that regulates cell adhesion, invasion, and angiogenesis^55^, and was found to be upregulated in endometriosis lesions^9^. In tumors, TGFb induces the metabolic conversion of glucose to lactate via glycolysis, a process referred to as the “Warburg effect”, with lactate increasing angiogenesis and cell invasion^56^. Glycolysis and gluconeogenesis term were significantly enriched in ENDO mesenchymal cells (top 7; Fig. 3b) suggesting a possible occurrence of a Warburg-like effect, in line with a peritoneal endometriosis report^57^. Dysregulation of TGFb signaling pathway contributes to fibrosis and neovascularization in various diseases, underscoring its therapeutic potential in endometriosis management^58,59^.

In summary, we observed enhanced angiogenesis signaling and ligand-receptor interactions as well as an increase of secretory mural cells in the endometrium of women with endometriosis, which may explain increased endometriosis susceptibility.

### Nervous system and WNT/NOTCH in ENDO endometrium

Painful symptoms associated with endometriosis lesions are proposed to arise from interactions with nerve fibers at ectopic sites^60,61^, mediated by both immune and mesenchymal-derived inflammation^62,63^ which stimulate sensory nerve fiber infiltration into established lesions^64^. However, controversy exists regarding the increase in sensory nerve fibers in the endometrium of women with endometriosis^62^. Axon guidance and nervous system development were among the top 20 enriched terms (Fig. 3b) in ENDO mesenchymal cells. This suggests a possible augmentation of nerve fibers in the endometrium of women with endometriosis.

The endometrium undergoes monthly regeneration driven by mesenchymal stem and epithelial progenitor cells and relies on programmed differentiation and maturation for normal function. We detected a subtle increase in overall WNT signaling (WNT2/3/7 A - (FZD3/4/6 + LRP6)) and a marginal increase in overall NOTCH signaling in ENDO (Fig. 3c, Supplementary Fig. 5b). Notably, crosstalk between NOTCH and WNT pathways affects epithelial cell differentiation, with increased WNT activity leading to enhanced ciliary commitment^12^, a feature observed in endometriosis lesions^47^.

### Prediction of endometriosis based on all cells or cycling fibroblasts using ScaiVision

Next, we trained predictive models for endometriosis based on gene expression changes within the endometrium. We employed the machine learning algorithm CellCnn via Scailyte’s ScaiVision platform, which can assess and integrate expression changes of hundreds of genes across hundreds of thousands of single cells simultaneously and employ representation learning to discover relevant biomarkers for disease modeling^65^.

**Fig. 4:**
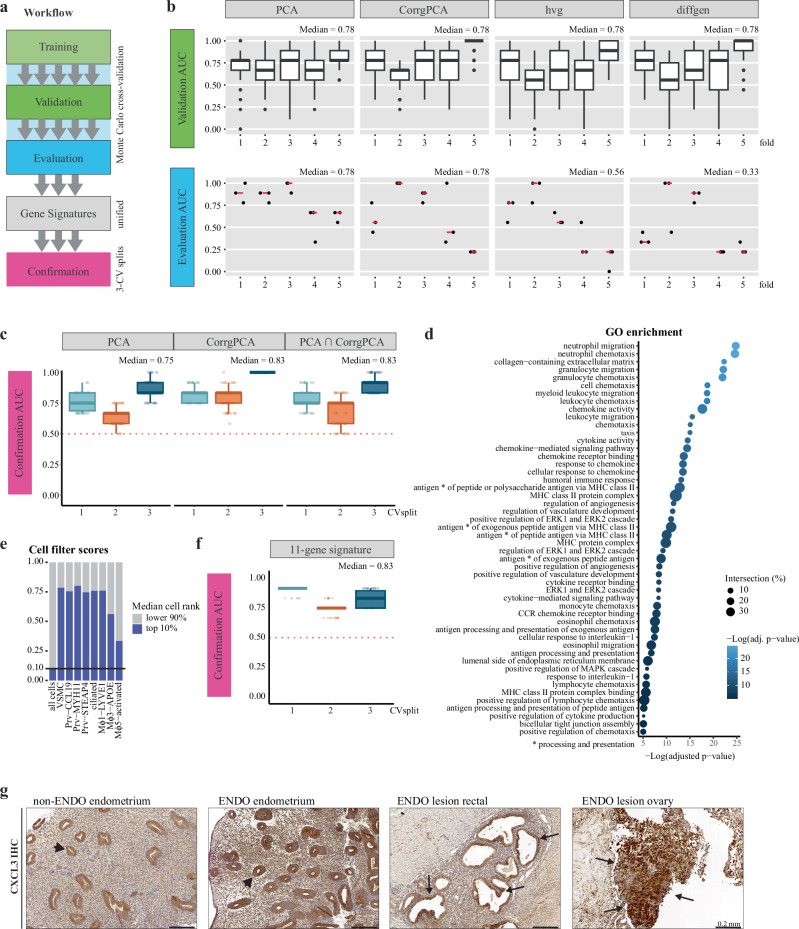
Prediction of endometriosis with ScaiVision using convolutional neural networks. **a** Workflow of ScaiVision model training and gene signature confirmation. Proliferative samples (*n* = 25) were allocated for training/validation (76%) and evaluation (24%) using 5-fold Monte Carlo cross-validation. Training/validation samples were further stratified with 5-CV splits, yielding 25 datasets. Unified gene signatures were derived by selecting the genes with top 20% deepLIFT and integrated gradients importance scores and evaluated across 3-CV splits. **b** Box plots show validation AUCs (*n* = 250 per fold and feature selection method) and evaluation AUCs (*n* = 3 per fold and method; median, red lines), grouped by the feature selection method (PCA, CorrgPCA, hvg, diffgen). PCA and CorrgPCA achieved the highest evaluation performance (median AUC = 0.78). **c** Box plots of confirmation AUCs (*n* = 50 per box plot) for the unified PCA and CorrgPCA gene signatures and their intersect (PCA ∩ CorrgPCA), with median AUCs consistently >0.7 across CV split. **d** Top 50 GO terms and pathways, ranked by adjusted p-value, highlight alterations in immune processes, extracellular matrix, angiogenesis and ERK1/2-MAPK signaling in the endometrium of women with endometriosis. Terms sizes <10 and >500 are excluded. Statistical significance was determined using a one-sided hypergeometric test, with multiple testing correction (g:SCS method). **e** Bar plot of cell types with >25% of cells among the top 10% ScaiVision cell filter scores predicting absence of endometriosis. Enriched populations include mural cell subsets (VSMC, Prv-CCL19, Prv-MYH11, Prv-STEAP4), ciliated epithelial cells, and myeloid cells (Mϕ1, Mϕ3, Mϕ5). **f** Box plot of confirmation AUCs (*n* = 50 per CV split) for the 11-gene signature derived from the intersection of ScaiVision-derived endometriosis signatures (PCA, CorrgPCA) and the top 300 cell-type-wise DEGs. **g** Representative CXCL3 immunohistochemistry stainings (scale bar: 0.2 mm): (1) eutopic endometrium from endometriosis-free donor, (2) eutopic endometrium from donor with endometriosis, (3) rectal endometriosis lesion, (4) ovarian endometrioma. In endometrium, arrowheads indicate epithelial glands with darker CXCL3 staining in ENDO versus non-ENDO. In lesions, arrows highlight epithelial cells with strong CXCL3 expression. Box plots in b, c and f show: center line, median; box limits, 25th–75th percentiles (IQR); whiskers, most extreme points within 1.5×IQR; points, individual AUCs.

To remove sources of confounding, ScaiVision models were trained exclusively on proliferative phase samples (Supplementary data 1). Employing a nested 5-fold Monte Carlo cross-validation scheme^66^, samples were first split in a stratified manner into 5 independent held-out evaluation sets, each containing 20% of the total samples. Within each of these splits, the remaining, non-held-out samples were further sampled 5 times using stratified Monte Carlo sampling in order to generate a total of 25 training (56% of samples) and validation (24% of samples) sets, which were used to monitor training and best model selection for evaluation (Fig. 4a). All cross-validation folds were balanced as far as possible with respect to endometriosis status, disease severity and sequencing batch (Supplementary Fig. 6a-c). We used four different feature selection methods to reduce the dimensionality of the data prior to model training: 1) PCA, identifying components of the highest variance^67^, 2) CorrgPCA, using correlation-based PCA (Methods), 3) diffgen, based on a non-parametric Wilcoxon rank sum test^68^ and 4) hvg, identifying highly variable genes^25^. All methods exhibited a median validation AUC of 0.78, with PCA, CorrgPCA, and diffgen achieving median validation AUC of ≥ 0.78 in 3 of the 5 inner CV folds (Fig. 4b top, Supplementary data 6). The top three models based on validation performance from each inner CV fold were then evaluated on the held-out datasets from each outer fold, resulting in median evaluation AUCs ≥ 0.78 in 3 of 5 folds for PCA and 2 of 5 folds for corrgPCA (Fig. 4b bottom, Supplementary data 6). Our presented models demonstrate equal efficacy in predicting both mild and severe endometriosis, highlighting robustness across different endometriosis severities (Supplementary Fig. 6d).

Next, we extracted the genes responsible for driving the performance of the top network from each fold, defined as having the lowest overlap of the sample prediction distributions (see Methods), from the well-performing PCA and corrgPCA feature selection methods based on deepLIFT and integrated gradients importance scores (see Methods)^69,70^. The unified PCA (215 genes) and CorrgPCA (210 genes) signatures shared over 60% of genes (PCA∩CorrgPCA), validating the selection process of predictive genes (Supplementary Fig. 6e, Supplementary data 7). To confirm the importance and sufficiency of these genes for the prediction of endometriosis, we trained additional ScaiVision networks with only the genes from each of the three signatures (PCA, CorrgPCA and PCA∩CorrgPCA) as input features. All three signatures achieved consistently high AUCs across 3 Cross-Validation (CV) splits of the proliferative samples, with the median network performance across splits achieving an AUC of 0.75, 0.83 and 0.83, respectively (Fig. 4c, Supplementary data 6). This result confirms that the ScaiVision networks can effectively prioritize the most important genes for predicting endometriosis in this dataset.

Subsequently, we aimed at predicting endometriosis based on only a subfraction of cells, which could be experimentally isolated from the endometrial tissue. In the proliferative phase samples, endometrial fibroblasts with high G2M values (cycling fibroblasts) account for 11.6% of the endometrium (Supplementary Fig. 7a). There are 32% more cycling fibroblasts in ENDO (13.0%) than non-ENDO (9.8%) (non-significant), in line with a previous study^18^. The ScaiVision models trained on only cycling fibroblasts demonstrated the potential to predict endometriosis, achieving an evaluation AUC of ≥ 0.78 on at least 3 of the 5 folds for diffgen, corrgPCA, and PCA dimension reduction methods (Supplementary Fig. 7b, c and Supplementary data 6). The united PCA, CorrgPCA, and PCA∩CorrgPCA signatures from the cycling fibroblasts achieved high performance, with median AUCs of 1.0 across all 3 Cross-Validation (CV) splits (Supplementary Fig. 7d, Supplementary data 6 and 9). GO term analysis of the gene signatures derived from these networks revealed enrichment of structural terms (collagen and ECM), apoptosis, myeloid cell differentiation, growth factor binding, MHC and responses to TNF (Supplementary Fig. 7e). These data emphasize the predictive potential of ScaiVision for endometriosis in only a fraction of endometrial cells, additionally highlighting the importance of cycling fibroblasts in endometriosis.

In conclusion, we successfully trained predictive models for endometriosis based on all cells or on cycling fibroblasts only and isolated core biomarker gene signatures with high discriminative value.

### ScaiVision predicts endometriosis based on multiple cell types and genes in immune pathways and angiogenesis

The unified PCA gene signature from all cells (Supplementary data 7), analyzed via Gene Ontology (GO) terms, highlights altered cytokine and chemokine activity, response, binding and signaling pathways (Fig. 4d). Additionally, chemotaxis and migration of various immune cell types were enriched (Fig. 4d), underscoring endometriosis-related immune system changes in endometrial tissue, in line with the DEG and receptor-ligand analysis (Fig. 3b, c). Regulation of angiogenesis and vasculature development were among the top 25 enriched GO terms (Fig. 4d), in line with the DEG and receptor-ligand analysis (Fig. 3b, h). Furthermore, collagen-containing extracellular matrix is top-ranked (rank 3, Fig. 4d), in line with the DEG pathway enrichment analysis (Supplementary data 5). The key genes in the unified PCA, ranked by integrated gradients importance scores (Supplementary data 7), include *IL1B*, *CCL4*, *CCL3*, *HLA-DRA*, *HLA-DPA1*, *CD74*, *A2M*, and *ACKR1*, which play crucial roles in immune responses, while *DUSP2* regulates MAPK signaling through dephosphorylation.

To further interpret the predictions made by ScaiVision models, we utilized their ability to rank cells by predictiveness, thereby identifying cell subsets associated with disease status. ScaiVision identified mural cells, macrophages, and ciliated epithelial cells to be important for non-ENDO prediction and mural cells, dendritic cells, and several epithelial cell types as most important for ENDO prediction (Fig. 4e, Supplementary data 8). This is consistent with the many altered gene expression changes and ligand-receptor interactions between myeloid, epithelial, and endothelial cells (Fig. 3), as well as increased VEGF signaling in mural cells (Fig. 3h) with subsequent increased cell frequency (Fig. 2f, Supplementary Fig. 4b).

Intersecting the ScaiVision-derived PCA and corrgPCA signatures from all cells with the top 300 cell-type-wise DEGs yielded an 11-gene signature (*CCN1*, *SLPI*, *DIO2*, *CEBPD*, *ADAMTS1*, *CALD1*, *CXCL3*, *CXCL2*, *COL4A2*, *TPM1* and *MGP*, ordered by predictive contribution). ScaiVision models based on this 11-gene signature achieved high AUC for endometriosis prediction (median AUC = 0.83, Fig. 4f). Each gene in this signature plays a differentiated role in biological processes. CCN1, with the highest predictive contribution (Supplementary Fig. 6f), promotes adhesion, migration, proliferation, and angiogenesis. SLPI protects against serine proteases, while DIO2 regulates thyroid hormone activity, previously linked to endometriosis^71^. The transcription factor CEBPD acts in immune responses. ADAMTS1 has inflammatory and anti-angiogenic properties and is involved in extracellular matrix degradation. CALD1 regulates smooth muscle contraction, and TPM1 controls muscle contraction. COL4A2 is involved in tissue remodeling, and MGP inhibits ectopic tissue calcification. The chemokines CXCL2 and CXCL3, which exhibit increased interaction with ACKR1 in ENDO (Fig. 3f), play a key role in immunoregulation. Immunohistochemistry results validate the elevated expression of CXCL3 in the epithelial cells of ENDO endometrium and reveal an even more pronounced expression in endometriosis lesions (Fig. 4g, Supplementary Fig. 8). This refined signature highlights cellular processes relevant to the pathogenesis of endometriosis, which may become further amplified within lesion sites, and suggests potential targets for future research.

## Discussion

The endometrium is a fundamental component of the female reproductive system for embryo implantation and growth. Anomalies in endometrial function are associated with multiple reproductive disorders, including endometriosis, a prevalent and debilitating disease^1,7^. The eutopic endometrium is thought to be the tissue of origin for endometriosis^8^, yet whether disease-related alterations can already be detected in the eutopic endometrium of affected women, or whether they only arise in the peritoneal cavity, has remained controversial^10^.

Because the endometrium undergoes rapid and cyclical remodeling, gene expression varies profoundly across menstrual phases. Without phase stratification, disease-related differences are easily masked or misinterpreted as physiological variation, a caveat that has hampered earlier analyses. Our dataset provides a cycle-resolved reference of cellular states and intercellular interactions across the endometrium, enabling the detection of robust endometriosis-specific signatures that were previously inaccessible.

Here we present a comprehensive single-cell atlas of the eutopic endometrium comprising 60 donors (35 with and 25 without endometriosis): (1) without exogenous hormonal treatment, (2) collected across different menstrual cycle phases, (3) analyzed with stratification by the donor’s menstrual cycle phase, (4) employing whole single-cell sequencing, (5) processed uniformly, and (6) accompanied by well-defined clinical data (see Supplementary data 10 for comparison with previously published studies). Even though previous single-cell studies on endometriosis provided valuable insights^16–21,47,72^, comparison of eutopic endometrium between endometriosis and non-endometriosis donors have remained limited by small eutopic sample sizes (n ≤ 3 per cohort)^18,19^, inclusion of hormonally treated and/or postmenopausal donors^18,19^, restricted cell subset analyses^21,47,72^ and/or lack of stratification by the donor’s menstrual cycle phase^18,72^.

Our approach overcomes the major limitations of prior work with a large sample size (*n* = 60), exclusion of hormonally treated and postmenopausal donors, comprehensive single-cell annotation and analysis, and robust stratification by the donor’s menstrual cycle phase. By addressing these limitations, we identified significant gene expression changes and altered receptor-ligand interactions in the eutopic endometrium of endometriosis patients, which potentially contribute to lesion formation, deepening our understanding of endometriosis pathophysiology. Leveraging these data as input to advanced neural network models in the ScaiVision platform, we can effectively predict endometriosis based on endometrial biopsies.

Studying the endometrium poses specific challenges due to its monthly renewal cycle, accompanied by expression changes of hundreds of genes^24^. Endometrial maturation is initiated by endometrial epithelial progenitor and mesenchymal stem cells in the basal layer, which proliferate under estrogen to repopulate the functional layer^73^. The rise of progesterone during the periovulatory and secretory phases prompts significant functional and morphological changes, as well as lineage specialization of the epithelial cells and decidualization of the stromal cells^74^. Dynamic immune cell infiltration supports this process, aiding tissue function transition and angiogenesis^30^. Our atlas, with refined menstrual cycle evaluation, detailed cell annotation, and high cell count per donor, maps cellular content and transcriptional changes across the menstrual cycle. It will serve as a crucial resource for an improved understanding of endometrial development, describing changes in cellular content, maturation, and intercellular relationships throughout the stages of the endometrial cycle. This insight is pivotal for comprehending how abnormalities in endometrial maturation may contribute to endometrial pathologies.

During the menstrual cycle, key mechanisms and pathways undergo dynamic changes, including angiogenesis/VEGF^75^, immune functionality^24^, wound healing^30^, adhesion^76^, WNT and NOTCH^12^ and TGFb signaling^12,77^. Exogenous hormonal treatment has varied effects on menstrual cycle phase markers rather than achieving synchronization (Supplementary Fig. 3g, h). Therefore, it is essential to stratify endometrial analysis by the donor’s menstrual cycle phase and ensure an adequate number of donors and cells for accurate interpretation of disease-specific changes, considerations often overlooked in previous studies.

**Fig. 5:**
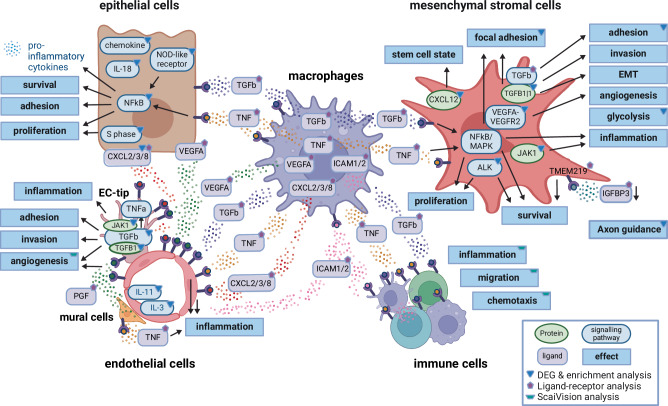
Graphical summary of enhanced ligand-receptor interactions and pathways in the eutopic endometrium of women with endometriosis. Ligand-receptor analyses point toward a pivotal role for macrophages in endometriosis endometrium. Enhanced interactions between macrophages and various endometrial cell types, mediated by TNF, CXCL, TGFb, ICAM and VEGF signaling, indicate elevated inflammation, angiogenesis, proliferation, and cell survival. Additionally, elevated signaling between epithelial and endothelial cells via CXCL and VEGF pathways was observed. Functional enrichment analysis of differentially expressed genes (*n* > 400), stratified by cell-types, underscores significant perturbations in inflammation (chemokine, NFκB and NOD-like receptor signaling), cellular adhesion (TGFb receptor signaling, adhesion, extracellular compartment), cell proliferation and survival (NFκB signaling, ALK signaling), angiogenesis (VEGF and TGFb signaling), and nervous system processes (development and axon guidance) across multiple cell types. Furthermore, ScaiVision analysis underscores significant changes in cytokine and chemokine activity, chemotaxis and migration of various immune cells and angiogenesis. These findings are consistent with the processes believed to be crucial for endometrial cells to establish viable ectopic lesions. In conclusion, the generation and analysis of our single-cell atlas reveals at unprecedented cell-type resolution that regulatory disturbances are present already in the eutopic endometrium of women with endometriosis, potentially increasing endometriosis susceptibility and suggesting therapeutic targets for intervention. *Created in BioRender. Duempelmann, L. (2026)* https://BioRender.com/2k0nx91.

This strategy enabled us to discover hundreds of differentially expressed genes across various cell types between non-ENDO and ENDO donors, all within the morphologic and transcriptionally homogeneous proliferative phase of the menstrual cycle^11,24^. The added precision of examining gene expression at the single-cell level underscores the advantages of single-cell sequencing in studying this complex tissue. Beyond confirming previously implicated pathways, such as TNF, VEGF, and TGFβ signaling, our analysis provides unprecedented resolution by identifying the specific cell types, ligands, and receptors mediating these interactions (Fig. 5 provides a graphical summary). For example, we uncover macrophage subtype–driven TNF–TNFR1/2 signaling to structural endometrial cells and identify adhesion axes, such as ICAM1/2–(ITGA* + ITGB2)/SPN between immune and epithelial cells, and cell death resistance mechanisms involving reduced TMEM219–IGFBP3 signaling in mesenchymal cells. We also present a detailed receptor-specific map of VEGF signaling, showing macrophage- and mural cell–endothelial interactions that align with secretory mural cell expansion in ENDO, offering in situ support for the ‘stem cell hypothesis’ of pathogenesis. In addition, our data highlight axon-guidance–related pathways supporting nerve fiber infiltration in the endometrium and subtle increases in WNT/NOTCH pathways affecting epithelial differentiation. A structured comparison of our findings with previous reports is provided in Supplementary data 10, which highlights both the known pathways we corroborate and cell-type-specific interactions revealed by our single-cell approach. These insights suggest that inflammation, adhesion, survival, angiogenesis, and epithelial differentiation are already perturbed in the proliferative eutopic endometrium of women with endometriosis, providing early molecular signatures of disease initiation.

Endometriosis affects approximately 190 million women globally, causing significant disability and negatively impacting quality of life. A reliable in vitro diagnostic could reduce the economic burden on healthcare systems, estimated to be over $10,910.96 per woman per year^2^, while also minimizing diagnostic delays that lead to increased costs and inadequate treatment. The predictive models trained in this study lay the groundwork for future development and clinical validation of such a diagnostic assay, while demonstrating the potential of leveraging tailored machine learning algorithms to unravel complex biological signatures. The gene signatures derived from these models can serve as a basis for both hypothesis generation regarding potential mechanisms and therapeutic targets for endometriosis, as well as prioritizing biomarkers for further diagnostic development. Mild endometriosis is particularly hard to detect through clinical examinations or conventional biomarkers^5^. Our single-cell-based models, however, can predict both mild and severe endometriosis with similar sensitivity, highlighting the sensitive nature of single-cell techniques. While single-cell sequencing remains expensive and challenging to implement clinically, emerging technologies, such as the 10X Flex Gene Expression kit and Parse Biosciences Evercode kit aim at streamlining and standardizing these approaches, making them more accessible. Furthermore, our ongoing efforts focus on validating our signatures in bulk RNA-seq data from independent donor cohorts, as well as distilling our best-performing single-cell signature through iterative gene selection into a core gene set suitable for a more cost-effective qPCR approach and large-scale clinical validation study. In conclusion, our research offers a significant step forward in understanding endometriosis at the single-cell level, providing a foundation for improved diagnostics and targeted therapies.

## Methods

### Study approval and donor cohort

All procedures involving human participants were conducted in accordance with the ethical standards of the Swiss Ethics Committee and the 1964 Declaration of Helsinki and its later amendments. The study was approved by the Swiss Ethics Committee (KEK-BE 01780, 2019). Donors provided written informed consent prior to inclusion and participated without receiving compensation. Inclusion criteria for this study included a minimum age of 18 years and scheduled laparoscopic surgery for indications, such as symptoms of endometriosis, tubal ligation, salpingectomy, hysterectomy, idiopathic infertility, pelvic pain or other gynecological pathologies as part of their planned clinical treatment. Donors with pre-existing inflammatory diseases, malignancy, pregnancy, lactation or the onset of the last period more than 35 days before the surgery were excluded. Donors without endometriosis but with adenomyosis were excluded. Donors filled out a pain questionnaire before laparoscopy. Immediately before surgery, an endometrial biopsy was obtained using Pipelle-de-Cornier (Laboratoire CCD, 1103000) and peripheral blood was taken with an S-Monovette tube (Sarstedt, 04.1926.001). During laparoscopy, each donor’s pelvic cavity was visually inspected for endometriotic lesions. Endometriosis was confirmed for each endometriosis patient in this study by histopathological evaluation.

### Tissue processing for single-cell RNA capture

After removal from the uterus, endometrial biopsies were immediately processed into single-cell suspensions and cryopreserved. Briefly, each endometrial biopsy was washed twice in PBS, cut into small pieces with a scalpel, and digested in 5 ml of IMDM (Gibco, 31980022) supplemented with 1 mg/ml Collagenase type I-A (Sigma-Aldrich, C2674) and 1 mg/ml Collagenase type II (Worthington, LS004174) at 37 °C for 30-60 minutes. During digestion, the tissue-containing tube was inverted several times every 10 minutes until tissue pieces appeared visually dissociated. Enzymatic dissociation was terminated by the addition of 5 ml 4 °C PBS. Cells were mechanically dissociated in a gentleMACS™ C Tube (Miltenyi Biotec, 130-093-237) using the gentleMACS™ Dissociator (Miltenyi Biotec, 130-093-235), with the Program mLung 02.01. The resulting cell suspension was filtered through a 100 µm and 40 µm strainer (Corning, 352340 and 352360) and rinsed with an additional 30 ml 4 °C PBS. To remove red blood cells, cell suspension was pelleted by centrifugation (300 g, 10 min), suspended in 6 ml of 1x red cell lysis buffer (Invitrogen, 00-4300-54), and incubated for 5 min at room temperature. The lysis was stopped by the addition of 35 ml 4 °C PBS and endometrial cells were filtered using a 20 µm strainer (pluriSelect, 43-50020-01). Following centrifugation (300 g, 10 min), the cells were cryopreserved in 90% FCS (Sigma-Aldrich, F7524) 10% DMSO (Sigma-Aldrich, D8418), frozen in a Bicell freezing vessel (Nihon Freezer, 1-6263-01) at −80 °C and subsequently stored long term in liquid nitrogen. For this project, 330 endometrial biopsy samples, each from a unique donor, were dissociated to single-cells and cryopreserved between 5/2020-12/2021.

### Single-cell RNA capture with 10x Genomics and sequencing with Illumina

Samples for 10x capture were selected by a thorough review of clinical data, pathology reports, menstrual cycle information, endometrial biopsy scores and single-cell quality. Selected endometrial single-cell aliquots were thawed in a 37 °C water bath and suspended in 37 °C IMDM supplemented with 10% FCS. After centrifugation (300 g, 5 min), cell suspension was washed with 4 °C PBS + 0.2% BSA (Sigma, A4503). After centrifugation (300 g, 5 min), the cell pellet was carefully resuspended in 4 °C PBS + 0.2% BSA to a concentration of ~1*10^6 cells/ml and strained using a 35 µm nylon mesh (Corning, 352235) or a 40 µm Flowmi cell strainer (Sigma-Aldrich, BAH136800040) to ensure the absence of cell aggregates. Cell concentration and viability (propidium iodide-negative population) were measured after 5 min incubation of cell aliquot in 2 µg/ml propidium iodide (Sigma, 81845) using the portable flow cytometer Moxi Flow (ORFLO Technologies, cassette Typ MF-M, MXC021). Only samples with a post-thawing viability of >70% were included for single-cell capture. Most tissue samples were fully consumed during this process and could not be preserved for further use.

Single-cell capture and cDNA library generation were performed with the 10x Genomics Chromium Single Cell Gene Expression workflow (Chromium NextGEM Single Cell 5’ Library and Gel Bead Kit v1.1, Chromium Controller). 25000 viable cells per sample were captured in a single reaction. cDNA libraries were balanced by shallow iSeq and sequenced with a NovaSeq 6000 System (Illumina, S4, 2 ×100 cycles).

### scRNA-seq data processing and atlas generation

The workflow was embedded in the workflow management engine Snakemake^78^ (v5.14) for automation and to ensure reproducibility. The software MultiQC^79^ was used for the quality control of the raw reads. Gene indexing, cell debarcoding, deduplication, read mapping, and estimation of transcript-level expression by pseudo-alignment were performed with the Salmon software package AlevinQC^80^ (v1.6.0). The Seurat^25^ (v4.1.0) and scater^81^ (v1.18.0) packages were used to perform quality control and visualization of the data on the sample-, cell- and gene-level. Included are the detection and removal of outlier cells based on transcript and gene metrics, the detection of possible doublet cells, and batch effects. Samples not fulfilling the following quality criteria were excluded: median cell-wise mitochondrial expression <30%, median number of genes per cell > 1000, and median number of unique transcripts per cell > 1000. Dimension reduction was done by selecting the 3000 highly variable genes that account for the most variation in a cell population.

### Principal component analysis

Sample-wise Principal Component Analysis (PCA) was performed on the sample-wise aggregated integrated transcripts^25^ with PCAtools (v2.10.0).

### Menstrual cycle phase-specific marker genes

Mesenchymal or epithelial cells were subsetted from the entire single-cell atlas and samples 65 and 105 were excluded, due to their possible transitional state between periovulatory and secretory menstrual cycle phases. Menstrual cycle phase-specific markers were identified with the FindAllMarkers function from Seurat (v5.0.3) and filtered for adjusted p-value < 0.05 and avg_log2FC > 2.

### Cell-type identification

Cells were annotated with Symphony (v0.1.1) using Tan et al.^18^ as reference. First, the entire dataset was annotated with the coarse annotation from Tan et al. Any annotations with a probability below 0.5 were discarded. Second, the entire dataset was split into the major cell types (mesenchymal, epithelial, endothelial, myeloid, and lymphoid) and subsequently annotated with the refined annotation from Tan et al^18^. Any annotations with a probability below 0.5 were discarded. The marker genes of the cell types were confirmed with DotPlot^25^. Graph-based clustering with the Louvain algorithm with multilevel refinement unveiled endometrial fibroblasts, C7 fibroblasts, and mesenchymal mural cell clusters specific to the menstrual cycle phase.

### Time trajectory of the cycle phase with monocle3

Time trajectory analysis was performed on the re-integrated epithelial cells with exclusion of ciliated, mesothelial, and MUC5B+ cells. Trajectories were deduced using the learn_graph function in the Monocle 3 v3_1.3.4 R package^82–84^, with a geodesic_distance_ratio set to 0.27. The root of the trajectory was determined by identifying the graph node close to cells from donors with the early menstrual cycle days 5, 6 and 7. Subsequently, cells were ordered along pseudotime using Monocle 3.

### Menstrual cycle phase evaluation

The menstrual cycle phase of each donor was assessed from serum progesterone levels, cycle day, histological endometrium evaluation (if available) by two independent pathologists, and the transcriptional profile. Samples from women without measurable serum progesterone ( < 2nmol/l) and menstrual cycle day ≤ 15 were considered to be in the proliferative menstrual cycle phase. Samples from women with measurable serum progesterone (>2 nmol/l), which clustered together with proliferative samples in PC1/PC2 and the UMAPs of all cells and epithelial cells, were considered as periovulatory and samples that clustered separately as secretory. From the transcriptional profile, proliferative and periovulatory samples could not be distinctly identified. The evaluation of secretory into early-, mid- and late-secretory was performed under the consideration of the pathologist’s evaluation (if available) and the transcriptomic profile. Samples with the majority of stromal cells being in the dS3 cell cluster (separate cluster in UMAP of all cells) were considered as early- and mid-secretory, with a clear separation between early- and mid-secretory cells in the UMAPs of all cells or epithelial cells. The late-secretory stromal cells clustered completely separately in the UMAP of all cells with high frequencies of dS4 cells. This separation corresponds to the phase marker genes described in Wang et al. 2020. Marker genes for all 5 major cell types and each menstrual cycle phase are provided in Fig. 2d and Supplementary data 2.

### Differential gene expression and pathway enrichment analysis

Cell-type-wise differential expression analysis, comparing non-ENDO to mild-, severe- or all-ENDO, was performed with Muscat (bioconductor-muscat v1.12.0) by fitting cell-level models on the RNA assay^28^ with 10x capture batch as covariate. Samples from the proliferative phase with strict exclusion criteria were used as input (Supplementary data 1). Only cell-types were considered for testing with at least 3 ENDO and 3 non-ENDO samples with at least 50 cells per sample. Cell-types too small for DEG testing were, if possible, aggregated with biologically similar cell-types (e.g., pNK, NK1, NK2, NK3 to NK, Supplementary data 3). The cut-off for differential expression was set at a p-value local adjusted of smaller than 0.05 and average expression > 18.53202 (inflection point), with average expression as calculated by Muscat. Ribosomal genes were excluded. Pathway enrichment analysis was performed with gProfiler^85^. Differential expression results obtained using Seurat FindMarkers() are additionally provided in Supplementary data 4. Fold changes and p-values are not directly comparable between Muscat and Seurat because the methods use different statistical frameworks for differential expression testing.

### Ligand-receptor interaction analysis

Ligand-receptor analysis was performed using the R package CellChat^29^ (v2.0.0) with standard settings. First, the cell atlas was filtered for the proliferative samples with strict exclusion criteria (Supplementary data 1) and then split into ENDO and non-ENDO Seurat objects. These were independently converted into CellChat objects for initial data exploration. Subsequently, the two CellChat objects (ENDO and non-ENDO) were merged, and interaction patterns were compared and visualized using several different CellChat functions. Ligand-receptor interaction findings were cross-referenced with differentially expressed genes in Supplementary data 4.

### Cell cycle phase

Cell-cycle scores were assigned to mesenchymal cells with the Cell-Cycle vignette from Seurat^25^, based on the G2/M and S phase markers genes^86^. Two mesenchymal cell clusters (Seurat clusters 2 and 4) contained cells with G2/M and S cell-cycle scores above 1 and were considered as cycling mesenchymal cells.

### Immunohistochemistry for CXCL3

Human endometrium and endometriosis lesion tissues were fixed in 4% Formalin and embedded in paraffin (FFPE). Tissue sections (2.5 μm) were stained with anti-CXCL3 antibody (Biorbyt, ORB13448, 1:500 dilution) and processed on the automated research stainer Leica Bond RX (Leica Biosystems), using heat-induced epitope retrieval (BOND Retrieval Solution 1 AR9961, pH 6, 100 °C, 30 min**)** and BOND Polymer Refine Detection (DS9800). Stained slides were scanned at 40x magnification (Pannoramic 250 scanner, 3DHistech). All processing was performed by the Translational Research Unit, Institute of Tissue Medicine and Pathology, University of Bern, Switzerland, in a blinded manner.

### ScaiVision analysis

Endometriosis predictions were carried out using the interpretable neural network algorithm CellCnn^65^ (v0.2), implemented in PyTorch (v1.10.2) in the ScaiVision platform (v1.6.3, Scailyte AG), similar to Roussel et al^87^. Briefly, this is a supervised machine learning algorithm that trains a convolutional neural network with a single hidden layer to predict sample-level labels using single-cell data as inputs.

The scRNA data of the proliferative, no progesterone samples with more than 5000 cells per sample (Supplementary data 1) were included in the ScaiVision network training, using either all cells or only cycling fibroblast cells^25,86^. Each sample was assigned a label indicating the donor's Endometriosis status (Endometriosis or Control). For model training, we used a nested cross-validation scheme. In the outer loop, we followed a 5-fold Monte Carlo cross-validation (MCCV) scheme in which ~20% of samples were set aside for evaluation for each fold, resulting in 5 independent evaluation datasets. The remaining samples ( ~ 80%) of each fold were further allocated to 5 MCCV splits of training (70%) and validation (30%) in a stratified manner to maintain the relative proportions of each class (technical batches and endpoints non-ENDO/ENDO), resulting in 25 training and validation datasets. This inner loop was used for the selection of the best network to be applied to the evaluation dataset.

Four different feature selection methods were used to reduce the dimensionality of the data prior to model training: 1) PCA^67^, 2) CorrgPCA 3) differentially expressed genes (“diffgen”)^68^, and 4) highly variable genes (“hvg”)^88^. CorrgPCA, Scailyte’s proprietary dimension reduction technique, first groups the highest variable genes^88^ based on pairwise correlation. It then performs a principal component analysis on each gene group separately and selects the 200 components that capture the highest amount of variance.

Per training dataset and feature selection method, 50 ScaiVision models with varying hyperparameters were trained on the dimension-reduced training data. To generate input data for training ScaiVision, sub-samples of 200 cells, termed multi-cell inputs (MCIs), were chosen randomly from each sample independently. For each training epoch, 1000 MCIs from each label class (ENDO or non-ENDO) were presented to the network in random order. 50 independent networks were generated for each feature selection method using hyperparameters randomly chosen from the following options: i) number of filters: (3, 5, and 10), ii) top-k pooling percentage: (20), iii) dropout probability: (0.3, 0.4, and 0.6), iv) learning rate: (0.001, 0.003, and 0.01), and v) weight decay: (0.00001, 0.0001, 0.001, 0.01, and 0.1). Training was performed with a batch size of 64. Each network was trained for a maximum of 50 epochs, or until the validation loss stopped decreasing for 20 consecutive epochs. At the end of the training, the weights from the epoch with the lowest validation loss were returned. The loss is computed on the probabilities returned by the models to belong to the class “ENDO”.

For each of the outer MCCV folds and each feature selection method, 3 trained models were selected that performed best on their respective validation dataset. For the selection of the best models, an average rank was computed across two metrics: First, the binary cross-entropy loss. Second, the overlap between the output probability distributions for each class, each of which was computed by fitting a normal distribution to the model outputs for samples belonging to one class. Here, low overlap indicated better model performance. The 3 models with the highest average per feature selection method and fold were then selected for evaluation on the hold-out set. We continued our analysis with those feature selection methods for which the best selected models achieved an AUC ≥ 0.8 on at least 3 of the 5 folds.

Models derived from different feature selection methods use different sets of input genes; additionally, each model can learn different weights for each input feature. In order to derive a unified gene signature for PCA and CorrgPCA, the top network was picked from each of the 5-folds. Only networks that had an accuracy of 0.66 or above and an AUC of 0.78 or above on the held-out evaluation set were picked. In the case of ties, the network with the lowest overlap in the response distributions between endometriosis and control predictions was picked. Folds that did not have a single network satisfying these criteria were excluded from further analysis. The AUC obtained across all learners, cell selection and dimension reductions methods is described in Supplementary data 6.

Next, for each of the networks, importance scores were calculated using the deepLIFT^69^ and integrated gradients methods implemented in the Captum software^70^, providing a metric for the importance of each feature for driving the prediction of either endometriosis or control. Integrated gradients importance scores were averaged feature-wise across the deepLIFT and integrated methods and across all the samples within a group (endometriosis or control), and the absolute difference between the integrated gradients importance scores for endometriosis prediction and that for control prediction was calculated for each feature. Next, the top 20% of features, ranked by the mean absolute difference of integrated gradients importance scores, were selected for further consideration. For corrGPCA, each feature (CorrgPCs) was mapped back to the top 20% of genes associated with it (ranked by the absolute PC loading of each gene). If the feature had fewer than ten genes associated with it, then the top 2 genes were picked, and for singleton gene sets, the single gene was picked. Features associated with PCA were treated analogously, however, due to the non-sparse nature of PCs relative to corrGPCs, only the top 1% of genes were picked per integrated gradient importance score-ranked PC per feature. The gene signatures identified are listed in Supplementary data 7 and Supplementary data 9 for model training using all cells and cycling mesenchymal cells, respectively. Along the genes composing each signature, a separate column refers to the rank of each gene after sorting by the integrated gradient derived importance score.

To identify cells driving the prediction of our models, cells targeted by the models selected during the evaluation were examined. Each of the models contains different filters, which collectively form the model’s prediction. Combining each filter's weights with the expression measurements via a weighted average yields a score per filter. This score represents how strongly the cells respond to the filter. To pinpoint predictive cells throughout folds, we examined all the filters and called a cell type as predictive if it was consistently selected in different folds.

### Statistics and reproducibility

No formal sample-size calculation was performed. Of 330 participants with cryopreserved endometrial biopsies, we included most who met our stringent criteria. All data exclusion criteria were pre-established, and data was only excluded if it did not meet the quality criteria, as described in detail above. For cell type frequency, differential gene expression and ligand-receptor analysis stringent donor exclusion criteria were applied (Supplementary data 1), due to a potential influence of these clinical parameters. To compare cell type frequencies across menstrual phases, we utilized 23 samples from the proliferative phase, 18 from the periovulatory phase, and 13 from the secretory phase, adhering to stringent clinical exclusion criteria. Retrospective alignment with a menstrual cycle atlas showed perfect phase correspondence. For differential gene expression and ligand-receptor analysis, the 12 samples from women with endometriosis and 11 without endometriosis from the transcriptionally homogeneous proliferative menstrual cycle phase were used, with stringent clinical exclusion criteria applied, ensuring reliable results. H&E stainings of 35 endometrial samples were independently evaluated for menstrual cycle phase by two pathologists, yielding highly concordant assessments. CXCL3 immunohistochemistry stainings (Supplementary Fig. 8) show consistent staining patterns across the cohort, representative stainings are shown in (Fig. 4g)

### Reporting summary

Further information on research design is available in the Nature Portfolio Reporting Summary linked to this article.

## Supplementary information

Supplementary Information

Description Of Additional Supplementary File

Supplementary Data 1

Supplementary Data 2

Supplementary Data 3

Supplementary Data 4

Supplementary Data 5

Supplementary Data 6

Supplementary Data 7

Supplementary Data 8

Supplementary Data 9

Supplementary Data 10

Reporting summary

Transparent Peer Review file

## Data Availability

The data supporting the findings from this study are available within the manuscript and its supplementary information. All raw sequencing data and the processed Seurat object are available at NCBI’s Gene Expression Omnibus (series accession number: GSE266265; https://www.ncbi.nlm.nih.gov/geo/query/acc.cgi?&acc=GSE266265). Source data are provided with this paper.

## Code availability

Code used for data analysis in this study is available at: https://github.com/DuempelmannLea/endometriosis_endometrium_scRNA_atlas10.5281/zenodo.19278464.
