# Supplementary material for: Endometriosis-related alterations in the endometrium revealed by integrated single-cell and AI-powered approaches
Source: Nat Commun. 2026 May 20;17:6688. doi: 10.1038/s41467-026-73020-4 (PMC13385355; doi:10.1038/s41467-026-73020-4)
Supplement: Supplementary file 1 — Supplementary Information [file 41467_2026_73020_MOESM1_ESM.pdf]

Supplementary Information

Endometriosis-Related Alterations in the Endometrium revealed by Integrated Single-Cell and AI-Powered Approaches

Lea Duempelmann[1,2,*], Shaoline Sheppard[3], Brett McKinnon[1,2,4,†], Angelo Duo[3,†], Jitka Skrabalova[1,2,†], Thomas Andrieu[1,2], Ryan Lusby[3], Wiebke Solass[5], Dennis Goehlsdorf[3], Sukalp Muzumdar[3], Cinzia Donato[6], Hans Bösmüller[7], Sarah Carl[3], Peter Nestorov[3] and Michael D. Mueller[1,2,*].

[1] Endometriosis & Gynaecological Oncology Laboratory, Department for BioMedical Research, University of Bern, Murtenstrasse 35, 3008 Bern, Switzerland

[2] Department of Obstetrics and Gynecology, Inselspital, Bern University Hospital, University of Bern, Friedbühlstrasse 19, 3010 Bern, Switzerland

[3] Scailyte AG, Lichtstrasse 35, 4056 Basel, Switzerland

[4] Institute for Molecular Bioscience, The University of Queensland, Brisbane, QLD, Australia

[5] Institute of Tissue Medicine and Pathology, University of Bern, Murtenstrasse 31, 3008 Bern, Switzerland

[6] Hera Biotech, Inc., 1475 college park, San Antonio, TX 78249, USA

[7] Department of Pathology, University Hospital Tuebingen, Liebermeisterstraße 8, 72076 Tuebingen, Germany

*Corresponding authors: Lea.Duempelmann@unibe.ch, Michel.Mueller@insel.ch

†Equal contribution

Supplementary Figure 1

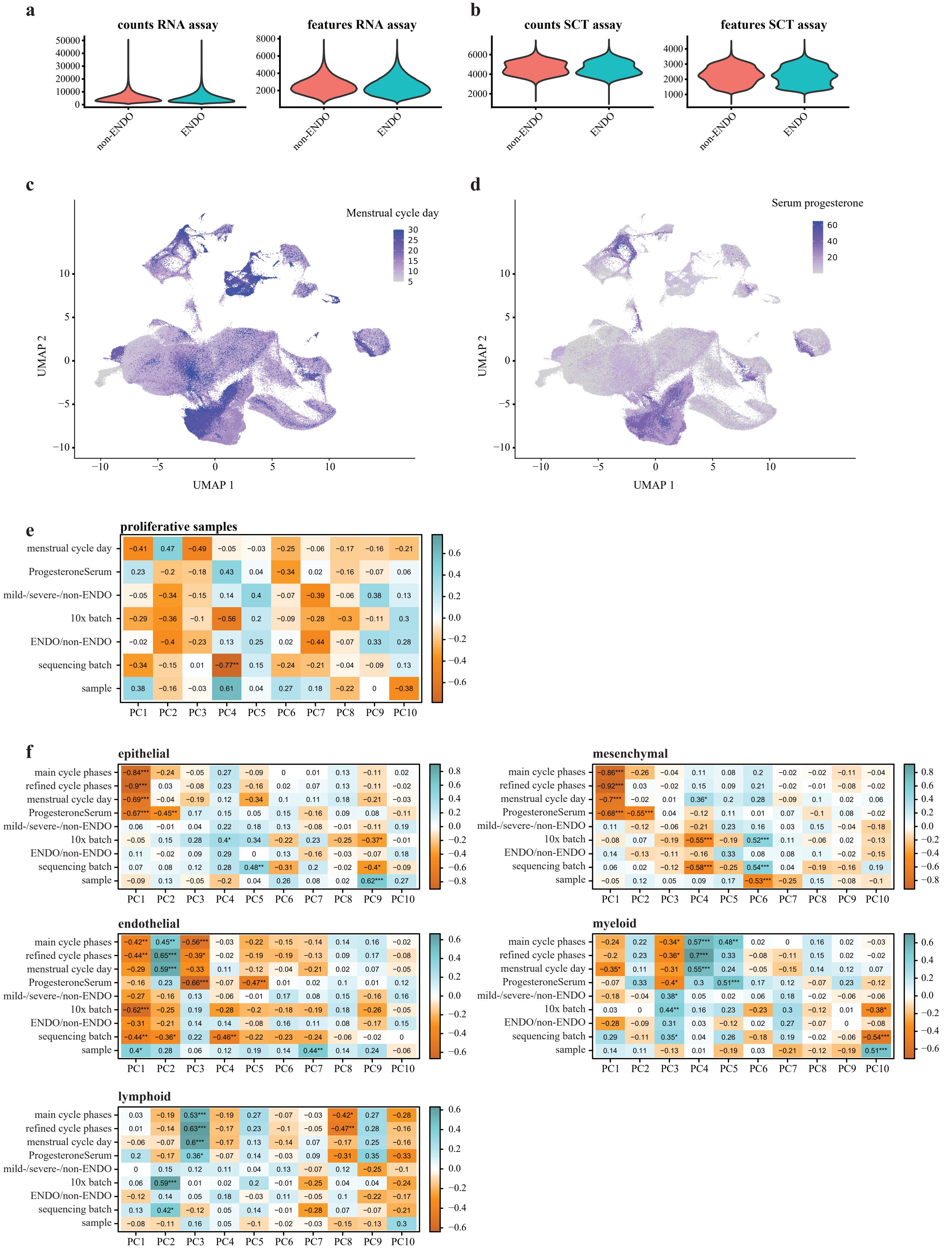

Supplementary Figure 1. **Additional parameters and features of the endometrium single-cell atlas.**

a, b Violin plots illustrating counts and features from RNA assay (a) and SCT assay (b), show no discernable differences between ENDO and non-ENDO.

c, d UMAPs of the entire endometrium single-cell atlas, colored by menstrual cycle day (c) and progesterone in serum (d), show a clear visual separation of cells by menstrual cycle day and serum progesterone concentration.

e Eigencor plot displaying Pearson correlation coefficients between the covariates and the principal components (PCs) of the proliferative samples with strict exclusion criteria (n = 23 samples), with significance assessed by two-tailed tests and adjusted using the Benjamini–Hochberg procedure. No significant correlation was identified between menstrual cycle phase parameters or endometriosis status and the initial ten PCs. Statistical significance symbols represent: *** <0.001, ** <0.01, * <0.05.

f Eigencor plot displaying Pearson correlation coefficients between the covariates and the principal components (PCs) of the main 5 cell types (epithelial, mesenchymal, endothelial, myeloid, lymphoid; of n = 60 samples), with significance assessed by two-tailed tests and adjusted using the Benjamini–Hochberg procedure. Menstrual cycle phase parameters significantly correlate with high PCs in all main cell types. In contrast, a significant correlation between endometriosis status and the first ten PCs was only detected in the myeloid cells among the major cell types. Statistical significance symbols represent: *** <0.001, ** <0.01, * <0.05.

Supplementary Figure 2

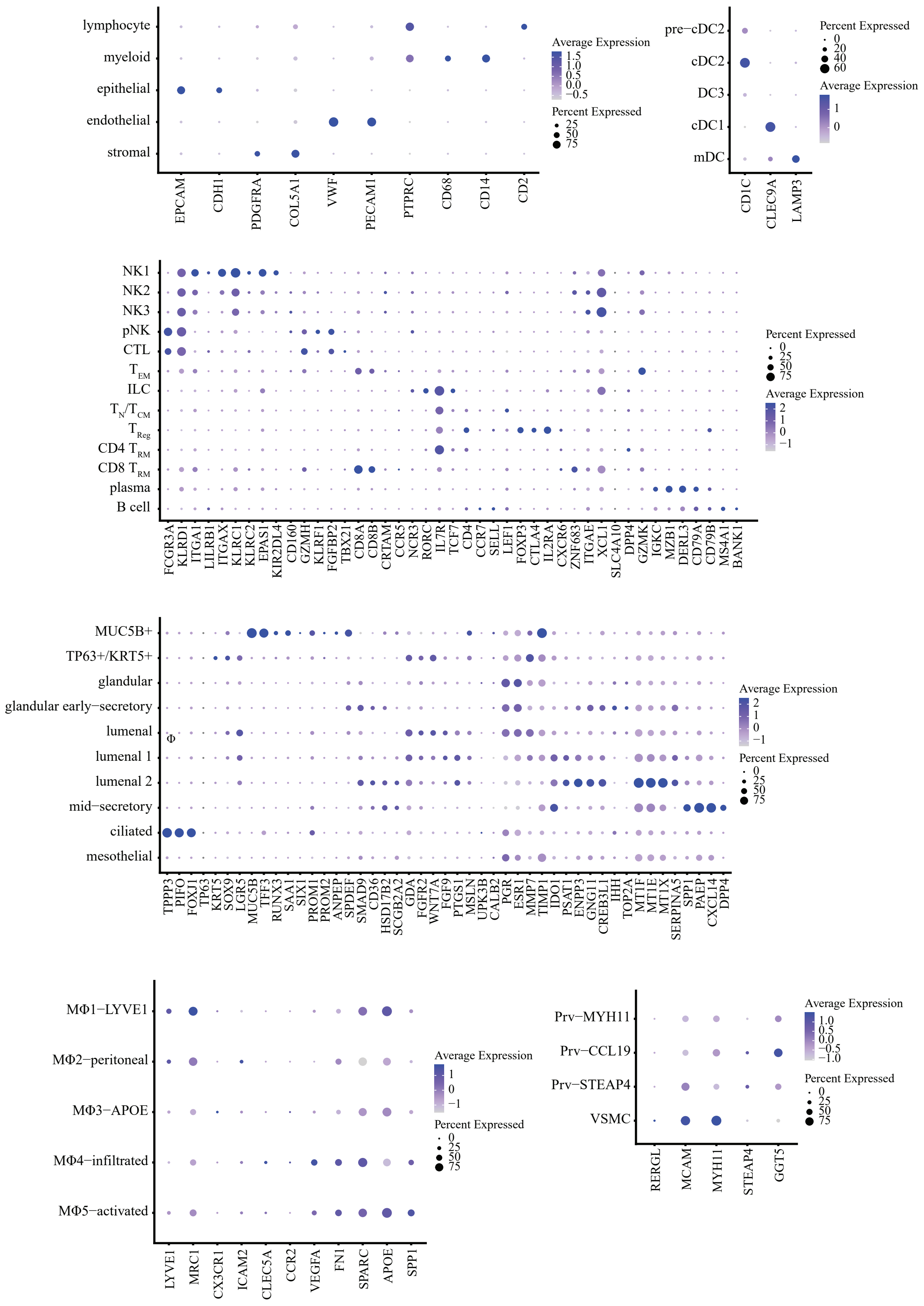

Supplementary Figure 2. **Marker gene expression from our dataset corresponds to the reference.** Expression of the marker genes for the different cell types corresponds to the cell type-specific expression shown in Tan et al. 2022.

Supplementary Figure 3

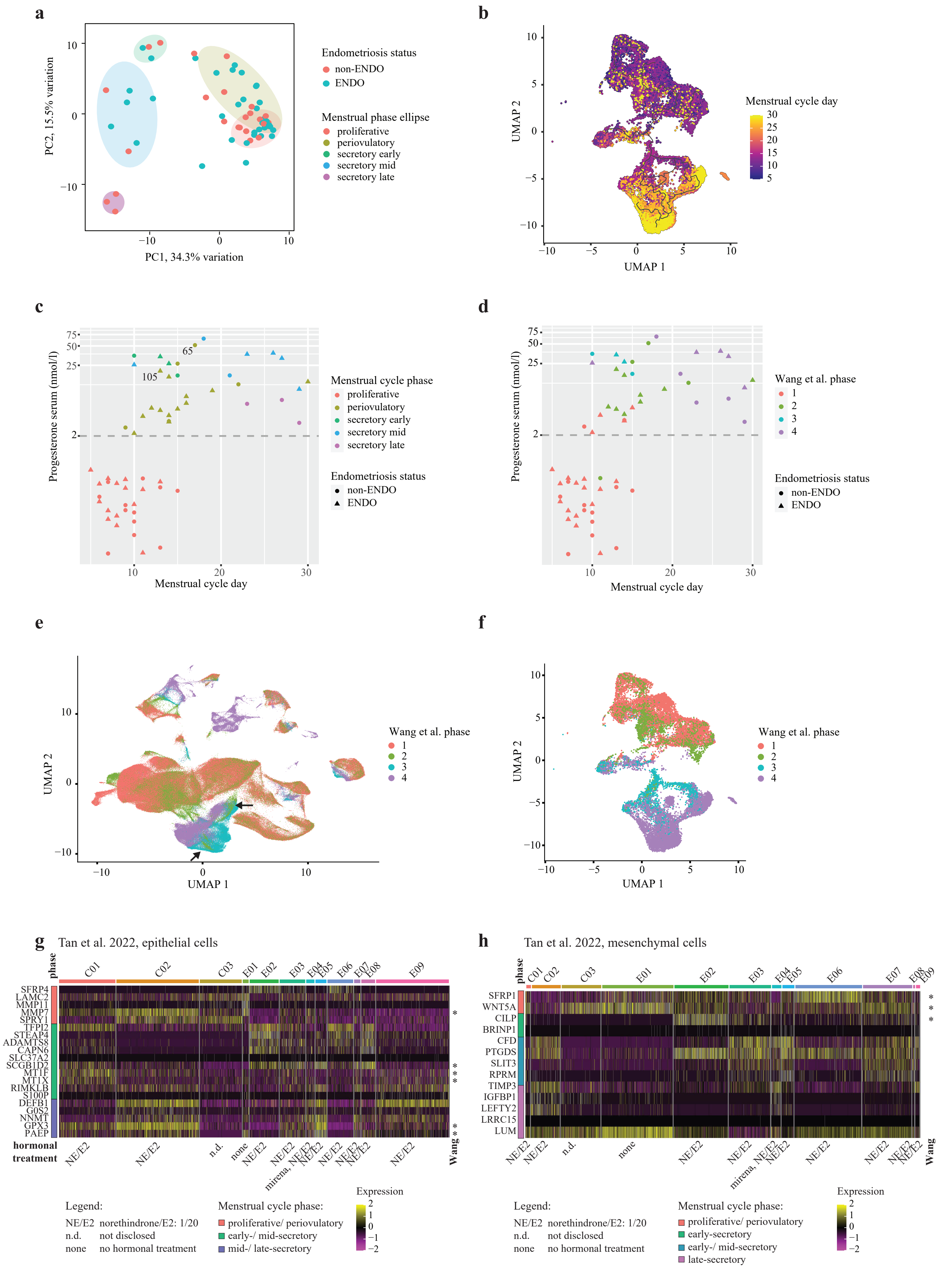

Supplementary Figure 3. **Strong correlation of menstrual cycle day and phase with pseudo time prediction and reference atlas.**

a Scores plot of the first two principal components PC1 and PC2 from sample-wise whole transcriptome PCA of the SCT assay, colored by endometriosis status. Ellipses mark the different menstrual cycle phases (same as in Fig. 2a). ENDO and non-ENDO do not cluster distinctively in any menstrual cycle phase.

b UMAP of re-integrated epithelial cells colored by menstrual cycle day. Progression from early to late menstrual cycle stages is evident and consistent with Monocle pseudo time prediction (Fig. 2c).

c, d Scatter plots showing the correlation of menstrual cycle day and serum progesterone with our menstrual cycle phase staging (c) and median Wang phases, transferred with Symphony (d). There is a perfect correspondence between our cycle phase evaluation and the mapped Wang phases (proliferative/periovulatory phase with Wang phases 1/2, early-secretory phase with Wang phase 3, and mid-/late-secretory phase with Wang phase 4).

e, f UMAPs of the entire dataset (left) and the re-integrated epithelial cells (right), colored by the Symphony transferred Wang phases, show a clear visual separation between phase 1/2, phase 3, and phase 4. The cells from phase 2 in the early-/mid secretory mesenchymal cluster (arrows) are from samples 65 and 105.

g, h Heatmaps showing the expression of menstrual cycle phase markers (as in Fig. 2d) of the eutopic endometrial epithelial cells (g) and mesenchymal cells (h) from the Tan et al. 2022 single-cell atlas. C01-C03 are control donors and E01-E09 are endometriosis patients. The menstrual cycle phase of the highest marker gene expression is annotated under phase. Menstrual cycle phase marker genes overlapping with the main marker genes from Wang et al. 2020 Fig. 4a (epithelial) and 4b (mesenchymal) are indicated with an Asterix. The hormonal treatment of each donor is given below the heatmap. Exogenous hormonal treatment with NE/E2 fails to synchronize menstrual cycle phase markers in the eutopic endometrium of donors.

Supplementary Figure 4

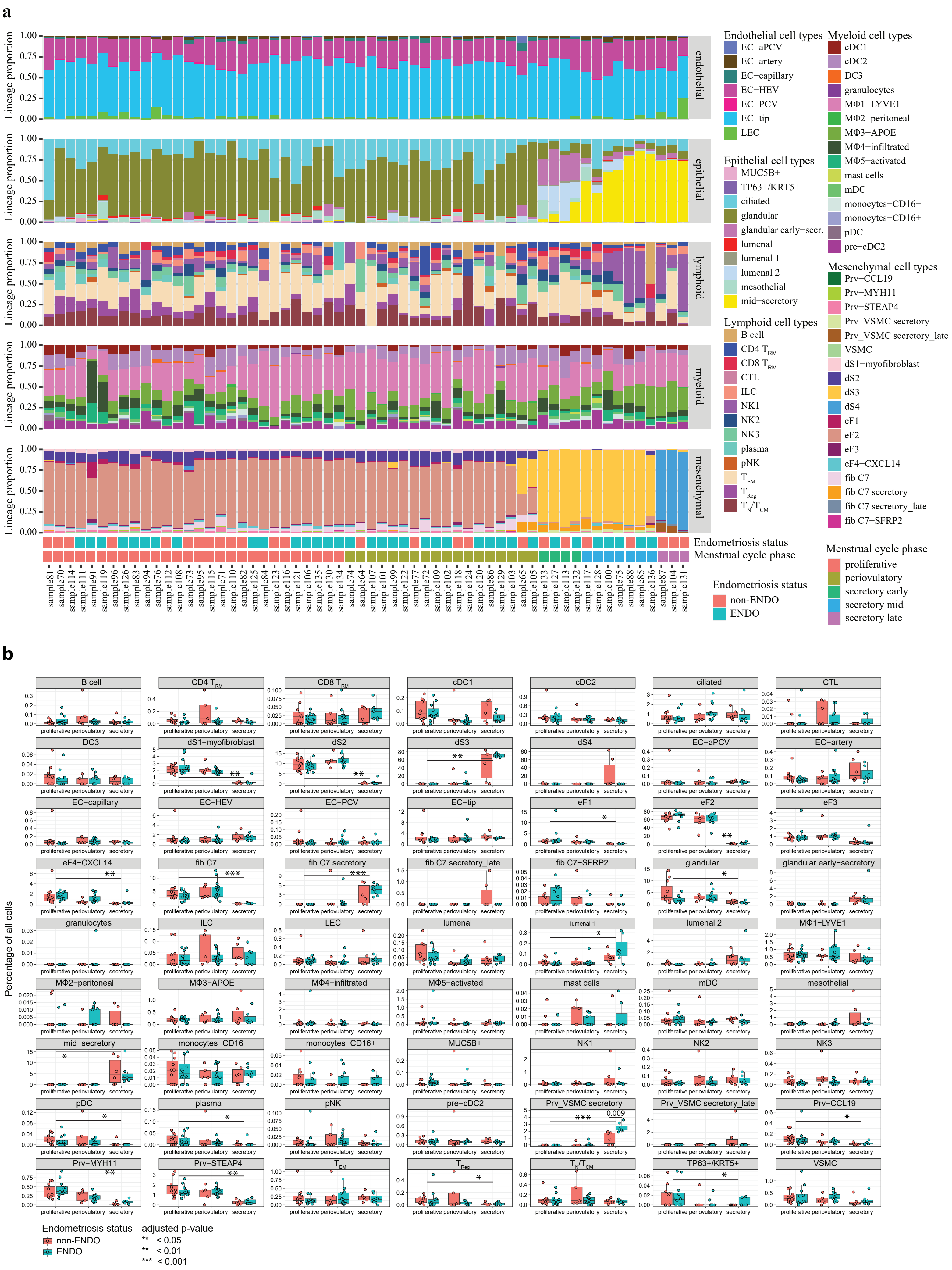

Supplementary Figure 4. **Cell type frequency changes are mainly driven by the menstrual cycle, not by the endometriosis status.**

a Bar plots of cell type frequencies per sample stratified by the main 5 cell types and sorted by menstrual cycle phase and pseudo time. There is no clear trend separating ENDO and non-ENDO.

b Box plots of refined cell type frequencies between the main menstrual cycle phases. 19 of the 63 cell types showed significant frequency change between proliferative (n = 23) and secretory (n = 13) samples. p-values for cell type frequency change between the proliferative and secretory menstrual cycle phase were calculated with a two-tailed Student's t-test and adjusted for multiple testing (Benjamini-Hochberg); * adjusted p-value < 0.05, ** adjusted p-value < 0.01, *** adjusted p-value < 0.001. Box plots show: center line, median; box limits, 25th–75th percentiles (IQR); whiskers, most extreme points within 1.5×IQR; points, individual samples. The only frequency change between ENDO and non-ENDO, stratified by the main menstrual cycle phase and with an unadjusted p-value < 0.05, was in secretory mural cells (Prv_VSMC secretory, unadjusted p-value = 0.009).

Supplementary Figure 5

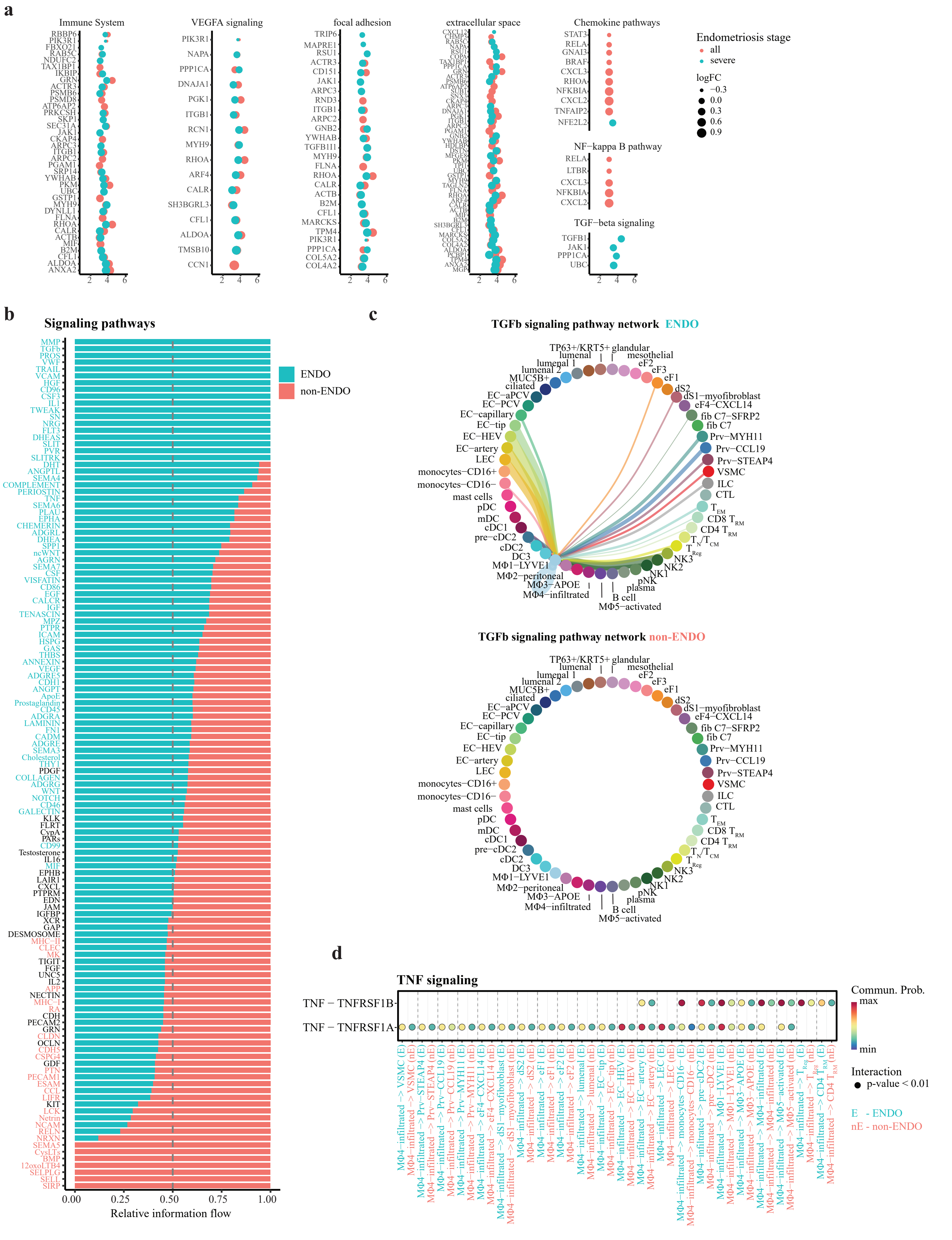

Supplementary Figure 5. **Inflammation, Adhesion, Proliferation, and Angiogenesis pathways and ligand-receptor interactions are upregulated in endometriosis endometrium.**

a Genes underlying selected pathways enriched in the cell-type- and stage-wise functional enrichment analysis (ENDO, n = 12, and non-ENDO, n = 11 samples).

b Signaling pathways with relative information flow from CellChat analysis, ranked by overall information flow within the inferred networks between ENDO (n = 12) and non-ENDO (n = 11). Signaling pathways with a turquoise font have overall enriched activity in ENDO and pathways with a red font have overall enriched activity in non-ENDO.

c Circle plot of TGFb signaling pathway between all cell types in ENDO (top, n = 12 samples) and non-ENDO (bottom, n = 11 samples, no significant interactions).

d Bubble plot of selected communication probabilities within the TNF signaling pathway with significant interaction probabilities (unadjusted p-value < 0.01). Further p-value adjustment is not recommended by the CellChat author. ENDO (n = 12) and non-ENDO (n = 11) samples.

Supplementary Figure 6

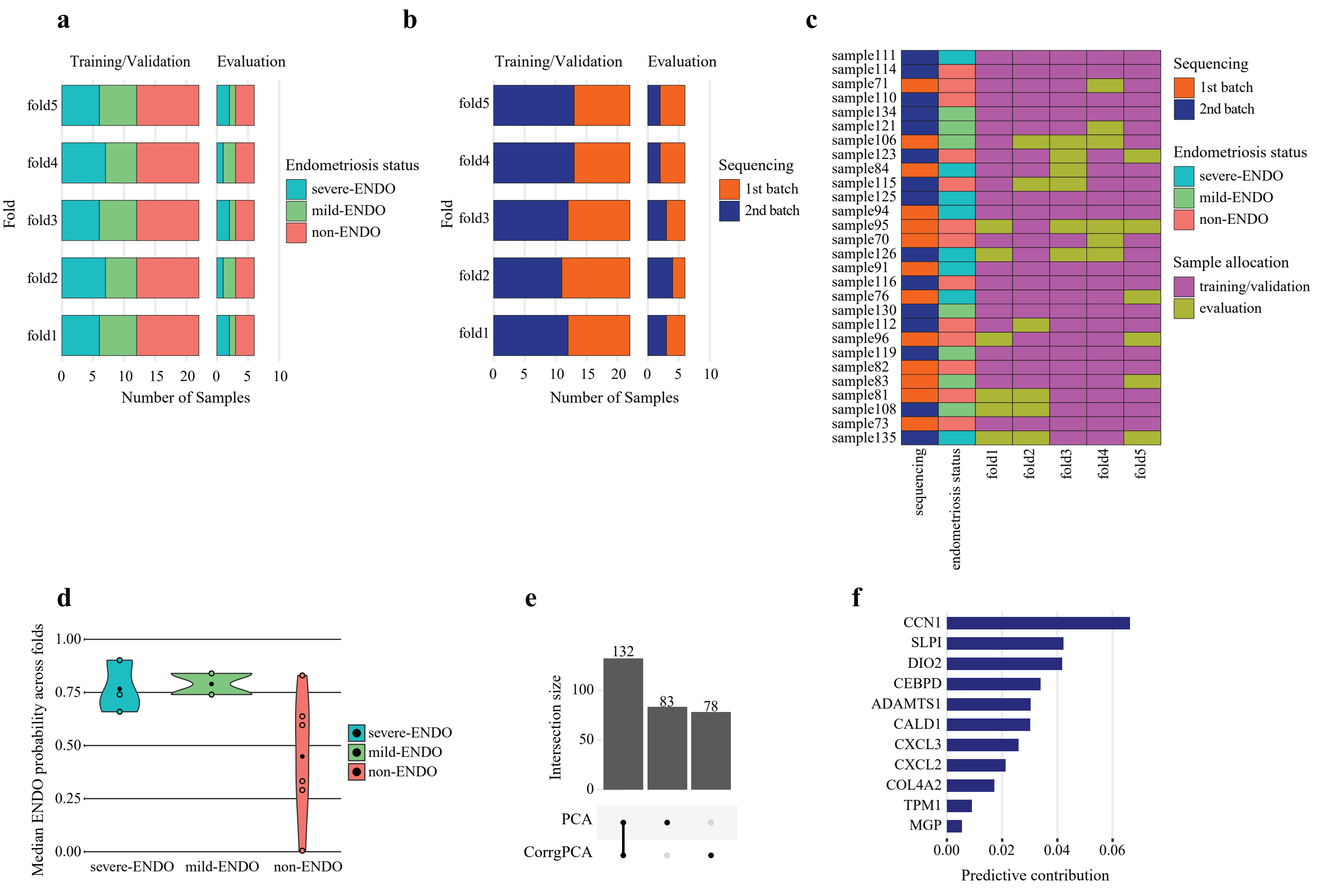

Supplementary Figure 6. **Parameter distributions and predictions of the ScaiVision model training.**

a, b Endometriosis status and sequencing runs are similarly distributed between the training, validation, and evaluation folds.

c Heatmap showing the parameters of each sample, as well as its fold-wise allocation to training/validation and evaluation through the 5-fold Monte Carlo cross-validation scheme.

d Exemplary violin plot of median endometriosis probability (black dot) from the top-ranked learner from the CorrgPCA 3-CV prediction for severe-ENDO, mild-ENDO, and non-ENDO samples, with no discernible difference between mild-ENDO and severe-ENDO prediction.

e UpSet plot of the unified gene signature from PCA (n = 215) and CorrgPCA (n = 210) reveals a substantial 60% gene overlap (n = 132).

f Bar plot illustrates the predictive contribution of each gene from the 11-gene signature. The scores were derived using the Integrated Gradients and deepLIFT method, implemented in the Captum software, and applied to the 11-gene model.

Supplementary Figure 7

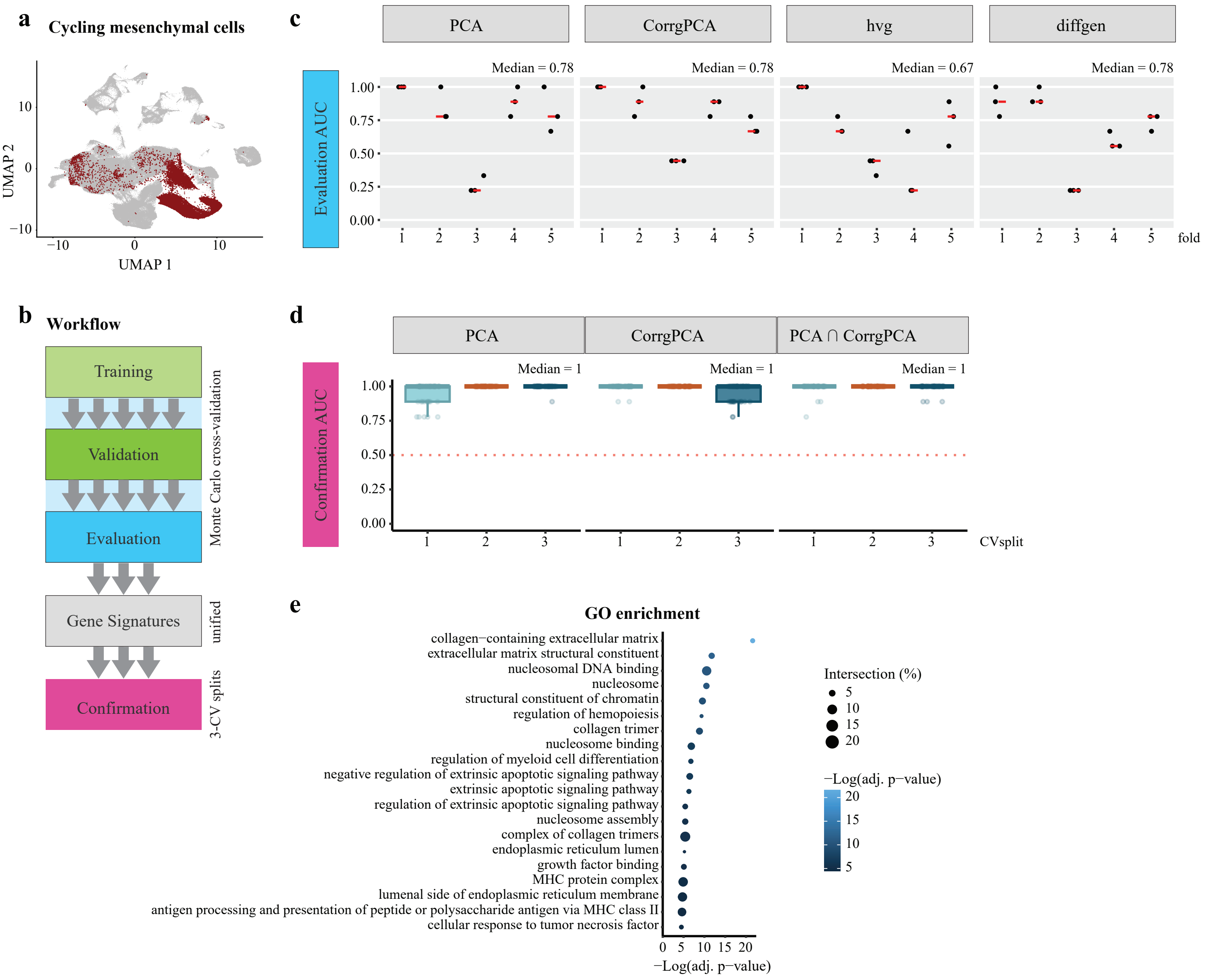

Supplementary Figure 7. **ScaiVision prediction of endometriosis based on cycling mesenchymal cells.**

a UMAP of the endometrium single-cell atlas with cycling mesenchymal cells highlighted (red).

b Workflow outlining the ScaiVision model training and gene signature confirmation process.

c Scatterplots of evaluation AUCs split by feature selection methods (PCA, CorrgPCA, hvg, and diffgen). The median evaluation AUC per fold is represented by the red line. The median AUC per plot is shown on its top right. PCA, CorrgPCA and diffgen performed best in the evaluation with median AUCs of 0.78.

d Boxplot of the confirmation AUC for the unified PCA and CorrgPCA gene signatures and their intersect (PCA ∩ CorrgPCA), showcasing median AUCs of 1 for each CV split. Box plots show: center line, median; box limits, 25th–75th percentiles (IQR); whiskers, most extreme points within 1.5×IQR; points, outliers.

e Top 20 enriched gene ontology (GO) terms and pathways, ranked by the adjusted p-value, highlight structural terms, myeloid cell differentiation, apoptosis, growth factor binding, MHC and responses to TNF. Statistical significance was determined using a one-sided hypergeometric test, with multiple testing correction (g:SCS method, R gprofiler2 package).

Supplementary Figure 8

CXCL3 immunohistochemistry stainings

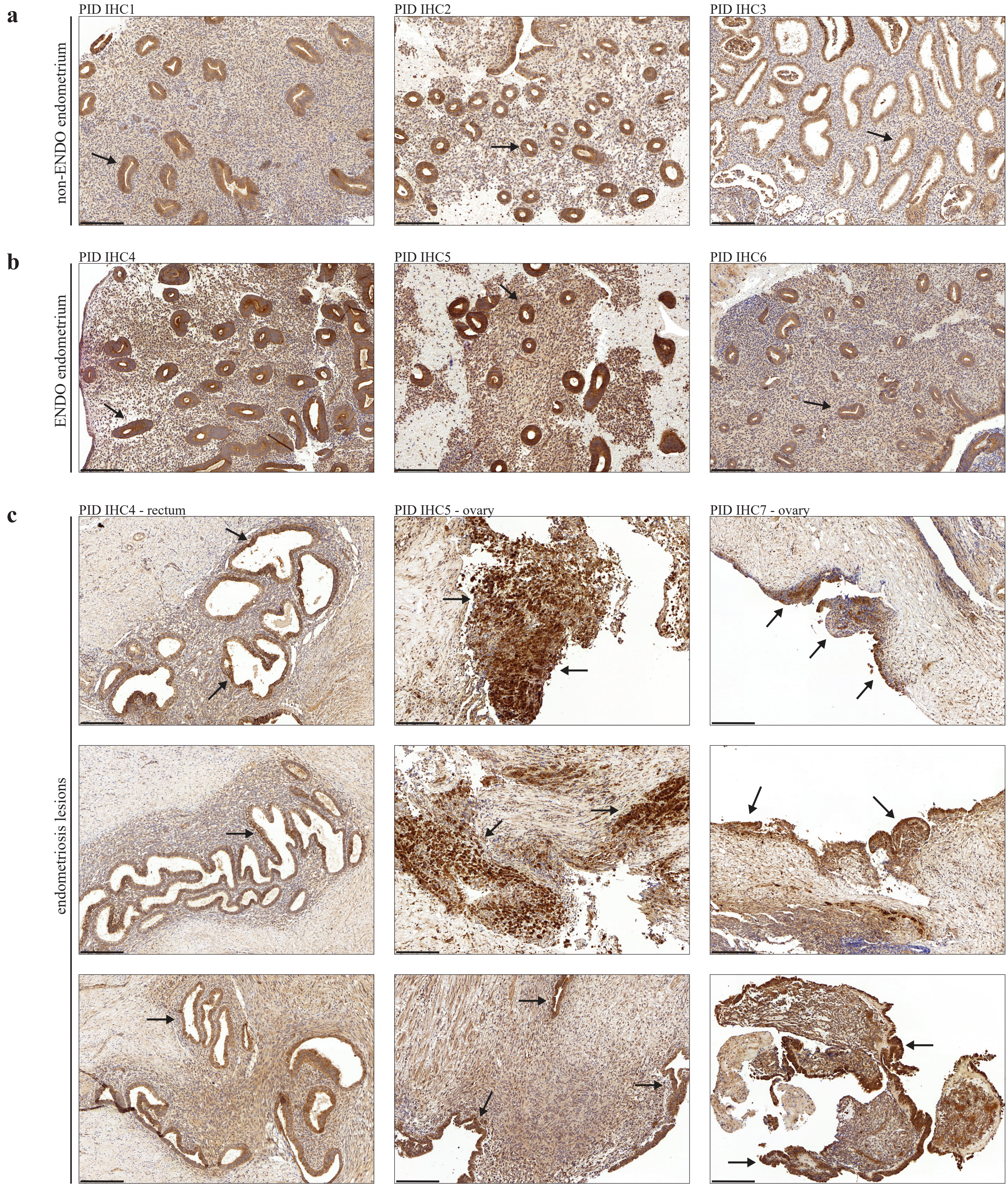

Supplementary Figure 8. **Increased CXCL3 in the endometrium and endometriosis lesions of women with endometriosis.**

CXCL3 immunohistochemistry stainings (scale bar: 0.2 mm) from: (a) eutopic endometrium of non-endometriosis donors (n = 3), (b) eutopic endometrium (n = 3), and (c) endometriosis lesions (n =3) from donors with severe endometriosis. Donor IDs are labeled above each image, with additional donor details provided in Supplementary data 1. In the first two columns, the two donors of the eutopic endometrium images (b) correspond to the lesion images (c). Arrows in (a) and (b) indicate epithelial cells within endometrial glands, showing predominantly darker CXCL3 staining in ENDO samples (b) compared to non-ENDO (a). In the lesion images, arrows highlight epithelial cells with intense CXCL3 expression (c).
