# Supplementary material for: Endometriosis-related alterations in the endometrium revealed by integrated single-cell and AI-powered approaches
Source: Nat Commun. 2026 May 20;17:6688. doi: 10.1038/s41467-026-73020-4 (PMC13385355; doi:10.1038/s41467-026-73020-4)
Supplement: Supplementary file 2 — Description Of Additional Supplementary File [file 41467_2026_73020_MOESM2_ESM.pdf]

Description of additional supplementary items

Supplementary data 1. Clinical data of study participants and sample details.

Supplementary data 2. Menstrual cycle specific marker genes of the main five cell types.

Supplementary data 3. Cell type frequency stratified by sample, endometriosis status, and menstrual cycle stage.

Supplementary data 4. Cell-type- and stage-wise differentially expressed genes of the proliferative menstrual cycle phase samples with stringent exclusion criteria applied. Differential expression analysis was performed using *muscat* as described in the Methods and the *FindMarkers* function, applying the likelihood ratio (LR) test within a logistic regression framework.

Supplementary data 5. Pathways and terms revealed by cell-type- and stage-wise gene enrichment analysis. Statistical significance was determined using a one-sided hypergeometric test, with p-values adjusted for multiple testing using the g:SCS method from the gprofiler2 R package.

Supplementary data 6. ScaiVision performance across learners for all cell model trainings.

Supplementary data 7. ScaiVision PCA and corrgPCA gene signatures for all cell models.

Supplementary data 8. Cell-type-wise percentage of cells within the top 10% of filter scores for ENDO or non-ENDO prediction.

Supplementary data 9. ScaiVision PCA and corrgPCA gene signatures for cycling mesenchymal cell models.

Supplementary data 10. Comparison of this study with published single-cell studies and reported interactions
