# Supplementary material for: Endometriosis-related alterations in the endometrium revealed by integrated single-cell and AI-powered approaches
Source: Nat Commun. 2026 May 20;17:6688. doi: 10.1038/s41467-026-73020-4 (PMC13385355; doi:10.1038/s41467-026-73020-4)
Supplement: Supplementary file 13 — Reporting summary [file 41467_2026_73020_MOESM13_ESM.pdf]

nature portfolio

Corresponding author(s): Lea.Duempelmann@unibe.ch, Michel.Mueller@insel.ch

Last updated by author(s): 2026/03/15

Reporting Summary

Nature Portfolio wishes to improve the reproducibility of the work that we publish. This form provides structure for consistency and transparency in reporting. For further information on Nature Portfolio policies, see our Editorial Policies and the Editorial Policy Checklist.

Statistics

For all statistical analyses, confirm that the following items are present in the figure legend, table legend, main text, or Methods section.

n/a	Confirmed	
☐	☒	The exact sample size (*n*) for each experimental group/condition, given as a discrete number and unit of measurement
☐	☒	A statement on whether measurements were taken from distinct samples or whether the same sample was measured repeatedly
☐	☒	The statistical test(s) used AND whether they are one- or two-sided<br>*Only common tests should be described solely by name; describe more complex techniques in the Methods section.*
☐	☒	A description of all covariates tested
☐	☒	A description of any assumptions or corrections, such as tests of normality and adjustment for multiple comparisons
☐	☒	A full description of the statistical parameters including central tendency (e.g. means) or other basic estimates (e.g. regression coefficient) AND variation (e.g. standard deviation) or associated estimates of uncertainty (e.g. confidence intervals)
☐	☒	For null hypothesis testing, the test statistic (e.g. *F*, *t*, *r*) with confidence intervals, effect sizes, degrees of freedom and *P* value noted<br>*Give P values as exact values whenever suitable.*
☒	☐	For Bayesian analysis, information on the choice of priors and Markov chain Monte Carlo settings
☐	☒	For hierarchical and complex designs, identification of the appropriate level for tests and full reporting of outcomes
☐	☒	Estimates of effect sizes (e.g. Cohen's *d*, Pearson's *r*), indicating how they were calculated

Our web collection on statistics for biologists contains articles on many of the points above.

Software and code

Policy information about availability of computer code

Data collection	Single-cell capture, barcoding and cDNA library generation were performed with the 10x Genomics Chromium Single Cell Gene Expression workflow (Chromium NextGEM Single Cell 5' Library and Gel Bead Kit v1.1, Chromium Controller) according to the manufacturer’s protocol (CG000207_ChromiumNextGEMSingleCellV_D_J_ReagentKits_v1.1_UG_RevE). 25,000 viable cells per sample were captured in a single reaction. cDNA libraries were sequenced with an iSeq 100 Sequencing System (Illumina, 2 x 150 cycles) for quality check and balancing of samples, based on the iSeq run calculated Loading Factor for per Cell Normalisation. Deep sequencing was performed with a NovaSeq 6000 System (Illumina, S4, 2 x 100 cycles).
Data analysis	The NovaSeq runs were demultiplexed using the bcl2fastq v2.20.0.422 software from Illumina.<br>Gene indexing, cell debarcoding, deduplication, read mapping, and estimation of transcript-level expression by pseudo-alignment were performed with the Salmon software package AlevinQC (1.6.0). The Seurat (4.1.0), scDblFinder (1.4.0) and scater (1.18.0) packages, implemented in snakemake (5.14), were used to perform quality control, integration of the endometrial single-cell atlas and visualization of the data on the sample-, cell- and gene-level.<br>Following data analysis were performed, with the R package names and versions given in brackets: Sample-wise Principal Component Analysis (PCAtools_2.10.0), annotation transfer (symphony_0.1.1), identification of menstrual cycle phase-specific markers (Seurat_5.0.3, FindAllMarkers), time trajectory analysis (Monocle3_1.3.4), cluster-wise differential expression analysis (bioconductor-muscat_1.12.0) and ligand-receptor analysis (CellChat_2.0.0).<br>Endometriosis predictions were performed with the interpretable neural network algorithm CellCnn (version 0.2), implemented in the ScaiVision platform (version 1.6.3) using PyTorch (torch_1.10.2)<br>Custom code for atlas integration, Sample-wise Principal Component Analysis, annotation transfer, time trajectory analysis, cluster-wise differential expression analysis, and ligand-receptor analysis are available at https://github.com/DuempelmannLea/

endometriosis_endometrium_scRNA_atlas. All raw sequencing data and the processed Seurat object are available at NCBI's Gene Expression Omnibus (series accession number: GSE266265; https://www.ncbi.nlm.nih.gov/geo/query/acc.cgi?&acc=GSE266265).

For manuscripts utilizing custom algorithms or software that are central to the research but not yet described in published literature, software must be made available to editors and reviewers. We strongly encourage code deposition in a community repository (e.g. GitHub). See the Nature Portfolio guidelines for submitting code & software for further information.

Data

Policy information about availability of data

All manuscripts must include a data availability statement. This statement should provide the following information, where applicable:

- Accession codes, unique identifiers, or web links for publicly available datasets
- A description of any restrictions on data availability
- For clinical datasets or third party data, please ensure that the statement adheres to our policy

The data supporting the findings from this study are available within the manuscript and its supplementary information. All raw sequencing data and the processed Seurat object are available at NCBI's Gene Expression Omnibus (series accession number: GSE266265; https://www.ncbi.nlm.nih.gov/geo/query/acc.cgi?&acc=GSE266265). Source data are provided with this paper.

Research involving human participants, their data, or biological material

Policy information about studies with human participants or human data. See also policy information about sex, gender (identity/presentation), and sexual orientation and race, ethnicity and racism.

Reporting on sex and gender	Given that endometriosis almost exclusively affects individuals with a uterus, our research design inherently revolves around cisgender women and other people assigned female at birth. No information was collected on gender. No sex-based or gender-based analysis was performed. Before undergoing laparoscopy, each participant was assigned a unique identifier to ensure anonymity, with no possibility of tracing it back to their individual identity. Consent for the publication of de-identified results was obtained from each study participant at their enrolment in the study.
Reporting on race, ethnicity, or other socially relevant groupings	Information on race, ethnicity, or other socially relevant groupings was not systematically recorded for patients in this dataset.
Population characteristics	All covariate-relevant characteristics from our study participants, such as age, endometriosis status and type, adenomyosis diagnosis, and pain scores are provided in Table 1.
Recruitment	Women scheduled for laparoscopic surgery at Frauenklinik Bern were recruited for our study. Inclusion criteria for this study include women who provide Informed Consent, were above 18 years old and scheduled for laparoscopic surgery for reasons including symptoms of endometriosis, tubal ligation, salpingectomy, hysterectomy, idiopathic infertility, pelvic pain or other gynaecological pathologies as part of their planned clinical treatment. Patients with pre-existing inflammatory diseases, malignancy, pregnancy or lactating. The final diagnosis of endometriosis status was determined by pathologists and clinicians after the inclusion of patients into the study. Patients participated without compensation.
Ethics oversight	The study at Frauenklinik Bern was approved by the Swiss Ethic Committee (KEK-BE 01780, 2019).

Note that full information on the approval of the study protocol must also be provided in the manuscript.

Field-specific reporting

Please select the one below that is the best fit for your research. If you are not sure, read the appropriate sections before making your selection.

☒ Life sciences ☐ Behavioural & social sciences ☐ Ecological, evolutionary & environmental sciences

For a reference copy of the document with all sections, see nature.com/documents/nr-reporting-summary-flat.pdf

Life sciences study design

All studies must disclose on these points even when the disclosure is negative.

Sample size	No formal sample-size calculation was performed. Our endometrial single-cell atlas includes samples from 35 women with endometriosis and 25 without. Of our 330 study participants with cryopreserved endometrial biopsies, we included most fulfilling our stringent inclusion/exclusion criteria, comprising no external hormone intake of participants the 3 months before laparoscopy and cell viability > 70% before single-cell capture. Additional selection criteria were a balance between endometriosis status and menstrual cycle phases.
Data exclusions	All exclusion criteria were pre-established.<br>Patients without endometrial biopsy, serum or reported menstrual cycle day were excluded. Patients with hormonal treatment or IUD less than 3 months before the operation were excluded. Patients with no confirmation of endometriosis by histology, or patients diagnosed to be endometriosis-free but have adenomyosis were excluded. Patients with inflammatory diseases, malignancies, lactating or the onset of the last period more than 35 days before the surgery were excluded.<br>For single-cell capture, samples with viability under 70% and less than 100,000 viable cells were excluded. After iSeq sequencing analysis,

	samples without a steep drop-off in their barcode rank plot were excluded, due to its indication of poor separation between the cell-associated partitions and the background partitions. During single-cell analysis, cells were excluded with less than 200 or more than 3500 detected genes, more than 25% mitochondrial genes or identified as doublets using scDblFinder. Furthermore, genes that were expressed in less than 4 cells per sample were excluded.<br>For cell type frequency, differential gene expression and ligand-receptor analysis stringent patient exclusion criteria were applied (Table 1), due to a potential influence of these clinical parameters. For ScaiVision analysis, samples with less than 5000 cells were excluded to ensure robust model training and prediction.
Replication	Retrospective alignment of our atlas samples to a comprehensive C1 menstrual cycle atlas from healthy volunteers with a regular cycle (Wang et al., Nature Medicine, 2020), resulted in perfect correspondence of menstrual cycle phase allocation. To compare cell type frequencies across menstrual phases, we utilized 23 samples from the proliferative phase, 18 from the periovulatory phase, and 13 from the secretory phase, adhering to stringent clinical exclusion criteria. For differential gene expression and ligand-receptor analysis, the 12 samples from women with endometriosis and 11 without endometriosis from the transcriptionally homogeneous proliferative menstrual cycle phase were used, with stringent clinical exclusion criteria applied, ensuring reliable results.
Randomization	For menstrual cycle phase analysis, participant samples were allocated into experimental groups based on their menstrual cycle phase (proliferative, periovulatory, and early, mid, or late secretory). For differential gene expression and ligand-receptor analysis, participant samples from the proliferative menstrual cycle phase were allocated into experimental groups based on their endometriosis status (no endometriosis and mild or severe endometriosis).<br>For ScaiVision network training each sample was assigned labels corresponding to the endometriosis status (no, mild or severe endometriosis) and sequencing batch. To ensure the preservation of relative proportions of technical batches and the non-ENDO/ENDO endpoints, sample allocation was conducted using a nested 5-fold Monte Carlo cross-validation scheme. A detailed description of this process is provided in the methods section.
Blinding	The primary aim of our study was to compare women with and without endometriosis. Therefore, blinding was not considered relevant to our study.

Reporting for specific materials, systems and methods

We require information from authors about some types of materials, experimental systems and methods used in many studies. Here, indicate whether each material, system or method listed is relevant to your study. If you are not sure if a list item applies to your research, read the appropriate section before selecting a response.

Materials & experimental systems

n/a	Involved in the study
☐	☒ Antibodies
☒	☐ Eukaryotic cell lines
☒	☐ Palaeontology and archaeology
☒	☐ Animals and other organisms
☒	☐ Clinical data
☒	☐ Dual use research of concern
☒	☐ Plants

Methods

n/a	Involved in the study
☒	☐ ChIP-seq
☒	☐ Flow cytometry
☒	☐ MRI-based neuroimaging

Antibodies

Antibodies used	CXCL3 antibody from biorbyt, Catalog Number: orb13448, species/host: Rabbit, Clonality: polyclonal, Immunogen: KLH conjugated synthetic peptide derived from human CXCL3 (35-107/107 aa)
Validation	Relevant citation: https://pmc.ncbi.nlm.nih.gov/articles/PMC11017417/

Plants

Seed stocks	*Report on the source of all seed stocks or other plant material used. If applicable, state the seed stock centre and catalogue number. If plant specimens were collected from the field, describe the collection location, date and sampling procedures.*
Novel plant genotypes	*Describe the methods by which all novel plant genotypes were produced. This includes those generated by transgenic approaches, gene editing, chemical/radiation-based mutagenesis and hybridization. For transgenic lines, describe the transformation method, the number of independent lines analyzed and the generation upon which experiments were performed. For gene-edited lines, describe the editor used, the endogenous sequence targeted for editing, the targeting guide RNA sequence (if applicable) and how the editor was applied.*
Authentication	*Describe any authentication procedures for each seed stock used or novel genotype generated. Describe any experiments used to assess the effect of a mutation and, where applicable, how potential secondary effects (e.g. second site T-DNA insertions, mosiacism, off-target gene editing) were examined.*
