# Supplementary material for: Endometriosis-related alterations in the endometrium revealed by integrated single-cell and AI-powered approaches
Source: Nat Commun. 2026 May 20;17:6688. doi: 10.1038/s41467-026-73020-4 (PMC13385355; doi:10.1038/s41467-026-73020-4)
Supplement: Supplementary file 14 — Transparent Peer Review file [file 41467_2026_73020_MOESM14_ESM.pdf]

nature portfolio

Peer Review File

Endometriosis-Related Alterations in the Endometrium revealed by Integrated Single-Cell and AI-Powered Approaches

Corresponding Author: Dr Lea Duempelmann

This manuscript has been previously reviewed at another journal. This document only contains information relating to versions considered at Nature Communications.

This file contains all reviewer reports in order by version, followed by all author rebuttals in order by version.

Version 1:

Reviewer comments:

Reviewer #1

(Remarks to the Author)
The authors have adequately addressed all my comments and the manuscript has improved as a result. In particular, the Discussion is more focused and powerful.

(Remarks on code availability)

Reviewer #3

(Remarks to the Author)

1. Two reviewers have flagged other published work that has shown variation in cell composition and single-cell analysis of endo samples. It's argued that the novelty here is that this work "the first to comprehensively study endometriosis-specific changes within the eutopic endometrium stratified by the donor's menstrual cycle phase". However, as flagged by another reviewer, there are substantial gaps in samples across relevant tissues that are highly relevant across certain stages of the cycle and predominantly focus on a single stage (proliferative). I appreciate there may be some practical reasons for not including these tissue samples and missing stages, but it undermines the point of novelty.
2. The finding of nerve fiber growth enrichment in endometriosis is noteworthy. It would be beneficial to explore the specific pathways or genes involved in this mechanism. The revision noted additional details have been added (lines 260-262). However, this consists of mentioning one of the differentially expressed genes.
3. A critical comment raised by multiple reviewers is the necessity of out-of-sample validation for the prediction model. The argument given is that this is out of scope for this work, which instead focuses on "the single-cell analysis and pathophysiology of endometriosis". However, the title, abstract, introduction, results and discussion all highlight diagnosis and prediction as points of novelty "we are the first to develop an endometrial biopsy-based diagnosis method". Given this, I strongly encourage the authors to consider developing this work, as a small N within the sample and small K cross-validation are not statistically robust enough to estimate AUC accurately. Coupled with variation in patient samples of course.
4. The sample variance of AUC across folds is high. Performing more folds will help accurately estimate this variance.
5. Reviewers suggested looking at alternative DEG and integrating DEG methods to account for method bias. This is a well-established issue in the field and likely leads to biased results.
6. Can you extend beyond cell types to identify disease-associated cell states, not just cell types?
7. All reviewers suggested validation of some biological results. Given the well-established biases of the analysis methods, this remains something that would be incredibly valuable.
8. Reviewer #3 asked about significance thresholds and if multiple testing correction has been performed. The rebuttal pointed to the reply to Reviewer #1 comment 11d. This comment relates to the practicality of a gene signature from

proliferative stage endometrium for a diagnostic test. It’s not clear what has been changed, or indeed if multiple testing correction was performed.

(Remarks on code availability)
Code is available for some areas of analysis. Data paths are hard coded and not not commented, so reproducing analysis would be challenging

Reviewer #4

(Remarks to the Author)
The author's response has made a commendable effort to articulate the uniqueness of their study and to compare it in detail with previous research. However, there are still some issues:

1.Previous review comments mentioned that the pathophysiological mechanisms discussed in the study are not novel. While the author's response lists their unique dataset and methods, it does not sufficiently emphasize how these methods bring new insights into the pathophysiological mechanisms. Moreover, the previously mentioned review comments noted that the biological pathways and molecular mechanisms have been extensively reported in studies on endometriosis. The authors' response should specifically point out what new regulatory mechanisms or modes of action their study has discovered in these known pathways, particularly highlighting how their single-cell data reveal the cell type specificity of these processes.

2.Your response briefly mentions the SDF-1α/Rho/mDia pathway influenced by CXCL12. However, it lacks a detailed discussion on how this pathway specifically contributes to the pathological nerve growth observed in endometriosis. If possible, please include new data or specific results from the study that show the differential expression of CXCL12 and its impact on nerve growth. If the study includes pathway analysis, highlighting how these pathways are enriched in endometriosis samples compared to controls would be valuable.

3.The reviewer suggested the use of spatial proteomics or spatial transcriptomics techniques to validate the interactions between cells. The author mentioned using spatial immunohistochemistry to validate the expression of CXCL3. Although this is a good start, the author did not specify how this method compares with other spatial techniques recommended by the reviewer, nor whether other spatial data analysis techniques were considered or planned for use in the study. Additionally, the author mentioned cross-validation methods in their response, including differential gene expression (DEG) analysis, ligand-receptor analysis, and AI analysis. However, they did not provide detailed descriptions of how these methods were specifically applied to their dataset, nor how these analyses support their conclusions interactively.

4.The author mentions specificity and sensitivity achieved with bulk sequencing data but does not detail the specific validation techniques used for the machine learning model developed from single-cell data, which is the focus of the paper. Moreover, the author does not discuss how the model performs under different conditions or with different datasets. Information on how the model handles variability in data, which is crucial for demonstrating robustness and generalizability, is lacking. In addition, the reviewer suggested using external validation with independent datasets as a way to strengthen the reliability and applicability of the predictive model. The author's response does not address whether such external validation has been or will be conducted.

(Remarks on code availability)
After examining the code, I realised that reproducing the results in the paper was impossible. The github links lacked an introduction to the data as well as a download link, and there was no introduction to the code, such as what each code was used for, or to reproduce which figure from the paper. The author's github profile is blank, which is not conducive to reproducing the results of the paper.

Version 3:

Reviewer comments:

Reviewer #3

(Remarks to the Author)

(Remarks on code availability)
Code was avaliable but I was unable to run it due to hardcoding of file paths.

Reviewer #4

(Remarks to the Author)
In their response, the authors highlight the study's new insights into the pathophysiology of endometriosis through cell type-specific single-cell analysis and describe improvements in code and GitHub data availability. However, the authors seem

intent on not addressing these issues and there are a number of questions that must be clarified or added to the evidence.

Q1:The authors list new cell-type-specific interactions within known pathways (e.g., TNF, VEGF, TGFβ), yet the response lacks a direct, side-by-side comparison showing exactly what is novel relative to published work.

Q2. The brief mention of the CXCL12-driven SDF-1α/Rho/mDia pathway and its role in aberrant nerve growth lacks direct data support (e.g., differential expression, pathway enrichment). Please provide pathway-level analyses (e.g., GSEA) or gene-expression data demonstrating significant up-regulation in endometriosis samples. Moreover, if this pathway is important, it is still recommended to illustrate the significance of CXCL12 or SDF-1α/Rho/mDia in ENDO patient tissues at least in Supplementary with differential expression results or pathway enrichment maps.

Q3. If it is difficult to carry out further spatial genomics experiments, it is recommended that the methodology be described at least in more detailed length. How was the ligand-receptor analysis done, For example with CellChat/CellPhoneDB/other tools? In which datasets were these analyses implemented respectively? How do the results corroborate with DEG analyses?

(Remarks on code availability)
It is a nice code. We can install and run the code.

Version 4:

Reviewer comments:

Reviewer #4

(Remarks to the Author)
The authors have addressed all my comments.

(Remarks on code availability)
Nice job.

Open Access This Peer Review File is licensed under a Creative Commons Attribution 4.0 International License, which permits use, sharing, adaptation, distribution and reproduction in any medium or format, as long as you give appropriate credit to the original author(s) and the source, provide a link to the Creative Commons license, and indicate if changes were made.
In cases where reviewers are anonymous, credit should be given to 'Anonymous Referee' and the source.
The images or other third party material in this Peer Review File are included in the article’s Creative Commons license, unless indicated otherwise in a credit line to the material. If material is not included in the article’s Creative Commons license and your intended use is not permitted by statutory regulation or exceeds the permitted use, you will need to obtain permission directly from the copyright holder.
To view a copy of this license, visit https://creativecommons.org/licenses/by/4.0/

Point-by-point Response to Reviewer's Comments

We thank the reviewers for their excellent, extensive, detailed, and helpful feedback on our manuscript. Your insights not only helped improve our manuscript but also provided stimulating input for our research and inspired interesting discussions. We deeply appreciate your time and effort in reviewing our work and are grateful for your valuable contributions. Below, we provide a detailed response to each comment.

Of note, page and line numbers in our responses correspond to the re-submitted manuscript with 'Simple Markup'.

Reviewer #1:
Remarks to the Author:
This study reports the largest single cell atlas for human endometrial tissue to date (>460,000 cells) derived from eutopic endometrium of a large number of women with and without endometriosis. Importantly, the samples were obtained from women not taking hormones and came with comprehensive patient phenotypic data including pelvic pain scores, circulating hormone concentrations and careful dating of the menstrual cycle stage, which is an important improvement in comparison to other published endometrial atlases. The authors focus on proliferative stage endometrium and show significant differences in DEGs, ligand-receptor interactions associated with increased inflammation, adhesion, proliferation, cell survival and angiogenesis between endometriosis and controls, mainly in the stromal/vascular cell types. The authors use AI to predict endometriosis of varying severity generating an 11 gene-based signature, with potential for use in diagnostics. However, how such a diagnostic test would be non-invasive to obtain endometrial samples and possibly isolation of specific cell types is not clear. This would be difficult to apply to menstruating girls with severe pelvic pain.
Deep exploration of human endometrium, a highly heterogeneous tissue, constantly changing as the menstrual cycle progresses, which sheds and then regenerates from a remaining germinal layer, is in its infancy. This study finds an area previously not pursued in detail in earlier single cell atlases on endometrium and/or endometriosis (refs 16, 17, 23, 58), therefore adds to important and new information in a generally neglected tissue. It could have significant impact on endometriosis diagnosis, which currently has an unacceptable 7-10 year diagnostic delay due to lack of a non-invasive diagnostic test that picks up all forms of the disease. It will likely be informative to other highly regenerative mucosal tissues in the body such as the gastrointestinal tract.

We thank Reviewer 1 for this comprehensive summary and for pointing out the importance of exclusion of exogenous hormone intake, careful dating of the menstrual cycle, detail of our study and significant impact on endometriosis diagnosis and beyond.

Novelty lies in the in depth analysis of proliferative stage endometrial mesenchymal cells to overcome heterogeneity of rapidly changing endometrial cellular gene expression profiles.
However, were mesenchymal rather than epithelial cells chosen as they comprised the majority of the cells? There appeared to be a disproportionately low percentage of epithelial cells in these samples.
We did not pre-select cell types for single-cell capture, therefore the high percentage of stromal cells reflects the disproportionately high amount of this cell type in the endometrium. This can also be seen in Garcia-Alonso et al. (2021), Fonseca et al. (2023) and Mareckova et al. (2024). As pointed out by the reviewer, a strength of this study is the size and well-characterized patient samples, suggesting that the appropriate ratio between cell types could be reconsidered based on this study.

The strengths of this manuscript lie in the approach used in obtaining high quality of the samples and 1) the associated patient phenotyping, the preciseness of the menstrual cycle stage determination using multiple methods, 2) the large number of samples utilized and the identical processing protocols for all samples, 3) the use of AI in finding a small set of signature genes that could be used for a diagnostic test. The data are of high quality and generally clearly presented.
Weaknesses include sampling only the functionalis layer of the endometrium via biopsy (same for almost all other single cell atlas studies in human endometrium), which do not use hysterectomy tissue to include the basalis layer which has a critical role in regenerating the endometrium each month and likely involved in endometriosis pathogenesis. This layer contains the basalis epithelial cells, which generates the new luminal epithelium into which an embryo implants, and epithelial progenitors which may be responsible for regenerating the vertical glands of the functionalis layer each menstrual cycle (ref 64).
We agree that it would be an advantage to also have had the basalis layer from hysterectomies. However, of our patients with our strict exclusion criteria, only very few had hysterectomies, making a statistically valid analysis on rare cell types, such as the stem and progenitor cells, not feasible. An accurate examination of these rare epithelial progenitors and stem cells requires dedicated enrichment from endometrial hysterectomy samples in future work.

Issues needing further clarification and/or experimental work:

1. The experimental design has both strengths and weaknesses. One major strength was that none of the sample donors had been taking hormones (eg contraceptive pills, progestins to manage symptoms), which has been a drawback of other endometrium scRNAseq studies. All samples had been processed by the authors and therefore subjected to similar protocols.
We agree with the reviewer that the exclusion of donors under hormonal treatment and uniform processing of all samples is a major strength of our study and is an important step forward compared to the current knowledge and thank the reviewer for acknowledging this.

a. Despite the large sample number and their well characterised cycle stage, none are from the menstrual stage, even though it is from this stage that the pelvic cavity is seeded with endometrial cells capable of establishing endometriosis lesions. The authors chose to study the proliferative stage in detail as it is less variable and did not study the secretory stages in detail, even though endometrial fragments present in menstrual fluid that reaches the pelvic cavity would be similar to late secretory stage.
At our hospital, patients are Not operated during the menstrual cycle phase due to a higher risk of endometriosis recurrence. The endometrium changes almost daily during the secretory phase (Noyes 1975), therefore many more samples would be required for a well-defined dissection of transcriptional changes between endometriosis and control patients in the secretory stage without getting false positive transcriptional changes caused by menstrual cycle phase change. As the reviewer pointed out earlier, we have the most comprehensive single-cell endometrium atlas to date.

2. In the Methods, please define the progesterone levels for the 3 cycle stages used in this study and how the transcriptional profile was used to determine cycle stage (ie which genes?)
We thank the reviewer for this comment and added the paragraph 'Menstrual cycle phase evaluation' to the Methods section (pages 30-31, lines 674-678).

3. In the Data generated, could the lack of differences in the transcriptional landscape between endometriosis and normal controls in the PCA analysis (up to 10 rounds) indicate a bias in cell types recovered from tissue processing – ie the low epithelial cells at just 5.6% and the preponderance of mesenchymal cells at 88.3%? And the high level of periovulatory and lack menstrual samples?
This is a very good point. We now added to the Extended Data Fig. 1f PCA eigencorplots from PCA analysis on the 5 main cell types separately. After correction for multiple-testing, endometriosis grade indeed correlates with PC3 in myeloid, but not in the first 10 PCs of any other main cell type. We would expect that with a more even sample balance

between proliferative/periovulatory and secretory phase, the menstrual cycle components would dominate the PCs even more.
This great question sparked an internal discussion, and we decided to replace in the manuscript the initial PCA analysis of the integrated assay with the more standard PCA analysis of the SCT assay (Figures 1e, 2a and Extended Data Figures 1e, 1f, 3a).

4. The increase in mural Prv-VSMC in the secretory phase in endometriosis indicates more of these cells will also be shed in menstrual fluid and could assist with vascularization of new lesions establishing in the pelvic cavity. Marker expression for this cell type, ie MCAM suggests these are perivascular endometrial MSC that are shed in menstrual fluid (Masuda et al 2021, PMID: 34011465).
Thanks to the reviewer for pointing out this important literature, which we now added to the manuscript (page 8, lines 152-154).

5. Page 7 line 5-6 – transcriptional changes in ENDO endometrium associated with endometriosis susceptibility. How was this determined? Please include methodology in the Methods section.
We improved the text accordingly (page 8, lines 156-158).

6. Page 7 last 2 lines and first paragraph on p8 are unclear. – the authors liken the role of bone marrow mesenchymal cells upregulated in CXCL2 in maintaining the haemopoietic stem cell niche to immature endometrial cells and endometriosis susceptibility. Please rewrite.
We improved the text accordingly (page 10, lines 203-207).
Mareckova et al. (bioRxiv, 2023) suggest a potential role of CXCL12/CXCR4 axis in endometrial stem/progenitor cells in the basalis layer, however without investigating expression changes in the context of endometriosis (they did not report differential expression of CXCL12/CXCR4 in the endometrium between control and endometriosis patients). We now also cited Mareckova (bioRxiv, 2023) in that paragraph.

The text describes enriched the GO terms (adhesion, migration and invasion) and lists a set of genes, but these are not detailed in the referred to Supplementary Table 5 (RAW data). It is currently not possible to follow this table and the manuscript would be improved if it could be summarized into a new Suppl Table to include the enriched terms in more detail and the genes that make up those GO terms for the different ENDO groups.
Thanks for this comment, we made the table more intuitive and clearer.
In Supplementary Table 5 the enriched terms are given in the column J ‘term_name’ and the genes making up the GO terms are given in column N ‘intersection’ with the different ENDO groups given in column Q ‘endometriosis stage’. To improve the intuitiveness of the table we changed the column name ‘intersection’ to ‘gene intersection between DEGs

and terms' and in the 'endometriosis stage' column all/mild/severe to all-ENDO/mild-ENDO/severe-ENDO. We noticed a duplication of rows due to the row names of the original output table in R and filtered out the duplicate rows.

7. Manuscript organisation – Grouping the description of the data into a single large headings on the basis if bioinformatic tools used covering several topics such as Inflammation, Adhesion, Proliferation and Angiogenesis Pathways would be simplified if broken down into several smaller sections such as Inflammation and Adhesion, Proliferation and Cell Survival, and Angiogenesis. The figures need not change, but smaller focussed sections in the Results on each of these 3 aspects that cover the GO terms and genes involved and include the cell-cell interactions, making the information more accessible to the reader. They could include another heading on the role of WNT and NOTCH in endometrial regeneration and expand on it a bit more.
Thanks for this valuable comment. We re-organized the manuscript accordingly (pages 8-13, lines 155-269)

8. It is of interest that the authors found EC-HEV as a cell type which are usually found in lymph nodes and tertiary lymphoid nodules and are involved in lymphocyte trafficking and inflammation. Human endometrium has lymphoid patches but they are usually in the basalis layer – maybe some of this layer was sampled?
This is a very good observation. We consider the sampling of the basalis layer unlikely with our method of biopsy (endometrial pipelle de Cornier) and believe that these cells might have rather traversed from the basalis layer into the functionalis. These cells have previously been detected and described in the endometrium from endometrial biopsies taken during hysteroscopy by Tan et al. 2022 (DOI: 10.1038/s41556-022-00961-5). Here is a summary table for the Tan et al. single-cell atlas of endothelial cell counts by cell subtype (incl. EC-HEV) and origin (Ctrl = endometrium from control patients, EuE = endometrium from endometriosis patients).

	Ctrl	EuE	EcP	EcPA	EcO
LEC	48	180	103	34	17
EC-artery	32	50	307	251	274
EC-tip	446	608	219	144	770
EC-capillary	92	59	89	89	458
EC-HEV	372	413	126	109	749
EC-PCV	57	35	454	739	70
EC-aPCV	28	22	1381	1802	124

9. Page 11 2nd last line – what do the author mean by "likely replicate across independent menstrual cycles". They have assessed many different patient samples to generate their data.

Thank you for this comment on clarity. We meant between independent menstrual cycle phases of the same individual. We removed this sentence part to avoid confusion among the readers (page 9, lines 177-179).

10. Endometriosis prediction based on AI analysis describing Ext Fig 7 – The identified GO terms (ext Fig 7e rather than fig 7c) suggest the authors should also include collagen and ECM (ie fibrosis potential) and negative regulation of apoptosis (cell survival).
We updated the text accordingly, thanks (pages 14, lines 308-309).

11. Discussion – Overall the final concluding paragraphs do provide a good summary of the experimental data generated in this study and the diagnostic model they describe.
a. There is a long introduction to the Discussion – the information is important and some may belong in the Introduction itself. Otherwise it could be incorporated as the authors summarise and discuss their results. Overall, the Discussion is rather superficial in that it has not compared their studies to other single cell atlases of human endometrium– there are some overlaps as the Tan et al paper also shows upregulation of inflammation, adhesion, proliferation and angiogenesis genes. Alternative explanations for some data has not been included.
We thank the reviewer for this suggestion and strongly revised the discussion (pages 17-19, lines 352-418).
It is correct that there are overlaps in the conclusion with the Tan et al paper. However, we are convinced that these findings are mainly caused by the diverse hormonal effects and lack of sufficient patient numbers. Please let us elucidate.
Tan et al compare the endometrium transcriptome of 3 control patients (2 under hormonal treatment and one not disclosed) to 9 endometriosis patients (under different hormonal treatments). Hormones have a strong effect on the transcriptional landscape of the endometrium (Marla et al and Figure 2 of our manuscript) and exogenous hormone treatment does not synchronize the hormonal cycle between individuals. Below, we present a heatmap showing the expression of menstrual cycle phase markers of the eutopic endometrial epithelial cells (g) and mesenchymal cells (h) from the Tan et al. 2022 single-cell atlas. You can clearly see that the expression of menstrual cycle phase markers between donors strongly differs. Control patient C01 has epithelial markers indicative for early-/ mid-secretory phase, C02 for late-secretory and proliferative phase and C03 for early-proliferative phase.
We and Wang et al. 2020 found thousands of genes to significantly change in expression during the menstrual cycle, including inflammation, adhesion, proliferation and angiogenesis genes. The vast transcriptomic changes during the menstrual cycle are much stronger than the rather subtle endometriosis-specific changes (our Figure 1e and Mareckova et al). The results of Tan et al from comparing 3 control patients with different

menstrual phase markers to endometriosis patients with different hormonal treatments and different menstrual phase markers cannot be considered robust.

We add this heatmap from the Tan et al. atlas as Extended Data Figure 3g,h as an example of the diverse hormonal effects on transcription and justification on why we excluded hormonally treated patients. See also response to Reviewer 2’s Remarks to the Author.

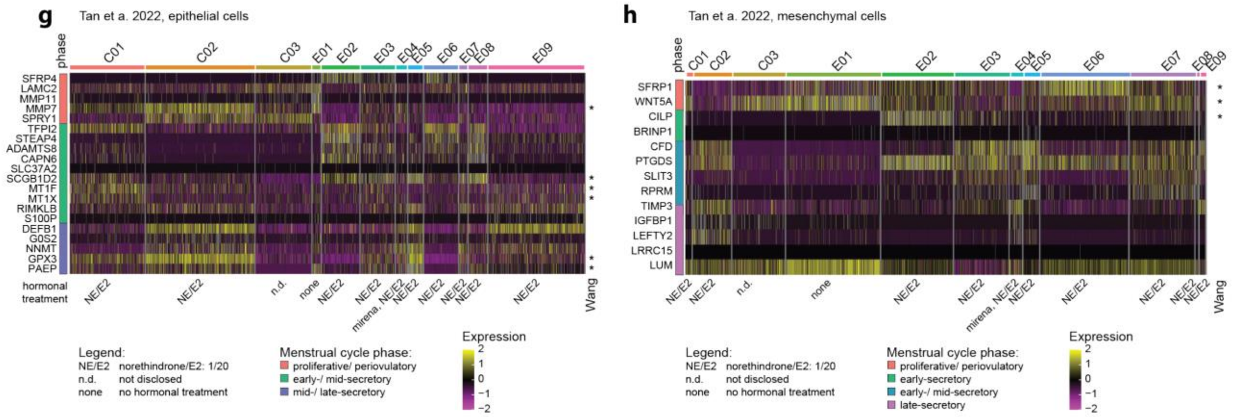

b. While the approach of assessing the least heterogeneous proliferative stage of the menstrual cycle is novel and an important concept, there are other benefits the authors have not mentioned. It could represent the growth phase of relatively new endometriotic lesions during this estrogen regulated phase. Other cycle stages could give even better gene signatures for assessing the initiation of lesions from retrogradely shed endometrium, particularly late secretory and menstrual stages.
We get many significant changes between non-ENDO and ENDO of the secretory phase, but do not consider these robust due to the vast menstrual cycle phase changes in the secretory phase, the imbalance of sub-secretory phases between ENDO and non-ENDO and the subsequent variance that is introduced by the imbalance. If requested, we can add these results with a note of caution to the supplement. We hope that our atlas contributes to bigger consortia of atlases, such as the HECA, to eventually enable such analysis.
We also believe the amount of data that has been generated in this project will stimulate many subsequent studies that can focus on this additional data. To do so now would generate a manuscript too large to be easily comprehensive.

c. Very detailed phenotypic data is provided such as pain scores but these are not correlated to any of the findings.
We wanted to provide these data openly for follow-up studies based on our comprehensive atlas.

d. The practicality of a gene signature from proliferative stage endometrium for a diagnostic test is also not addressed – this is still invasive for girls when endometriosis starts soon after menarche. How else could this approach be used?
We agree with this notion even though this type of endometrial biopsy is considered as non-/ minimal-invasive and applicable to many currently undiagnosed individuals with endometriosis symptoms. Our main efforts are currently to develop a diagnostic product, based on bulk methods, to facilitate diagnosis of all the undiagnosed cases that feel comfortable with an endometrial biopsy. At a later stage, such a diagnostic product might be translatable to menstrual efflux in future.

12. Methodology-
a. While the details of the patient cohort imply that the samples came from women who were diagnosed by visual inspection of the pelvic cavity for endometriotic lesions and histological confirmation, this is not stated. Nor if the diagnosis was histologically confirmed.
We stated this only in the first paragraph and added it now also to the methods section (page 28, line 621-623).

b. There is nothing in the Methods on how the pain scores were obtained from individual patients.
We have now added this (page 28, line 619).

c. Please indicate which 2 mesenchymal cell clusters contained cells in G2M cells in the Methods.
Seurat clusters 2 and 4, we added this to the Methods section (page 33, line 726).

d. Statistical methods need a separate description under a separate heading in the Methods section besides brief information given in the Figure legends for some graphed data eg Figs 1e, 2e, 2f, 3e-h, Suppl Fig 4b (Was this corrected for FDR?)
Thank you for pointing out this important subject. We added this information everywhere in the manuscript and adapted S4b.
Comment to 3e-h: In CellChat (DOI: 10.1038/s41467-021-21246-9), significant interactions between two cell groups through a ligand-receptor pair are identified using a permutation test by randomly permuting the group labels of cells 100 times. The author does not recommend further multiple testing. We show only interactions that were considered significant in both ENDO and non-ENDO, with the main focus on interaction strength change between ENDO and non-ENDO.
We removed the word ‘significant’ for the overall enriched pathways found in Figure 3c.

Minor

We thank the reviewer for the attention to detail and addressed these points.

13. Improvements to referencing to adequately address the concept the authors are suggesting

a. Page 3, line 2, cited references 14-19 – 2 references (14, 18) are incomplete in the reference section.

Thanks for noticing, we completed them (page 21, lines 450-461).

b. Page 8 last line 1st para, find a more relevant reference to ENDO (or endometrium or non-cancer) mesenchymal cell migration, adhesion and EMT than ref 33.

It seems there may have been a misunderstanding. While TGFB1 is a well-reported player in the context of the endometrium and endometriosis, to our knowledge, the specific role of TGFB1|1 (TGFB1 induced transcript 1) has not been reported in this context. To enhance clarity, we have added 'in carcinoma' (page 10, line 213).

c. Page 8 para 2 line 3 – replace ref 34 with a non cancer ref, ALK signaling is well known in reproductive biology and more relevant here

Thanks, we changed the reference (page 10, line 217).

d. P 12 Include Stone 1974 in reference section

Changed (page 13, line 277).

14. In Suppl Table 2, A number of cell types and markers are denoted as MPhi2, or T$_N$/T$_(CM)$ etc. What does this terminology mean?

In LaTeX '$_()$/' is used to create a subscript. We changed this.

15. The Volcano plot in Suppl Fig 5 is missing but referred to in the Legend.

We changed this.

16. Page 8 line 4 – "Extended data figure 6c is incorrect – please indicate correct panel.

We changed this (page 10, line 211).

17. Page 10 line 6 – the mechanism is "complex" with different macrophages having different roles rather then the mechanism is "unknown".

We changed this to "convoluted" (page 9, line 184).

18. Page 11 lines 4-5 – clarify what is meant by "endothelial secretory mural cells" as both cell types are distinctive and both are involved in angiogenesis in different ways.

We classified mural cells to the main cell type endothelial cells and added the 'endothelial', so less familiar readers could place the cell type better. We agree with the reviewer and now removed "endothelial" (page 11, line 239).

19. AUCs are reported in the text to one significant figure (often 0.8), but on the graphs are 0.78 or 0.83, which should be used in the text. Should the AUCs reported on p 13 line 4 be 0.75, 0.83, 0.83 as stated in Fig 4c legend?

We changed the reported AUCs now everywhere to 2 digits (pages 13-14). Thanks for pointing out the values on p 13, line 4, which we changed (page 14, line 297 in the updated manuscript).

20. Discussion , p 15 lin 8-9, please add extra text that Progesterone levels peak during the mid secretory stage (when the embryo implants) when decidualization is maximal. The unique endometrial spiral arterioles are the result of vascular development during the secretory stage of the menstrual cycle. Second last line – endometrial growth is 6-10 mm of new functionalis (not 20 mm).

We strongly rewrote the discussion to accommodate the reviewers' valuable comments.

21. Page 24 – were 330 pipelle biopsies used in this study when 60 women were recruited? How many pipelles did each participant provide?

Pipelles from 330 donors were collected for this study, however, with thorough review of clinical data, pathology reports, menstrual cycle information, pipelle quality scores and single-cell quality, only 60 were selected under our stringent exclusion criteria for single-cell capture. We changed the text (page 29, lines 641).

22. P 29 Vision analysis - the first sentence appears incomplete

Thanks, we changed the text accordingly (page 33, line 738).

Reviewer #2:
Remarks to the Author:
Early and accurate diagnosis of endometriosis is crucial for managing this debilitating condition. However, its pathophysiology remains unclear, often leading to delayed diagnosis. To address these issues, the authors created a single-cell atlas of endometrial tissue, encompassing 466,371 cells from 35 endometriosis patients and 25 non-endometriosis patients. Their analysis revealed significant changes in gene expression and ligand-receptor interactions, including increased inflammation, adhesion, proliferation, cell survival, and angiogenesis across various cell types in patient samples. The authors employed the machine learning algorithm CellCnn, utilizing representation learning to identify pertinent biomarkers within the disease model, allowing for the simultaneous assessment and integration of expression changes across hundreds of genes. This approach led to the development of an 11-gene-signature-based model to predict endometriosis severity, potentially offering precise in vitro diagnostic models for future use.

However, several recent papers have conducted similar analyses (e.g., PMID: 36624343; PMID: 35864314; PMID: 38412094). For instance, PMID: 36624343 reports comparable scale single-cell sequencing on endometriosis and non-endometriosis samples, identifying significant gene expression changes and altered receptor-ligand interactions, highlighting processes such as inflammation, proliferation, nerve growth, and angiogenesis. In this manuscript, the authors identified elevated levels of NFkB, JAK1, Tgfb, and ALK in endometriosis, which are known to play roles in inflammation, angiogenesis, and focal adhesion. However, these pathways are not novel discoveries in the context of endometriosis. In fact, the promotion of angiogenesis and immune cell trafficking in endometriosis is already discussed by Tan et al. (PMID: 35864314). The upregulation and possible involvement of CXCL12 in the stem cell niche in endometriosis is also reported by Marečková et al. The activation of survival pathways in epithelial cells is already discussed by Yan et al. (PMID: 38412094). The dysregulation of TGFb, TNF, and VEGF pathways in endometriosis is already reported by Marečková et al. Thus, the innovation that this manuscript brings in is limited.

We feel there might be a misunderstanding. While we agree that there are great single-cell studies comparing the endometrium to endometriosis lesions (e.g. Fonseca et al. (PMID 36624343,)) and others studying menstrual cycle phase changes (Wang et al. 2020 (PMID 32929266), Garcia-Alonso et al. 2021 (PMID 34857954)), we are the first to comprehensively study endometriosis-specific changes within the eutopic endometrium stratified by the donor’s menstrual cycle phase. Thereby, our study addresses the longstanding pathophysiological question of whether there are endometriosis-specific changes in the eutopic endometrium, the tissue of origin for endometriosis, or if the

changes detected between eutopic endometrium and endometriosis lesions only occur in the peritoneal cavity and during lesion formation. We show several deregulated pathways and ligand-receptor interactions in the eutopic endometrium that may enhance endometriosis lesion formation, which offer novel therapeutic targets to prevent recurrence of endometriosis and provide a novel diagnostic approach and models for this challenging-to-diagnose disease.
We rephrased some parts in the manuscript to make this clearer (including lines 60-64 and lines 387-393).

PMID: 36624343 (Fonseca et al.) generated single-cell eutopic endometrium data from 3 control donors (2 post-menopausal and one with exogenous hormone use) and 7 endometriosis patients (2 with exogenous hormone use, 3 in luteal and 2 in follicular phase). We disagree that this should be a comparable scale to our 60 pre-menopausal donors without exogenous hormone use. More importantly though, we did not find that Fonseca et al. compared the transcription of eutopic endometrium between endometriosis and non-endometriosis donors. Eutopic endometrium from control and endometriosis donors seem to be taken together as ‘eutopic endometrium’ without differential analysis between endometriosis status (or hormonal or menstrual status). We kindly ask Reviewer 2 to point out where in that paper endometriosis-specific changes in the eutopic endometrium were shown, in case we missed it.

PMID: 35864314 (Tan et al.) This study compares the expression of the eutopic endometrium between non-endometriosis (n = 3) and endometriosis (n = 9) donors, mostly reported as under hormonal treatment. Control patient C01 has markers indicative for early-secretory phase, C02 for late-secretory phase and C03 for early-proliferative phase. With the drastic expression changes during the menstrual cycle and only a minor impact of endometriosis status (shown in this manuscript (Fig. 1e & 2) and by Mareckova et al.), it is very likely that Tan et al. identified menstrual-phase-related-changes rather than endometriosis-related-changes. (See details in response to Reviewer 1.11.)

PMID: 38412094 (Yan et al.) Whereas this study nicely stratifies by the donor’s menstrual cycle phase, they only compare the expression between non-endometriosis and endometriosis donors in one cell type. We added this reference where appropriate.

PMID: 39198675 (Marečková et al.) suggest a role of CXCL12 in stem cell niche in the endometrium (not in endometriosis as Reviewer 2 states), but in contrast to us, do not show a differential expression of CXCL12 between non-endometriosis and endometriosis donors. Marečková et al. perform DEG analysis on only 4 cell types. While they do exclude hormonal treated donors from their DEG analysis, they did not stratify by the donor’s menstrual cycle. There is an imbalance in the menstrual cycle phase in their

dataset (control: 8 proliferative, 4 secretory; endometriosis: 16 proliferative, 16 secretory), which can explain e.g. changes in menstrual-cycle phase dependent TGFb expression (same paper) by lack of stratification by the donor's menstrual cycle phase.

Comments:

1. Line 109 corresponds to Fig 2: The mesenchymal, epithelial, and endothelial cell types mentioned are not depicted in Figure 2.

All cell types (incl. mesenchymal, epithelial, and endothelial cell types) are depicted in Fig. 2b in the left UMAP titled 'entire atlas'. The annotation of this UMAP with the main and subcell types can be found in Fig. 1c

2. The finding of nerve fiber growth enrichment in endometriosis is noteworthy. It would be beneficial to explore the specific pathways or genes involved in this mechanism .

We thank the reviewer for this comment and added additional details (page 12, lines 260-262).

3. The single-cell transcriptomics analysis revealed numerous intriguing interactions between different cell types mediated by multiple ligand-receptor pairs. For instance, they found significantly enhanced interactions between macrophages and stromal, endothelial, epithelial, and immune cells via TNF-TNFR1/2 signaling in endometriosis; increased interactions of macrophages, dendritic cells, and epithelial cells with various immune cells mediated through ICAM1/2 and its receptors; and strengthened interactions between macrophages and epithelial cells with endothelial cells via chemokines CXCL2, CXCL3, and CXCL8 with ACKR1. These conclusions should be validated using methods that preserve authentic spatial information, such as multiplex protein staining, multiplex RNA staining, spatial proteomics, or spatial transcriptomics.

Quantitative validation of ligand-receptor interactions with spatial methods presents significant challenges. The previously mentioned single-cell endometriosis papers by the Reviewer presenting ligand-receptor interactions do not include such quantitative spatial validation of ligand-receptor interactions, despite recent publication in good journals (Tan et al. 2022 Nature Cell Biology, Yan et al. 2024 Cell Reports). These studies therefore present quantitative DotPlots of the single-cell data for ligand-receptor interactions, similar to our CellChat bubble plots. Additionally, we present a cross-validation approach with DEG analysis, ligand-receptor analysis and annotation-independent AI analysis. E.g. chemokines and angiogenesis/VEGF were identified by the separate DEG analysis, ligand-receptor analysis, and AI gene signature analysis.

In response to this comment, we validated the elevated levels of the chemokine CXCL3 in the endometrium of women with endometriosis using spatial immunohistochemistry (Fig. 4g, Extended Data Fig. 8).

4. The authors developed a representation learning-based machine learning model. They should provide clarity on their validation strategy for the predictive model developed in their study. Specifically, they should outline the methods they intend to use to assess the accuracy, robustness, and generalizability of the model. This may include external validation using independent datasets. Providing details on the validation process will strengthen the reliability and applicability of their predictive model. Otherwise, the application value falls short.
We thank the reviewer for this comment.
As noted in the discussion, we are working on translating our findings into a more cost-effective bulk sequencing or qPCR-approach. We successfully condensed our endometriosis signature to just 7 genes, achieving a specificity of 0.91 and a sensitivity of 0.78 in endometriosis prediction with bulk sequencing data (not shown in this manuscript). As the focus of this paper is the single-cell analysis and pathophysiology of endometriosis, we believe the translation of the model to an easy and cost effective screening methods is a significant project itself and beyond the scope of this project that already presents the largest and most well characterized analysis of endometrium single cell data to date. See also Reviewer 3, point 1 response.

5. The predictive power of each gene within the 11-gene signature should be elucidated.
We added the predictive contribution for each of the 11 genes as Extended Data Fig. 6f.

Reviewer #3:

Remarks to the Author:

Endo is an understudied, although widespread and critical health condition. The paper presents an analysis of the single-cell atlas of endometrial tissue, encompassing ~450,000 cells from 35 endometriosis and 25 non-endometriosis patients. The results show differential expression between cells at a single stage in the menstrual cycle and estimates of altered receptor-ligand interactions in the endometrium of endometriosis patients. The authors apply an AI model to the differentially expressed genes, showing they can predict Endometriosis status.

1. The interpretation and use of an AI model to predict endo status is unclear, and the paper would benefit from a clear justification of the novelty and impact. The authors have already identified differential expression (between case and controls) at the proliferative phase, so it would follow that the expression of those genes will correlate with disease status. Put another way, the interpretation of the prediction results is that collectively fitting DE genes in a model explains variance in the disease status. This would be useful if there was a translational avenue for prediction, and that it could perform against current clinical diagnosis methods. Performing an out-of-sample prediction against current diagnostic criteria would be convincing. As it currently stands, it's unclear what these results add to the DE expression other than a nice confirmation that genes indeed explain some variance in Endo status (shown in the DEG analysis). How this method would be used in practice could also be considered. Is it practical to generate single-cell data for the diagnosis of Endo, for example?

We thank Reviewer 3 for this insightful comment and would like to expand on these raised points.

We realized that our sentence on line 253 (first submission) might have led to some confusion and changed it (page 13, lines 272-273). Our AI models were trained using 4 different feature selection methods (PCA, CorrgPCA, DEGs, HVG). While one of the feature selection methods does rely on DEGs, the other 3 use highly variable genes as input. Highly variable genes capture the intrinsic variability within and across cell populations, providing a more comprehensive view of the underlying biological heterogeneity and, in our case, led to better performance. This can reveal genes displaying subtle patterns that, when combined with other genes, are predictive of the endometriosis status. Another key advantage of using highly variable genes is that we are not relying on annotations (see also Reviewer 3, point 3) and therefore are able to find genes with transcriptional changes in endometriosis patients in a novel subset of cells. Thereby, the Muscat DEG analysis, ligand-receptor analysis, and AI gene selection serve as a cross-validation rather than duplication, reinforcing the importance of the identified pathways and confirming their dysregulation in endometriosis.

To our knowledge, we are the first to develop an endometrial biopsy-based diagnosis method. This is likely due to the subtle endometriosis-specific expression changes in endometrium bulk sequencing (Mortlock et al. 2020) and the recent advances in single-cell technologies, which have only recently made this challenging problem more approachable. Individual DEGs alone do not show sufficient separation to reliably distinguish between endometriosis and non-endometriosis. Therefore, we employed the machine learning algorithm CellCnn, which can assess and integrate expression changes of hundreds of genes simultaneously. CellCnn employs representation learning to identify biomarkers and predict disease status, offering a robust predictive capability.
We acknowledge that single-cell based diagnostics may not be immediately practical due to the current cost, even though costs are steadily decreasing and there are commercially targeted single-cell methods that can be used for a robust and scalable in-vitro diagnostic assay. As noted in the discussion, we are working on translating our findings into a more cost-effective bulk sequencing or qPCR approach. The discovery of our single-cell endometriosis gene signatures in this manuscript was essential for the successful generation of models based on bulk sequencing data (not shown). Further, we successfully condensed our endometriosis signature to just 7 genes, achieving a specificity of 0.91 and a sensitivity of 0.78 in endometriosis prediction with bulk sequencing data (patent application). We considered this topic beyond the scope of the current manuscript, which focuses on single-cell sequencing.

2. Related to this, the variance in the estimation of accuracy parameters will be high given the in-sample cross-validation and small number of samples used for the evaluation (it appears to be n=14). This is observed with AUC <0.8 for 2/5 folds. Performing more folds across different splits would help determine a better estimate of the sample variance of the AUC, which should be discussed and considered.
Thanks for this comment. We included an equal number of controls and endometriosis patients and ensured balance in disease status, disease severity, and sequencing batch. Considering all these criteria, we believe that 5 folds is a good balance between providing a reliable estimate and ensuring comparability between folds.

Given that an out-of-sample prediction is required for any translational or use cases, how have you considered applying this model to other data sets?
Our immediate focus is on translating our best-performing single-cell signature into a more cost-effective bulk sequencing or qPCR approach, as prioritized in our response to Reviewer 3, Point 1. This approach aims to enhance the applicability and scalability of our findings in clinical and translational settings.

3. As a general comment, the analysis of the DEG section would benefit from more analysis. The variability in DEG results between different methods has been well reported,

so assessment with other tools would be helpful. Likewise, other single-cell tools have been shown to induce bias or non-reproducible results, which, used independently, lead to incorrect biological conclusions. A nice example of this is shown in the Lung Atlas paper, in which different integration methods lead to different estimations of DEG genes and cell types. The authors responded by sequentially using a series of tools and an integrated framework to use output from multiple methods. It would be great to see a more thorough analysis of this data.
We thank the reviewer for sharing these ideas and generally supporting the sequential use of the different tools in our study. Due to the Reviewer's mentioned variability, we chose to perform DEG analysis with the muscat framework utilizing edgeR for differential expression analysis in a pseudobulk approach. Pseudobulk methods, especially those utilizing edgeR, have been demonstrated to outperform single-cell methodologies (Squair et al. 2021, Soneson et al. 2018). Additionally, we also tested Seurat's less stringent FindMarkers, which supports the Muscat findings. If wanted, we can add the FindMarkers results as a Supplementary Table.

For example, how do demographics and disease status/metadata drive interindividual variation?
Our DEG analysis reveals that the gene expression profiles between non-ENDO and mild-ENDO are more similar than those between non-ENDO and severe-ENDO (Figure 3a and Supplementary Tables 4 and 5). Despite having one of the largest single-cell endometrium atlases, further stratification by multiple metadata parameters would reduce statistical power. While we agree that stratification by different parameters has potential merit, we believe it is beyond the scope of this project.

What is the role of genetics, and specifically Endo GWAS hits, in influencing expression in different cell types?
Great thought. Regarding the role of genetics, large GWAS studies involving thousands of patients and controls (e.g., Sapkota et al. 2017) have identified several gene variants associated with endometriosis. While genetic variants significantly correlate with endometriosis, their overall contribution is thought to be relatively low (e.g., Nouri et al. 2010) and influenced by multiple variants and other factors rather than a single "endometriosis gene." Our single-cell patient cohort is far from being large enough to perform detailed expression analysis in different cell types stratified by specific gene variants and their combinations. Interestingly though, our ligand-receptor analysis showed increased interaction between VEGFA and VEGFR2, and genetic variants in the VEGFR2 gene (KDR) have previously been linked to endometriosis susceptibility (Seton-Rogers 2007).

Can you extend beyond cell types to identify disease-associated cell states?

Great question. One of the advantages of using ScaiVision is that we are able to identify cells driving the prediction of our model (Figure 4e). Notably, we were able to discover specific subtypes of mural cells, macrophages, and ciliated epithelial cells to be crucial for non-ENDO prediction and mural cells, dendritic cells, and several epithelial cell types for ENDO prediction (Fig. 4e, Supplementary Table 7).

And what co-regulatory networks are perturbed (or not) in disease?
In our Figure 5, we illustrate the perturbed pathways and how some of them interact. We did not notice interesting transcription factors in the DEG data that would perturb co-regulatory networks.

4. I understand how the confounding in the imbalance of case/control, changes in cell numbers, and menstrual status will make analysing non-proliferative stages challenging. Still, it feels as though cells from donors at periovulatory and secretory stages have been ignored. The analysis showing changes in cell types between stages is useful but needs to report new biology. This analysis shows significant changes in some cell types, but from my knowledge, this is known already.
Currently, Wang et al. 2020 serves as an excellent reference for menstrual cycle phase staging, with 19 healthy donors with a regular menstrual cycle and no intake of external hormones (Fluidigm C1 single-cell set). They only report 48 to 173 cells per donor (Wang Extended Data Fig. 1c), menstrual cycle markers only for stromal and epithelial cells, and do not report progesterone levels and HE staining results, which are commonly used to guide menstrual cycle phase staging in clinics.
Our identified menstrual cycle phases with refined evaluation (serum progesterone, cycle day, HE staining and transcriptomic profile) has excellent correspondence to the phases described in Wang et al. 2020. The main advantage of our atlas is that we have 50 times more cells per patient (average 7773) and a detailed annotation with menstrual cycle specific cell types and marker genes also for endothelial, lymphoid and myeloid, which can well serve as reference for future studies. We believe it is out of scope for this study with focus on endometriosis-associated changes and diagnostic models.
We devoted an entire figure to the changes during the menstrual cycle specifically to highlight the challenges in analyzing endometriosis-associated changes in periovulatory and secretory stage samples. We strongly believe that considering the significant variations in cell types and pathways throughout the menstrual cycle is crucial for accurately analyzing disease-specific changes. Unfortunately, this approach is not yet widely adopted in the field (see responses to Reviewer 2). We hope that, after reading this manuscript, researchers will recognize the importance of stratification by menstrual cycle phase and incorporate this approach into their research.
As written in the Reviewer 1, point 11b response, we get many significant changes between non-ENDO and ENDO of the secretory phase and if requested, we can add

these results with a note of caution to the supplement. We hope that our atlas contributes to bigger consortia of atlases, such as the HECA, to eventually enable such analysis.

5. The work would benefit significantly from the validation of some of the results. For example, in lines 147-148, it's noted that the interplay between different cell types through secreted cytokines is critical in inflammatory environments. This is well known. The results presented here show that CXCL2, CXCL3, TGFB1, and CXCL12 are co-upregulation (in endo) with IL-3, IL-11, and IL-8. It's unclear what is novel here or how these results confirm the onset or progression of endometriosis. For example, what is the impact on the disease (model systems) of inhibiting the expression of these specific cytokines? Similarly, it's suggested that the upregulation of TGFB1 and JAK1 are responsible for angiogenesis through TGFb cytokine signalling. Validating the impact of the cell phenotype/behaviour (angiogenesis in this case) on the up/down-regulation of TGFB1 and JAK1 would help convince the community that the analysis of the single-cell data has led to new biology of endometriosis being uncovered.
Even though interesting, for such a proof we would need complicated co-culture organoid systems with endothelial/epithelial/mesenchymal and immune cells. Such model systems with immune cells are challenging, potentially not stable and we do not have them in our laboratory.

6. Results (such as line 203) are presented as significant with $p<0.05$. But in all instances, multiple tests have been performed. Are the significance thresholds adjusted for multiple testing?
We thank the reviewer for pointing this out. We added better descriptions in the text where appropriate. See also our answer to Reviewer 1.11d.

Point-by-point Response to Reviewer's Comments

We thank the reviewers for their excellent, extensive, detailed, and helpful feedback on our manuscript. Your insights not only helped improve our manuscript but also provided stimulating input for our research and inspired interesting discussions. We deeply appreciate your time and effort in reviewing our work and are grateful for your valuable contributions. Below, we provide a detailed response to each comment.

Reviewer #1 (Remarks to the Author):

The authors have adequately addressed all my comments and the manuscript has improved as a result. In particular, the Discussion is more focused and powerful.

We would like to express our sincere gratitude to Reviewer #1 for the highly constructive feedback provided. Your detailed and thoughtful comments have been instrumental in improving the quality of our manuscript. We appreciate your recognition of the enhanced focus and strength of our Discussion section, which was largely shaped by your insightful suggestions. A huge thank you for your time and effort to reviewing our work.

Reviewer #3 (Remarks to the Author):

1. Two reviewers have flagged other published work that has shown variation in cell composition and single-cell analysis of endo samples. It's argued that the novelty here is that this work "the first to comprehensively study endometriosis-specific changes within the eutopic endometrium stratified by the donor's menstrual cycle phase". However, as flagged by another reviewer, there are substantial gaps in samples across relevant tissues that are highly relevant across certain stages of the cycle and predominantly focus on a single stage (proliferative). I appreciate there may be some practical reasons for not including these tissue samples and missing stages, but it undermines the point of novelty.

In response to the reviewer's concern, we acknowledge the limitations of the main flagged study by Tan et al. (PMID: 35864314), which is based on a small number of non-ENDO eutopic samples (n = 3) with mixed menstrual cycle phase markers (early-/mid-secretory, late-secretory, and proliferative), compared to 9 ENDO eutopic samples also with mixed menstrual cycle phase markers (See our Extended Data Fig. 3 g, h). Given the substantial fluctuations in the menstrual cycle and comparatively weaker changes related to endometriosis, we believe that stratifying the analysis by the donor's menstrual cycle phase is crucial for obtaining reliable and meaningful results.

Therefore, we argue that our phase-specific approach offers valuable, novel contributions to the field, providing new insights into endometriosis-specific changes (see also response to Reviewer #4, point 1). Through single-cell analysis of the endometrium, we identify cell-type-specific regulatory mechanisms, confirming known pathways like TNF and VEGFA, while revealing novel interactions in macrophages, immune cells, endothelial cells, and epithelial cells. This approach, utilizing our large single-cell atlas and menstrual cycle phasing, enables exploration of these interactions in ways not previously reported, offering deeper understanding and potential therapeutic targets. Furthermore, our study introduces a novel approach by leveraging predictive models based on single-cell data from the endometrium and gene signatures for endometriosis. This innovative method distinguishes our work from previous studies and lays the groundwork for future hypothesis generation and clinical advancements, despite the need for further external validation. By stratifying the analysis by donor menstrual cycle phase, we offer a more robust and reliable analysis, enhancing the potential for future applications.

To address the reviewer's concern about secretory phase (also first review round, point 4.), we performed DEG analysis on a set of secretory-phase samples with comparable clinical data. These include 4 non-Endo and 4 Endo samples, with 2 early-secretory and 2 mid-secretory samples from each group. The analysis resulted in a large number of DEGs (n = 801, p.val adj. 0.05), predominantly in epithelial cells. However, the substantial

changes in epithelial cell expression during the secretory phase (early, mid and late, Fig. 2 and Extended Data Fig. 4) complicate the distinction between menstrual cycle-related and endometriosis-driven changes. Gene Ontology (GO) analysis of these DEGs gave limited insight. Some interesting terms were enriched such as cell adhesion and migration and axon regeneration in epithelial cells, cytokine production and signaling as well as interferon response in endothelial cells, and focal adhesion in mesenchymal cells (full list for now here: endometriosis_endometrium_scRNA_atlas/Tables/GO_enrichment_Secretory_E065A005.csv). Most enriched terms however yielded broad and non-specific findings, including enriched terms such as related to mitochondria, cellular respiration and oxidative phosphorylation, making their relevance to endometriosis pathophysiology unclear.

While these findings suggest some potential differences between Endo and non-Endo in the secretory phase, we feel the results are not robust enough to form a compelling narrative for the main manuscript. Therefore, we have opted not to include this data in the main text. However, should the editor deem it valuable, we are happy to provide the secretory-phase DEG and GO tables as supplementary material, with a cautionary note regarding the observed variability. We hope that our atlas contributes to larger consortia of atlases, such as HECA, which will eventually enable such rigorous analyses throughout the menstrual cycle phases in the future.

2. The finding of nerve fiber growth enrichment in endometriosis is noteworthy. It would be beneficial to explore the specific pathways or genes involved in this mechanism. The revision noted additional details have been added (lines 260-262). However, this consists of mentioning one of the differentially expressed genes.

Thank you for your feedback. We agree that the finding of nerve fiber growth enrichment in endometriosis is noteworthy. The mention of CXCL12 and potential pathway in lines 260–262 was included as an illustrative example. Following the editor's guidance, we kept this section concise to avoid overinterpretation while accurately presenting the findings. We hope this addresses your concern.

3. A critical comment raised by multiple reviewers is the necessity of out-of-sample validation for the prediction model. The argument given is that this is out of scope for this work, which instead focuses on "the single-cell analysis and pathophysiology of endometriosis". However, the title, abstract, introduction, results and discussion all highlight diagnosis and prediction as points of novelty "we are the first to develop an endometrial biopsy-based diagnosis method". Given this, I strongly encourage the authors to consider developing this work, as a small N within the sample and small K cross-validation are not statistically robust enough to estimate AUC accurately. Coupled with variation in patient samples of course.

We appreciate the reviewer's comment and acknowledge the importance of out-of-sample validation for prediction models. While our primary focus is indeed on the pathophysiology of endometriosis, we have carefully revised the manuscript to acknowledge that while the developed models to predict endometriosis would need further external validation, they can still serve as a basis for hypothesis generation and guide future clinical advancements. Our work introduces a novel approach by combining neural networks and single-cell analysis of the endometrium as an initial step towards the prediction of endometriosis. We agree that further validation, including larger sample sizes, will be essential for future development. Our immediate focus is on translating our best-performing single-cell signature into a more cost-effective bulk sequencing or qPCR approach, to enhance the applicability and scalability of our findings in clinical and translational settings. We are committed to addressing the statistical concerns raised by the reviewer in future iterations of this project.

4. The sample variance of AUC across folds is high. Performing more folds will help accurately estimate this variance.

Thank you for this comment. While it is possible to use more folds, we opted for 5 cross-validation folds to balance the limitations of our dataset size while ensuring that the holdout sets remain diverse and well-balanced across different criteria (SFig. 6a-c). This choice allows us to achieve a good trade-off between computational efficiency and robust generalization estimation. Under the constraints of stratified selection, further folds would turn out very similar to the 5 selected ones.

5. Reviewers suggested looking at alternative DEG and integrating DEG methods to account for method bias. This is a well-established issue in the field and likely leads to biased results.

To respond to this comment, we have performed DEG analysis with one of the most frequently used methods, the Seurat function FindMarkers, using the same configuration as for the Muscat DEG analysis in the manuscript comparing patients diagnosed with endometriosis to the control population. A high number of DEGs were found (n = 10 518). This is concordant with the finding that methods operating at the single-cell level yield a high number of DEGs, likely increasing the risk of false positives (https://www.nature.com/articles/s41467-021-25960-2). In contrast, methods such as Muscat, which are specifically tailored to sample-level predictions, produce more reliable results with better type I error control (https://www.nature.com/articles/s41467-021-25960-2#Sec10).

Nonetheless, a good overlap between the two methods was observed, with 40% of the cell type DEGs identified through Muscat shared with the FindMarkers analysis (n=126). All 11 genes shared by ScaiVision models and the DEG analysis from Muscat were also

found differentially expressed when using FindMarkers. Genes uniquely identified by Muscat have generally a lower fold change (p_val < 2.2e-16 using Wilcoxon rank-sum test, greater alternative). FindMarkers filters out by default genes with a fold change below 0.1, which may explain why these genes were not detected by FindMarkers.

6. Can you extend beyond cell types to identify disease-associated cell states, not just cell types?

Our approach extends beyond cell type identification by leveraging ScaiVision's ability to rank cells based on disease predictiveness, helping to identify disease-associated cell states. For example, we found that a high percentage of mural cells, dendritic cells, and various epithelial cell types were found among the top 10% of predictive cells (Fig. 4e, Supplementary Table 7). These findings align with altered gene expression and ligand-receptor interactions seen in disease (Fig. 3), as well as increased VEGF signaling in mural cells (Fig. 3g).

In response to the reviewer's question, we performed an additional analysis comparing the top 10% of endometriosis-associated predictive cells to the rest of the cells within these cell-type. We found angiogenesis, regulation of cell adhesion, endothelial cell migration and response to cytokine to be enriched in mural cells, inflammatory response, lysosome and regulation of programmed cell death was upregulated in macrophages and cilium assembly, organization and movement was upregulated in ciliated cells(full table added for now in endometriosis_endometrium_scRNA_atlas\Tables\GO_enrichment_predictive_cells_E066A002.csv). These findings are in line with the results described in the manuscript.

Moreover, when examining the UMAP and applying various clustering methods, we did not observe any distinct clusters segregated strictly by disease status (ENDO vs. non-ENDO) when excluding sample and menstrual cycle phase-driven effects. If not captured in our analysis, we would appreciate further clarification from the reviewer on the specific type of disease-associated cell states they are referring to.

7. All reviewers suggested validation of some biological results. Given the well-established biases of the analysis methods, this remains something that would be incredibly valuable.

In response to the reviewers' suggested validation of biological results, we confirmed the differential expression of CXCL3 with immunohistochemistry (Fig. 4g, Extended Data Fig. 8).

8. Reviewer #3 asked about significance thresholds and if multiple testing correction has been performed. The rebuttal pointed to the reply to Reviewer #1 comment 11d. This

comment relates to the practicality of a gene signature from proliferative stage endometrium for a diagnostic test. It's not clear what has been changed, or indeed if multiple testing correction was performed.

We apologize for the oversight in our previous response. We intended to refer to Reviewer #1 comment 12d, not 11d. Here in detail:

Thank you for pointing out this important subject. We added this information everywhere applicable in the manuscript (Fig. 1e, Fig. 2e and f, Fig. 3a, b, Fig. 4d, Extended Data Fig. 1e and f, Extended Data Fig. 4b and Extended Data Fig. 7e). For the DEG analysis with muscat, we used a cut-off value of 0.05 of the local adjusted p.value given by muscat.
Comment to Fig. 3e-h: In CellChat (DOI: 10.1038/s41467-021-21246-9), significant interactions between two cell groups through a ligand-receptor pair are identified using a permutation test by randomly permuting the group labels of cells 100 times. The author does not recommend further multiple testing. We show only interactions that were considered significant in both ENDO and non-ENDO, with the main focus on interaction strength change between ENDO and non-ENDO.
We removed the word 'significant' for the overall enriched pathways found in Figure 3c.

Reviewer #3 (Remarks on code availability):

Code is available for some areas of analysis. Data paths are hard coded and not not commented, so reproducing analysis would be challenging

We greatly appreciate the reviewers' constructive comments regarding code availability and reproducibility. In response, we have made significant improvements to the GitHub repository. Specifically, we have reorganized the structure to enhance clarity and ease of use. Detailed descriptions have been added to the repository, as well as the code to reproduce the figures and supplementary data figures. Furthermore, we have included the necessary data paths, provided links for downloading the relevant data, and added clear instructions on how to reproduce the results presented in the paper. Additionally, we have populated the author's GitHub profile to facilitate better access to the relevant resources. We believe these updates will significantly improve the transparency and reproducibility of the analysis.

Reviewer #4 (Remarks to the Author):

The author's response has made a commendable effort to articulate the uniqueness of their study and to compare it in detail with previous research. However, there are still some issues:

1.Previous review comments mentioned that the pathophysiological mechanisms discussed in the study are not novel. While the author's response lists their unique dataset and methods, it does not sufficiently emphasize how these methods bring new insights into the pathophysiological mechanisms. Moreover, the previously mentioned review comments noted that the biological pathways and molecular mechanisms have been extensively reported in studies on endometriosis. The authors' response should specifically point out what new regulatory mechanisms or modes of action their study has discovered in these known pathways, particularly highlighting how their single-cell data reveal the cell type specificity of these processes.

While we acknowledge the reviewer's point, we would like to clarify that some of the comments raised by Reviewer 2 regarding the novelty of our findings were incorrect, as detailed in our responses to Reviewer 2. Our study contributes novel insights into the pathophysiology of endometriosis by identifying cell-type-specific regulatory mechanisms through comprehensive single-cell analysis of the endometrium. While we confirm the involvement of some known biological pathways such as TNF and VEGFA, our research provides unprecedented detail on the cell-type-specific interactions and regulatory mechanisms. The use of our large single-cell atlas, precise cell annotations, and menstrual cycle phasing enables us to explore these interactions in ways not previously reported.

Key findings include:

Inflammation and Adhesion:

Our study reveals a macrophage mediated enhanced inflammation in the endometrium of women with endometriosis through a significant increase in subtypes MΦ4 and MΦ5 interactions with structural endometrial cell via TNF–TNFR1/2 signaling. This signaling pathway is known to activate the NFκB and MAPK pathways, which we observed to be elevated. We also demonstrate heightened macrophage-dendritic cell-epithelial cell interactions with immune cells through ICAM1/2- (ITGA*+ITGB2)/SPN, potentially increasing the recruitment of circulating leukocytes and contributing to an inflammatory microenvironment that favors lesion formation. Furthermore, we uncover an enhanced interaction between macrophage subtypes and epithelial cell subtypes with endothelial cell subtypes through the chemokines CXCL2, CXCL3, CXCL8 with ACKR1, known to regulate inflammatory response.

Proliferation and Cell Survival:

We identify a novel reduced signaling interaction between the cell death receptor TMEM219 and IGFBP3 in eutopic mesenchymal cells in endometriosis. Coupled with upregulated NFκB and ALK signaling, this reduced signaling may contribute to enhanced cell survival and proliferation, aiding the establishment and progression of endometriotic lesions.

Angiogenesis:

Our study is the first to detail the cell-type and receptor-specific interactions in VEGF signaling, highlighting the significant roles for macrophages and endothelial cells through VEGFA-VEGFR1/-VEGFR2, as well as mural cells and endothelial cells through PGF-VEGFR1 interactions. We show a significant increase in secretory mural cell abundance in the endometrium of women with endometriosis aligning with endothelial cell proliferation via enhanced VEGF signaling in ENDO. These findings in the endometrium suggest an early link to stem cell behavior raising the potential perivascular endometrial mesenchymal stem cell transformation may already be occurring in the endometrium, providing critical and novel support for the ‘stem cell hypothesis’ of endometriosis pathogenesis.

We also observe an increase in TGFβ signaling and glycolysis/gluconeogenesis pathways, suggesting a Warburg-like effect in the eutopic endometrium, which could further promote angiogenesis. While TGFβ signaling in endometriosis has been reported previously, our work provides additional evidence for its role in these processes in the endometrium.

Nervous System and WNT/NOTCH Pathways:

Our analysis additionally reveals a microenvironment in the endometrium of women with endometriosis conductive of nerve fiber infiltration, driven by axon guidance-related genes. Increased expression of these genes supports the presence of nerve fibers in the endometrium (Al-Jefout et al., 2009, doi:10.1093/humrep/dep275). If refluxed into the peritoneal cavity, this provides a mechanism for the lesions to interact with existing nerve fibers, potentially facilitating a feedback loop that contributes to painful sensations. Furthermore, we observe subtle increases in WNT signaling (WNT2/3/7A - (FZD3/4/6+LRP6) and NOTCH signaling, which may influence epithelial differentiation in the endometrium, a feature previously observed in endometriosis lesions.

In summary, our single-cell analysis uncovers new regulatory mechanisms in known pathways as well as new interactions, revealing how macrophages, immune cells, endothelial cells, and epithelial cells interact in a highly specific and refined manner.

These findings provide a deeper understanding of the cellular and molecular processes driving endometriosis and highlight potential therapeutic targets for future research.

2.Your response briefly mentions the SDF-1α/Rho/mDia pathway influenced by CXCL12. However, it lacks a detailed discussion on how this pathway specifically contributes to the pathological nerve growth observed in endometriosis. If possible, please include new data or specific results from the study that show the differential expression of CXCL12 and its impact on nerve growth. If the study includes pathway analysis, highlighting how these pathways are enriched in endometriosis samples compared to controls would be valuable.

Thank you for your detailed feedback. The mention of the SDF-1α/Rho/mDia pathway influenced by CXCL12 was intended as a brief reference to its potential mode of action, given its established role in axon pathfinding. While our study includes pathway enrichment analysis that highlights axon guidance and nervous system development as significant terms in ENDO mesenchymal cells, the current findings do not definitively demonstrate a causal link between CXCL12 and nerve growth and we chose not to overextend our interpretation.

3.The reviewer suggested the use of spatial proteomics or spatial transcriptomics techniques to validate the interactions between cells. The author mentioned using spatial immunohistochemistry to validate the expression of CXCL3. Although this is a good start, the author did not specify how this method compares with other spatial techniques recommended by the reviewer, nor whether other spatial data analysis techniques were considered or planned for use in the study. Additionally, the author mentioned cross-validation methods in their response, including differential gene expression (DEG) analysis, ligand-receptor analysis, and AI analysis. However, they did not provide detailed descriptions of how these methods were specifically applied to their dataset, nor how these analyses support their conclusions interactively.

Quantitative validation of ligand-receptor interactions with spatial methods indeed presents significant challenges. As mentioned, we validated the expression of CXCL3 in the endometrium of women with endometriosis using spatial immunohistochemistry. At this stage, we do not plan to perform additional spatial techniques beyond immunohistochemistry.

To support our findings comprehensively, we implemented cross-validation methods, including differential gene expression (DEG) analysis, ligand-receptor interaction analysis, and annotation-independent AI gene signature analysis. These methods complement each other to reinforce our conclusions. For instance, key biological processes such as chemokine signaling and angiogenesis/VEGF pathways were consistently identified through DEG analysis, ligand-receptor predictions, and AI-based

gene signature analysis. This convergence of evidence strengthens the validity of our findings.

The interactive conclusions from these analyses are summarized in Fig. 5, which integrates the insights from the various approaches applied in this study. While we recognize the broader utility of spatial data analysis techniques, we believe that our current approach, combining computational cross-validation methods with the targeted spatial validation, sufficiently supports the main conclusions of our work.

4.The author mentions specificity and sensitivity achieved with bulk sequencing data but does not detail the specific validation techniques used for the machine learning model developed from single-cell data, which is the focus of the paper. Moreover, the author does not discuss how the model performs under different conditions or with different datasets. Information on how the model handles variability in data, which is crucial for demonstrating robustness and generalizability, is lacking. In addition, the reviewer suggested using external validation with independent datasets as a way to strengthen the reliability and applicability of the predictive model. The author's response does not address whether such external validation has been or will be conducted.

The main focus of the paper is the pathophysiology of endometriosis. Suitable comparable single-whole-cell datasets for endometrial tissue are currently not available in a high enough number for a thorough external validation. We could, however, successfully translate the signature to bulk RNA-seq by training standard machine learning models using our single-cell signatures as well as distilling our best-performing single-cell signature through iterative gene selection into a core gene set suitable for a more cost-effective qPCR approach and large-scale clinical validation study. The specificity and sensitivity achieved with bulk sequencing data were only mentioned in a review response (Reviewer #2, point 4) and the editor did not favor its inclusion. To clarify this point in the manuscript, we have expanded the discussion to include how the external validation would be conducted.

Reviewer #4 (Remarks on code availability):

After examining the code, I realised that reproducing the results in the paper was impossible. The github links lacked an introduction to the data as well as a download link, and there was no introduction to the code, such as what each code was used for, or to reproduce which figure from the paper. The author's github profile is blank, which is not conducive to reproducing the results of the paper.

We greatly appreciate the reviewers' constructive comments regarding code availability and reproducibility. In response, we have made significant improvements to the GitHub repository. Specifically, we have reorganized the structure to enhance clarity and ease of

use. Detailed descriptions have been added to the repository, as well as the code to reproduce the figures and supplementary data figures. Furthermore, we have included the necessary data paths, provided links for downloading the relevant data, and added clear instructions on how to reproduce the results presented in the paper. Additionally, we have populated the author's GitHub profile to facilitate better access to the relevant resources. We believe these updates will significantly improve the transparency and reproducibility of the analysis.

Point-by-point Response to Reviewer's Comments

We thank the reviewers for their excellent, extensive, detailed, and helpful feedback on our manuscript. Your insights not only helped improve our manuscript but also provided stimulating input for our research and inspired interesting discussions. We deeply appreciate your time and effort in reviewing our work and are grateful for your valuable contributions. Below, we provide a detailed response to each comment.

Reviewer #1 (Remarks to the Author):

The authors have adequately addressed all my comments and the manuscript has improved as a result. In particular, the Discussion is more focused and powerful.

We would like to express our sincere gratitude to Reviewer #1 for the highly constructive feedback provided. Your detailed and thoughtful comments have been instrumental in improving the quality of our manuscript. We appreciate your recognition of the enhanced focus and strength of our Discussion section, which was largely shaped by your insightful suggestions. A huge thank you for your time and effort to reviewing our work.

Reviewer #3 (Remarks to the Author):

1. Two reviewers have flagged other published work that has shown variation in cell composition and single-cell analysis of endo samples. It's argued that the novelty here is that this work "the first to comprehensively study endometriosis-specific changes within the eutopic endometrium stratified by the donor's menstrual cycle phase". However, as flagged by another reviewer, there are substantial gaps in samples across relevant tissues that are highly relevant across certain stages of the cycle and predominantly focus on a single stage (proliferative). I appreciate there may be some practical reasons for not including these tissue samples and missing stages, but it undermines the point of novelty.

In response to the reviewer's concern, we acknowledge the limitations of the main flagged study by Tan et al. (PMID: 35864314), which is based on a small number of non-ENDO eutopic samples (n = 3) with mixed menstrual cycle phase markers (early-/mid-secretory, late-secretory, and proliferative), compared to 9 ENDO eutopic samples also with mixed menstrual cycle phase markers (See our Extended Data Fig. 3 g, h). Given the substantial fluctuations in the menstrual cycle and comparatively weaker changes related to endometriosis, we believe that stratifying the analysis by the donor's menstrual cycle phase is crucial for obtaining reliable and meaningful results.

Therefore, we argue that our phase-specific approach offers valuable, novel contributions to the field, providing new insights into endometriosis-specific changes (see also response to Reviewer #4, point 1). Through single-cell analysis of the endometrium, we identify cell-type-specific regulatory mechanisms, confirming known pathways like TNF and VEGFA, while revealing novel interactions in macrophages, immune cells, endothelial cells, and epithelial cells. This approach, utilizing our large single-cell atlas and menstrual cycle phasing, enables exploration of these interactions in ways not previously reported, offering deeper understanding and potential therapeutic targets. Furthermore, our study introduces a novel approach by leveraging predictive models based on single-cell data from the endometrium and gene signatures for endometriosis. This innovative method distinguishes our work from previous studies and lays the groundwork for future hypothesis generation and clinical advancements, despite the need for further external validation. By stratifying the analysis by donor menstrual cycle phase, we offer a more robust and reliable analysis, enhancing the potential for future applications.

To address the reviewer's concern about secretory phase (also first review round, point 4.), we performed DEG analysis on a set of secretory-phase samples with comparable clinical data. These include 4 non-Endo and 4 Endo samples, with 2 early-secretory and 2 mid-secretory samples from each group. The analysis resulted in a large number of DEGs (n = 801, p.val adj. 0.05), predominantly in epithelial cells. However, the substantial

changes in epithelial cell expression during the secretory phase (early, mid and late, Fig. 2 and Extended Data Fig. 4) complicate the distinction between menstrual cycle-related and endometriosis-driven changes. Gene Ontology (GO) analysis of these DEGs gave limited insight. Some interesting terms were enriched such as cell adhesion and migration and axon regeneration in epithelial cells, cytokine production and signaling as well as interferon response in endothelial cells, and focal adhesion in mesenchymal cells (full list for now here: endometriosis_endometrium_scRNA_atlas/Tables/GO_enrichment_Secretory_E065A005.csv). Most enriched terms however yielded broad and non-specific findings, including enriched terms such as related to mitochondria, cellular respiration and oxidative phosphorylation, making their relevance to endometriosis pathophysiology unclear.

While these findings suggest some potential differences between Endo and non-Endo in the secretory phase, we feel the results are not robust enough to form a compelling narrative for the main manuscript. Therefore, we have opted not to include this data in the main text. However, should the editor deem it valuable, we are happy to provide the secretory-phase DEG and GO tables as supplementary material, with a cautionary note regarding the observed variability. We hope that our atlas contributes to larger consortia of atlases, such as HECA, which will eventually enable such rigorous analyses throughout the menstrual cycle phases in the future.

2. The finding of nerve fiber growth enrichment in endometriosis is noteworthy. It would be beneficial to explore the specific pathways or genes involved in this mechanism. The revision noted additional details have been added (lines 260-262). However, this consists of mentioning one of the differentially expressed genes.

Thank you for your feedback. We agree that the finding of nerve fiber growth enrichment in endometriosis is noteworthy. The mention of CXCL12 and potential pathway in lines 260–262 was included as an illustrative example. Following the editor’s guidance, we kept this section concise to avoid overinterpretation while accurately presenting the findings. We hope this addresses your concern.

3. A critical comment raised by multiple reviewers is the necessity of out-of-sample validation for the prediction model. The argument given is that this is out of scope for this work, which instead focuses on “the single-cell analysis and pathophysiology of endometriosis”. However, the title, abstract, introduction, results and discussion all highlight diagnosis and prediction as points of novelty “we are the first to develop an endometrial biopsy-based diagnosis method”. Given this, I strongly encourage the authors to consider developing this work, as a small N within the sample and small K cross-validation are not statistically robust enough to estimate AUC accurately. Coupled with variation in patient samples of course.

We appreciate the reviewer's comment and acknowledge the importance of out-of-sample validation for prediction models. While our primary focus is indeed on the pathophysiology of endometriosis, we have carefully revised the manuscript to acknowledge that while the developed models to predict endometriosis would need further external validation, they can still serve as a basis for hypothesis generation and guide future clinical advancements. Our work introduces a novel approach by combining neural networks and single-cell analysis of the endometrium as an initial step towards the prediction of endometriosis. We agree that further validation, including larger sample sizes, will be essential for future development. Our immediate focus is on translating our best-performing single-cell signature into a more cost-effective bulk sequencing or qPCR approach, to enhance the applicability and scalability of our findings in clinical and translational settings. We are committed to addressing the statistical concerns raised by the reviewer in future iterations of this project.

4. The sample variance of AUC across folds is high. Performing more folds will help accurately estimate this variance.

We thank the reviewer for this valuable point. In response, we conducted additional analysis to clarify the source of the variance across folds, as detailed below. Based on this, we explain why we do not believe that increasing the number of folds would yield a more stable or reliable estimate in our specific setting.

First, in the AUC plots each point represents a single model trained and evaluated within a nested cross-validation framework. Thus, **the observed AUC variance reflects model-level performance (not sample-level)** across training, validation, and evaluation sets, hence there is no metric of "sample variance". Nonetheless, we recognize that, particularly for the validation and evaluation AUCs, there is a high variance both within and between folds. The intra-fold variance—i.e., variation within the boxplots—arises largely from differences in hyperparameter settings and random seeds, which is expected during model tuning. Inter-fold variance, on the other hand, stems from differences in sample composition across folds, particularly the inclusion of samples that are consistently difficult to classify.

To better understand this, we performed a **sample-level misprediction analysis across all folds and CV splits**, revealing that validation and evaluation folds with lower AUCs tend to be enriched for specific outlier samples. For example, fold 5 showed reduced evaluation AUCs across all dimensionality reduction methods and contained samples 83, 95, 96, and 123, all of which were particularly difficult to predict, in some cases displaying a misprediction rate of up to 100%. Similar patterns were observed in the validation fold 2, exhibiting the lowest median AUC across different dimension reduction techniques. This fold contained samples 70, 116, and 125 in at least 3 of the 5 CV splits. Notably,

these specific samples had a high misprediction rate of at least 25%. These results suggest that variance is driven by a subset of biologically ambiguous or borderline cases.

Interestingly, samples prone to misprediction are predominantly samples from non-endometriosis (non-ENDO) patients. These non-ENDO patients are not strictly healthy patients, with some exhibiting symptoms of endometriosis but without visible lesions during laparoscopy. Therefore some non-ENDO samples may share transcriptomic features with ENDO, like inflammation, which will make them harder to classify accurately. Moreover, there is no clear pattern of higher levels of misprediction for either more mild or more severe endometriosis samples. The pattern of misprediction also does not appear to be predominantly due to a batch effect, although certain batches (e.g. batch 18) are enriched for mispredicted samples, as all batches also contain some samples that were well-predicted.

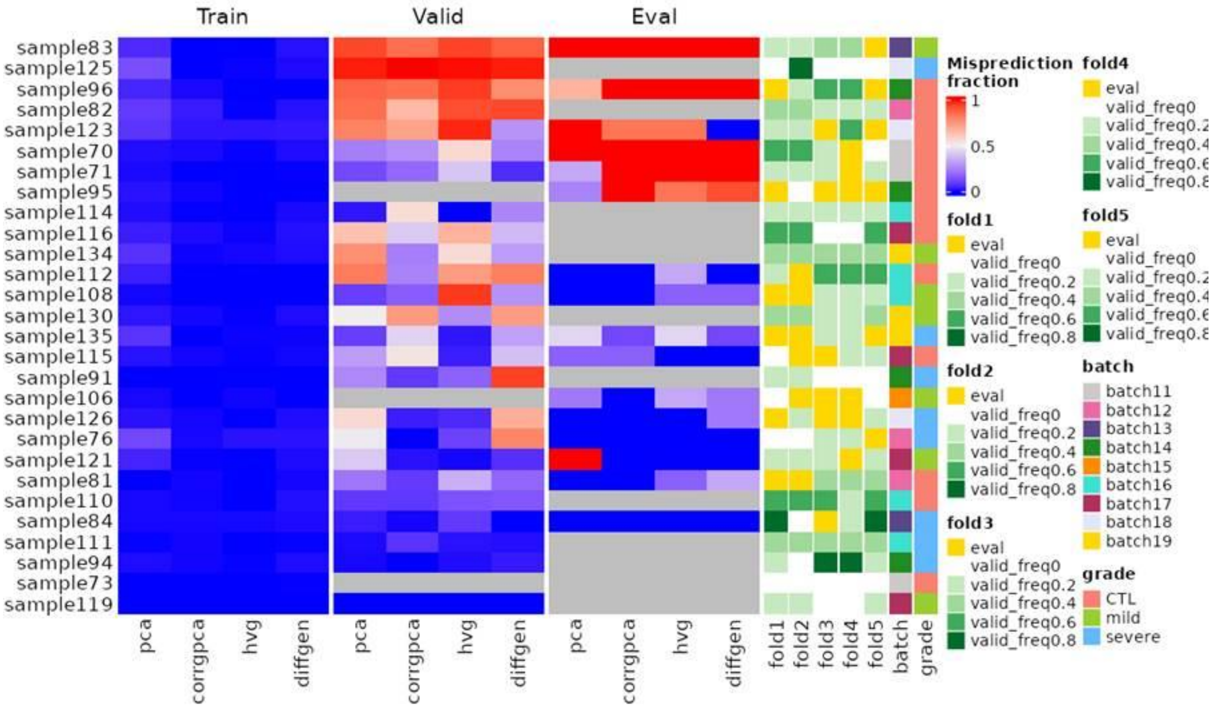

Misprediction of samples computed across all folds. The sample misprediction plots depict the fraction of times a sample was assigned to the wrong label (ENDO/ non-ENDO) across all models and all folds for each respective dataset (training, validation and evaluation). A grey misprediction fraction indicates that the sample was not included in any of the folds. The columns labelled "fold1" to "fold5", indicate whether the sample was included in the evaluation set ("eval") or the validation ("valid"). The associated numbers (0, 0.2, 0.4, 0.6, 0.8) represent the frequency with which that sample appeared in the validation set across the cross-validation splits. A value of 0 means it was never in the validation set and only used for training, while 0.8 suggests it was in the validation set in 80% of the splits.

Regarding the suggestion to perform more folds: while we understand the theoretical benefit of increasing *k* in cross-validation, our critical stratification variables and finite size of this comparatively large cohort in the field makes this impractical. We do not believe that performing more folds would yield a more stable estimate of model performance, for two main reasons: 1) increasing k in the k-fold setup would result in each evaluation set having fewer samples and therefore being more imbalanced with regard to stratifying variables such as endometriosis grade and sequencing batch, meaning the variance across folds would likely increase - not decrease; and 2) performing additional Monte-Carlo CV splits on our cohort during model tuning would only result in the same samples being chosen across multiple folds. This could artificially reduce apparent variance, not because of greater model stability, but because the same samples are reused across folds.

We hope this answer appropriately addressed the reviewer's concerns regarding AUC variance.

5. Reviewers suggested looking at alternative DEG and integrating DEG methods to account for method bias. This is a well-established issue in the field and likely leads to biased results.

To respond to this comment, we have performed DEG analysis with one of the most frequently used methods, the Seurat function FindMarkers, using the same configuration as for the Muscat DEG analysis in the manuscript comparing patients diagnosed with endometriosis to the control population. A high number of DEGs were found (n = 10 518). This is concordant with the finding that methods operating at the single-cell level yield a high number of DEGs, likely increasing the risk of false positives (https://www.nature.com/articles/s41467-021-25960-2). In contrast, methods such as Muscat, which are specifically tailored to sample-level predictions, produce more reliable results with better type I error control (https://www.nature.com/articles/s41467-021-25960-2#Sec10).

Nonetheless, a good overlap between the two methods was observed, with 40% of the cell type DEGs identified through Muscat shared with the FindMarkers analysis (n=126). All 11 genes shared by ScaiVision models and the DEG analysis from Muscat were also found differentially expressed when using FindMarkers. Genes uniquely identified by Muscat have generally a lower fold change (p_val $< 2.2e-16$ using Wilcoxon rank-sum test, greater alternative). FindMarkers filters out by default genes with a fold change below 0.1, which may explain why these genes were not detected by FindMarkers.

6. Can you extend beyond cell types to identify disease-associated cell states, not just cell types?

Our approach extends beyond cell type identification by leveraging ScaiVision's ability to rank cells based on disease predictiveness, helping to identify disease-associated cell states. For example, we found that a high percentage of mural cells, dendritic cells, and various epithelial cell types were found among the top 10% of predictive cells (Fig. 4e, Supplementary Table 7). These findings align with altered gene expression and ligand-receptor interactions seen in disease (Fig. 3), as well as increased VEGF signaling in mural cells (Fig. 3g).

In response to the reviewer's question, we performed an additional analysis comparing the top 10% of endometriosis-associated predictive cells to the rest of the cells within these cell-type. We found angiogenesis, regulation of cell adhesion, endothelial cell migration and response to cytokine to be enriched in mural cells, inflammatory response, lysosome and regulation of programmed cell death was upregulated in macrophages and cilium assembly, organization and movement was upregulated in ciliated cells (full table added for now in endometriosis_endometrium_scRNA_atlas\Tables\GO_enrichment_predictive_cells_E066A002.csv). These findings are in line with the results described in the manuscript.

Moreover, when examining the UMAP and applying various clustering methods, we did not observe any distinct clusters segregated strictly by disease status (ENDO vs. non-ENDO) when excluding sample and menstrual cycle phase-driven effects. If not captured in our analysis, we would appreciate further clarification from the reviewer on the specific type of disease-associated cell states they are referring to.

7. All reviewers suggested validation of some biological results. Given the well-established biases of the analysis methods, this remains something that would be incredibly valuable.

In response to the reviewers' suggested validation of biological results, we confirmed the differential expression of CXCL3 with immunohistochemistry (Fig. 4g, Extended Data Fig. 8).

8. Reviewer #3 asked about significance thresholds and if multiple testing correction has been performed. The rebuttal pointed to the reply to Reviewer #1 comment 11d. This comment relates to the practicality of a gene signature from proliferative stage endometrium for a diagnostic test. It's not clear what has been changed, or indeed if multiple testing correction was performed.

We apologize for the oversight in our previous response. We intended to refer to Reviewer #1 comment 12d, not 11d. Here in detail:

Thank you for pointing out this important subject. We added this information everywhere applicable in the manuscript (Fig. 1e, Fig. 2e and f, Fig. 3a, b, Fig. 4d, Extended Data Fig. 1e and f, Extended Data Fig. 4b and Extended Data Fig. 7e). For the DEG analysis with muscat, we used a cut-off value of 0.05 of the local adjusted p.value given by muscat.
Comment to Fig. 3e-h: In CellChat (DOI: 10.1038/s41467-021-21246-9), significant interactions between two cell groups through a ligand-receptor pair are identified using a permutation test by randomly permuting the group labels of cells 100 times. The author does not recommend further multiple testing. We show only interactions that were considered significant in both ENDO and non-ENDO, with the main focus on interaction strength change between ENDO and non-ENDO.
We removed the word 'significant' for the overall enriched pathways found in Figure 3c.

Reviewer #3 (Remarks on code availability):

Code is available for some areas of analysis. Data paths are hard coded and not not commented, so reproducing analysis would be challenging

We greatly appreciate the reviewers' constructive comments regarding code availability and reproducibility. In response, we have made significant improvements to the GitHub repository. Specifically, we have reorganized the structure to enhance clarity and ease of use. Detailed descriptions have been added to the repository, as well as the code to reproduce the figures and supplementary data figures. Furthermore, we have included the necessary data paths, provided links for downloading the relevant data, and added clear instructions on how to reproduce the results presented in the paper. Additionally, we have populated the author's GitHub profile to facilitate better access to the relevant resources. We believe these updates will significantly improve the transparency and reproducibility of the analysis.

Reviewer #4 (Remarks to the Author):

The author's response has made a commendable effort to articulate the uniqueness of their study and to compare it in detail with previous research. However, there are still some issues:

1.Previous review comments mentioned that the pathophysiological mechanisms discussed in the study are not novel. While the author's response lists their unique dataset and methods, it does not sufficiently emphasize how these methods bring new insights into the pathophysiological mechanisms. Moreover, the previously mentioned review comments noted that the biological pathways and molecular mechanisms have been extensively reported in studies on endometriosis. The authors' response should specifically point out what new regulatory mechanisms or modes of action their study has discovered in these known pathways, particularly highlighting how their single-cell data reveal the cell type specificity of these processes.

While we acknowledge the reviewer's point, we would like to clarify that some of the comments raised by Reviewer 2 regarding the novelty of our findings were incorrect, as detailed in our responses to Reviewer 2. Our study contributes novel insights into the pathophysiology of endometriosis by identifying cell-type-specific regulatory mechanisms through comprehensive single-cell analysis of the endometrium. While we confirm the involvement of some known biological pathways such as TNF and VEGFA, our research provides unprecedented detail on the cell-type-specific interactions and regulatory mechanisms. The use of our large single-cell atlas, precise cell annotations, and menstrual cycle phasing enables us to explore these interactions in ways not previously reported.

Key findings include:

Inflammation and Adhesion:

Our study reveals a macrophage mediated enhanced inflammation in the endometrium of women with endometriosis through a significant increase in subtypes MΦ4 and MΦ5 interactions with structural endometrial cell via TNF–TNFR1/2 signaling. This signaling pathway is known to activate the NFκB and MAPK pathways, which we observed to be elevated. We also demonstrate heightened macrophage-dendritic cell-epithelial cell interactions with immune cells through ICAM1/2- (ITGA*+ITGB2)/SPN, potentially increasing the recruitment of circulating leukocytes and contributing to an inflammatory microenvironment that favors lesion formation. Furthermore, we uncover an enhanced interaction between macrophage subtypes and epithelial cell subtypes with endothelial cell subtypes through the chemokines CXCL2, CXCL3, CXCL8 with ACKR1, known to regulate inflammatory response.

Proliferation and Cell Survival:

We identify a novel reduced signaling interaction between the cell death receptor TMEM219 and IGFBP3 in eutopic mesenchymal cells in endometriosis. Coupled with upregulated NFκB and ALK signaling, this reduced signaling may contribute to enhanced cell survival and proliferation, aiding the establishment and progression of endometriotic lesions.

Angiogenesis:

Our study is the first to detail the cell-type and receptor-specific interactions in VEGF signaling, highlighting the significant roles for macrophages and endothelial cells through VEGFA-VEGFR1/-VEGFR2, as well as mural cells and endothelial cells through PGF-VEGFR1 interactions. We show a significant increase in secretory mural cell abundance in the endometrium of women with endometriosis aligning with endothelial cell proliferation via enhanced VEGF signaling in ENDO. These findings in the endometrium suggest an early link to stem cell behavior raising the potential perivascular endometrial mesenchymal stem cell transformation may already be occurring in the endometrium, providing critical and novel support for the 'stem cell hypothesis' of endometriosis pathogenesis.

We also observe an increase in TGFβ signaling and glycolysis/gluconeogenesis pathways, suggesting a Warburg-like effect in the eutopic endometrium, which could further promote angiogenesis. While TGFβ signaling in endometriosis has been reported previously, our work provides additional evidence for its role in these processes in the endometrium.

Nervous System and WNT/NOTCH Pathways:

Our analysis additionally reveals a microenvironment in the endometrium of women with endometriosis conductive of nerve fiber infiltration, driven by axon guidance-related genes. Increased expression of these genes supports the presence of nerve fibers in the endometrium (Al-Jefout et al., 2009, doi:10.1093/humrep/dep275). If refluxed into the peritoneal cavity, this provides a mechanism for the lesions to interact with existing nerve fibers, potentially facilitating a feedback loop that contributes to painful sensations. Furthermore, we observe subtle increases in WNT signaling (WNT2/3/7A - (FZD3/4/6+LRP6) and NOTCH signaling, which may influence epithelial differentiation in the endometrium, a feature previously observed in endometriosis lesions.

In summary, our single-cell analysis uncovers new regulatory mechanisms in known pathways as well as new interactions, revealing how macrophages, immune cells, endothelial cells, and epithelial cells interact in a highly specific and refined manner.

These findings provide a deeper understanding of the cellular and molecular processes driving endometriosis and highlight potential therapeutic targets for future research.

2.Your response briefly mentions the SDF-1α/Rho/mDia pathway influenced by CXCL12. However, it lacks a detailed discussion on how this pathway specifically contributes to the pathological nerve growth observed in endometriosis. If possible, please include new data or specific results from the study that show the differential expression of CXCL12 and its impact on nerve growth. If the study includes pathway analysis, highlighting how these pathways are enriched in endometriosis samples compared to controls would be valuable.

Thank you for your detailed feedback. The mention of the SDF-1α/Rho/mDia pathway influenced by CXCL12 was intended as a brief reference to its potential mode of action, given its established role in axon pathfinding. While our study includes pathway enrichment analysis that highlights axon guidance and nervous system development as significant terms in ENDO mesenchymal cells, the current findings do not definitively demonstrate a causal link between CXCL12 and nerve growth and we chose not to overextend our interpretation.

3.The reviewer suggested the use of spatial proteomics or spatial transcriptomics techniques to validate the interactions between cells. The author mentioned using spatial immunohistochemistry to validate the expression of CXCL3. Although this is a good start, the author did not specify how this method compares with other spatial techniques recommended by the reviewer, nor whether other spatial data analysis techniques were considered or planned for use in the study. Additionally, the author mentioned cross-validation methods in their response, including differential gene expression (DEG) analysis, ligand-receptor analysis, and AI analysis. However, they did not provide detailed descriptions of how these methods were specifically applied to their dataset, nor how these analyses support their conclusions interactively.

Quantitative validation of ligand-receptor interactions with spatial methods indeed presents significant challenges. As mentioned, we validated the expression of CXCL3 in the endometrium of women with endometriosis using spatial immunohistochemistry. At this stage, we do not plan to perform additional spatial techniques beyond immunohistochemistry.

To support our findings comprehensively, we implemented cross-validation methods, including differential gene expression (DEG) analysis, ligand-receptor interaction analysis, and annotation-independent AI gene signature analysis. These methods complement each other to reinforce our conclusions. For instance, key biological processes such as chemokine signaling and angiogenesis/VEGF pathways were consistently identified through DEG analysis, ligand-receptor predictions, and AI-based

gene signature analysis. This convergence of evidence strengthens the validity of our findings.

The interactive conclusions from these analyses are summarized in Fig. 5, which integrates the insights from the various approaches applied in this study. While we recognize the broader utility of spatial data analysis techniques, we believe that our current approach, combining computational cross-validation methods with the targeted spatial validation, sufficiently supports the main conclusions of our work.

4.The author mentions specificity and sensitivity achieved with bulk sequencing data but does not detail the specific validation techniques used for the machine learning model developed from single-cell data, which is the focus of the paper. Moreover, the author does not discuss how the model performs under different conditions or with different datasets. Information on how the model handles variability in data, which is crucial for demonstrating robustness and generalizability, is lacking. In addition, the reviewer suggested using external validation with independent datasets as a way to strengthen the reliability and applicability of the predictive model. The author's response does not address whether such external validation has been or will be conducted.

The main focus of the paper is the pathophysiology of endometriosis. Suitable comparable single-whole-cell datasets for endometrial tissue are currently not available in a high enough number for a thorough external validation. We could, however, successfully translate the signature to bulk RNA-seq by training standard machine learning models using our single-cell signatures as well as distilling our best-performing single-cell signature through iterative gene selection into a core gene set suitable for a more cost-effective qPCR approach and large-scale clinical validation study. The specificity and sensitivity achieved with bulk sequencing data were only mentioned in a review response (Reviewer #2, point 4) and the editor did originally not favor its inclusion (we could however include these results if deemed useful). To clarify this point in the manuscript, we have expanded the discussion to include how the external validation would be conducted.

Reviewer #4 (Remarks on code availability):

After examining the code, I realised that reproducing the results in the paper was impossible. The github links lacked an introduction to the data as well as a download link, and there was no introduction to the code, such as what each code was used for, or to reproduce which figure from the paper. The author's github profile is blank, which is not conducive to reproducing the results of the paper.

We greatly appreciate the reviewers' constructive comments regarding code availability and reproducibility. In response, we have made significant improvements to the GitHub

repository. Specifically, we have reorganized the structure to enhance clarity and ease of use. Detailed descriptions have been added to the repository, as well as the code to reproduce the figures and supplementary data figures. Furthermore, we have included the necessary data paths, provided links for downloading the relevant data, and added clear instructions on how to reproduce the results presented in the paper. Additionally, we have populated the author's GitHub profile to facilitate better access to the relevant resources. We believe these updates will significantly improve the transparency and reproducibility of the analysis.

REVIEWER COMMENTS

Reviewer #3 (Remarks on code availability):

Code was avaliable but I was unable to run it due to hardcoding of file paths.

Hardcoded file paths have been removed from the code. Please let us know if you find any remaining instances. As detailed in the README, data from GSE111976 must be downloaded and linked to the _Data folder of the code repository before running the code.

Reviewer #4 (Remarks to the Author):

In their response, the authors highlight the study's new insights into the pathophysiology of endometriosis through cell type-specific single-cell analysis and describe improvements in code and GitHub data availability. However, the authors seem intent on not addressing these issues and there are a number of questions that must be clarified or added to the evidence.

Q1:The authors list new cell-type-specific interactions within known pathways (e.g., TNF, VEGF, TGFβ), yet the response lacks a direct, side-by-side comparison showing exactly what is novel relative to published work.

####Response to Q1 and Supplementary Table 10 need to be double-checked!!!

We thank the reviewer for this suggestion. In the revised manuscript, we have clarified the novelty of our findings in three ways:

1. **Comparison of single-cell studies:** We added a manuscript-ready table (Supplementary Table 10) that summarizes existing single-cell studies of the eutopic endometrium, including sample sizes, menstrual cycle stratification, hormonal treatment status, and major limitations. This highlights how our atlas advances beyond prior work by including the largest hormone-naïve, premenopausal cohort to date, analyzed with rigorous menstrual cycle stratification and uniform processing.

2. **Comparison of pathways and interactions:** We expanded the discussion with a structured side-by-side comparison (Supplementary Table 10) of major pathways previously reported (e.g., TNF, VEGF, TGFβ, WNT/NOTCH) versus the novel, cell-type-specific interactions we identified. For example, while VEGF signaling has been broadly implicated in endometriosis, our study provides the first receptor- and cell-type-resolved map at single-cell resolution, uncovering macrophage–endothelial and mural–endothelial VEGF axes and linking mural

cell expansion in ENDO to the “stem cell hypothesis.” Similarly, we detail previously undescribed adhesion and survival mechanisms (e.g., ICAM1/2–ITGA*+ITGB2/SPN, TMEM219–IGFBP3).

3. **Integration into the discussion:** We adapted the discussion to emphasize that the novelty of our findings is not only in the identification of new interactions, but also in the ability to disentangle disease-related changes from physiological menstrual cycle variation. Some prior studies reported changes in similar pathways; however, these were most likely menstrual cycle-driven rather than disease-specific due to the lack of phasing. By combining a large donor cohort with careful cycle phasing, our atlas distinguishes true endometriosis-specific signatures from normal menstrual cyclical fluctuations in angiogenesis, adhesion, immune function, WNT/NOTCH, and TGFβ signaling. This distinction was not possible in earlier work but is central to interpreting endometrial biology correctly.

Together, these additions provide a clear, side-by-side comparison to the literature and highlight both the methodological advance of menstrual cycle-resolved single-cell analysis and the novel biological insights it enables.

As note, Reviewer #1 positively commented on the novelty:

“This study finds an area previously not pursued in detail in earlier single cell atlases on endometrium and/or endometriosis (refs 16, 17, 23, 58), therefore adds to important and new information in a generally neglected tissue.”

Q2. The brief mention of the CXCL12-driven SDF-1α/Rho/mDia pathway and its role in aberrant nerve growth lacks direct data support (e.g., differential expression, pathway enrichment). Please provide pathway-level analyses (e.g., GSEA) or gene-expression data demonstrating significant up-regulation in endometriosis samples. Moreover, if this pathway is important, it is still recommended to illustrate the significance of CXCL12 or SDF-1α/Rho/mDia in ENDO patient tissues at least in Supplementary with differential expression results or pathway enrichment maps.

Thank you for this comment. One would expect the SDF-1α/Rho/mDia pathway to be upregulated in nerve fibers, which are not represented in our single-cell dataset due to methodological limitations, as nerve fibers are not captured with standard single-cell dissociation protocols. Therefore, the brief speculative mention of the CXCL12-driven SDF-1α/Rho/mDia pathway cannot be directly supported by pathway-level evidence in our data and we have removed the mention.

Q3. If it is difficult to carry out further spatial genomics experiments, it is recommended that the methodology be described at least in more detailed length. How was the ligand-receptor analysis done, For example with CellChat/CellPhoneDB/other tools? In which datasets were these analyses implemented respectively? How do the results corroborate with DEG analyses?
The ligand-receptor analysis was performed with CellChat, as noted in the figure legends, Methods section and the main text, with the original CellChat methods publication referenced for full details on the methodology.
Importantly, the full code to the CellChat analysis and figure generation is openly available in our GitHub repository under:

- endometriosis_endometrium_scRNA_atlas/07_Ligand_receptor_analysis
- endometriosis_endometrium_scRNA_atlas/Figures.

In response to the reviewer's comment, we have now expanded the Methods section (see *Ligand-receptor interaction analysis*) to describe the procedure in more detail.

We appreciate the reviewer's question regarding the overlap. There is a good overlap between the ligand/receptor genes (Figure 3e-h and Sup. Figure 5d) and the DEGs (Table 4), with 14 of the 20 shown ligand/receptor genes also being DEGs. To highlight this concordance, we have added new columns to Table 4 titled 'Fig3_Sup5_LigandReceptor,' for easier cross-referencing and emphasizing the consistency across analysis.

Reviewer #4 (Remarks on code availability):

It is a nice code. We can install and run the code.
Thank you for acknowledging the improvement.

Additional points requested by editor and confidentially by Reviewer #3 , question 4: The sample variance of AUC across folds is high. Performing more folds will help accurately estimate this variance.

In order to address the question of inter-fold variance, we have repeated the nested CV-split experiment (training, validation and evaluation) from Figure 4, increasing the number of both inner and outer fold from 5 to 10, and focusing on the pca and corrgpca dimension reduction methods. As before, 50 models were trained per inner CV split and dimension reduction method, leading to a total of 10,000 models being trained (10 outer splits * 10 inner splits * 2 dimension reductions * 50 models). Given the number of samples (n = 28),

we assessed that a true k-fold split with k=10 was not feasible, as each evaluation fold would only contain 2-3 samples, which would not be sufficient to robustly assess model performance. Moreover, while in the manuscript we attempted to balance the evaluation folds with regard to endometriosis grade (severe or mild), this was not possible with 10 folds, as each grade comprises less than 10 samples (7 mild and 8 severe). Therefore, we applied a Monte-Carlo cross-validation scheme, randomly choosing 6 samples with replacement 10 times to be held out for evaluation, while stratifying on disease status (ENDO vs CTL). The resulting distribution of samples across folds can be seen in **Figure 1**. We note that while 5 samples were never selected for evaluation using this procedure, this number is well within the expected outcome for random sampling.

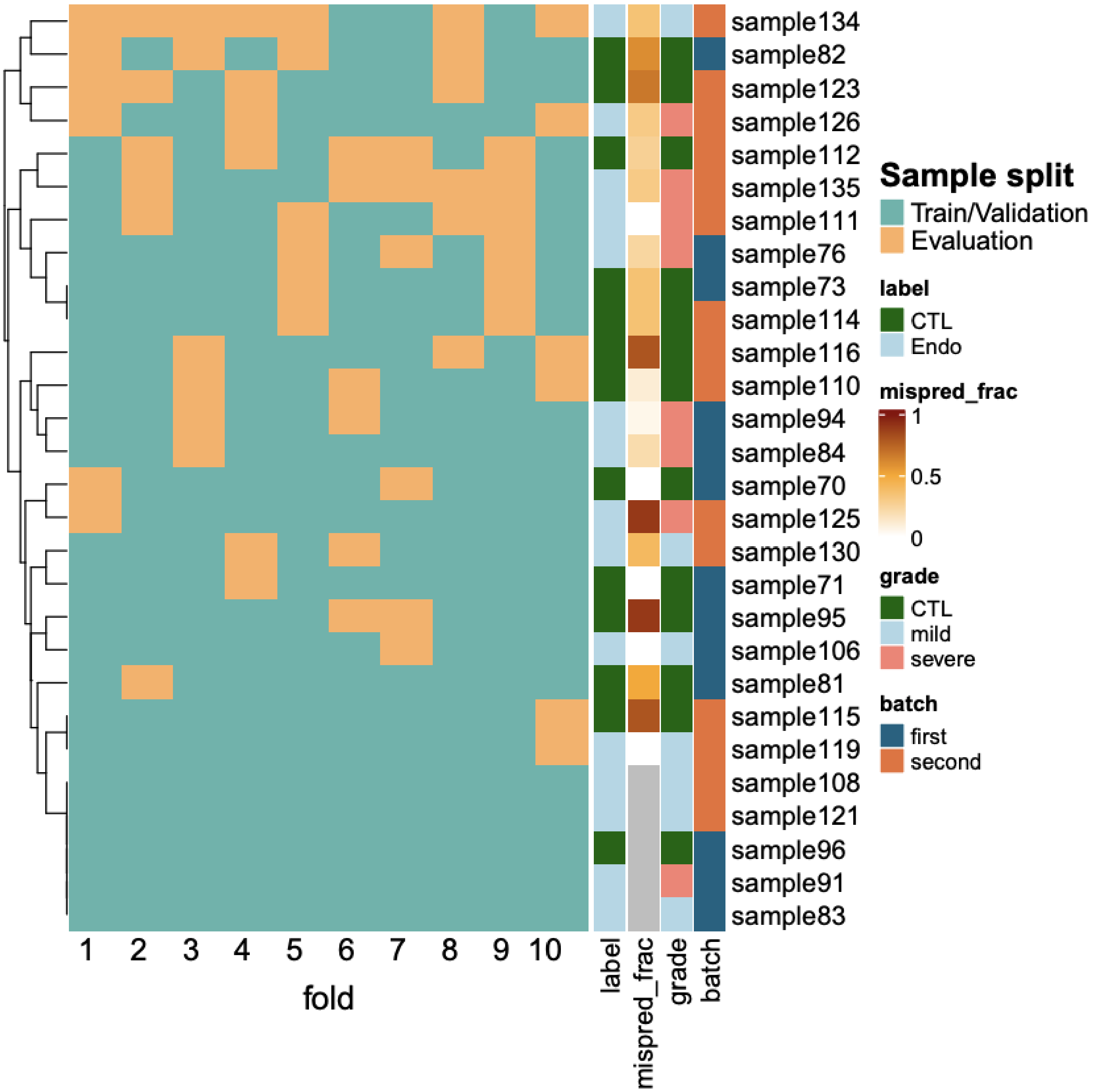

Figure 1. Distribution of samples by evaluation or training/validation split across folds. Annotation columns show disease status (label column, Endo vs. CTL), the fraction of models in which each sample was mispredicted across all evaluation folds, endometriosis grade, and sequencing batch.

The results of model training and validation can be seen in **Figures 2 and 3,** which show the model-wise AUCs grouped by dimension reduction method and outer CV fold. The thick red line in each boxplot indicates the median AUC per outer fold. For each inner fold, the model with the best performance on the validation data (measured by loss) was evaluated on the evaluation data from the corresponding outer fold; hence for each outer fold, 10 models were evaluated. The results on the evaluation folds can be seen in **Figure 4**. In order to quantitatively compare the inter- and intra-fold variance between this experiment and the original experiment from the manuscript, we calculated the standard deviation of the validation and evaluation AUCs within each outer fold, the standard deviation of the median AUC values across folds, and the standard deviation of the standard deviations across folds. The results are presented in **Tables 1-2**.

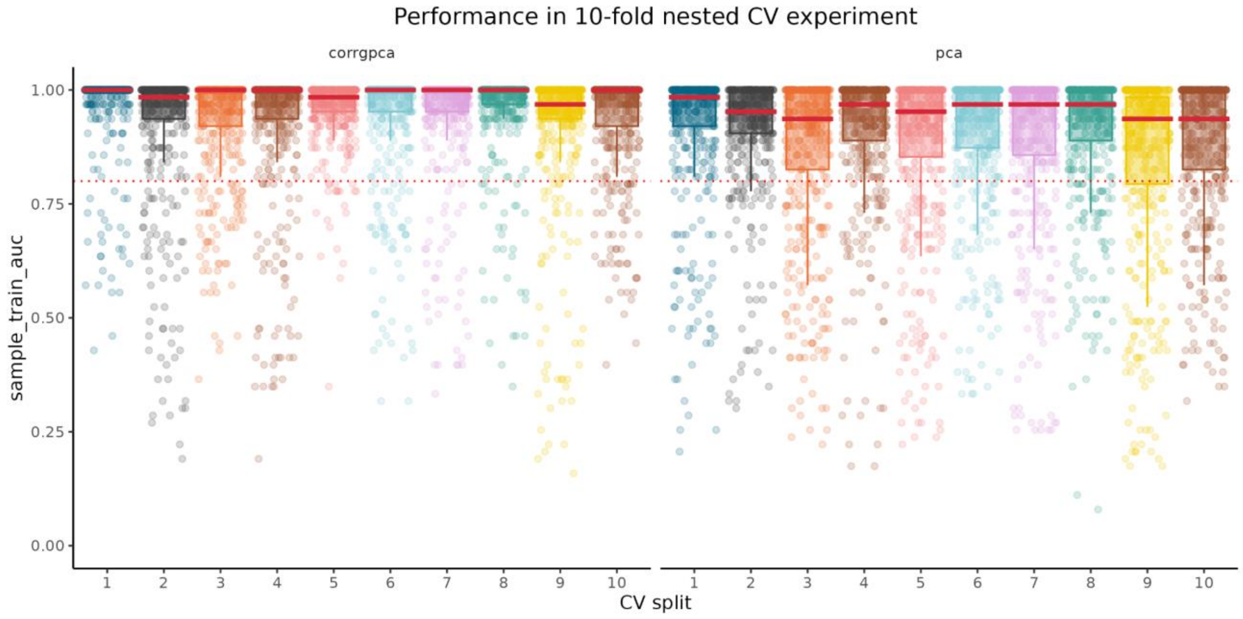

Figure 2. Model-wise AUCs on training data, grouped by outer CV fold and dimension reduction method. Thick red lines indicate the median value across all models within an outer CV fold.

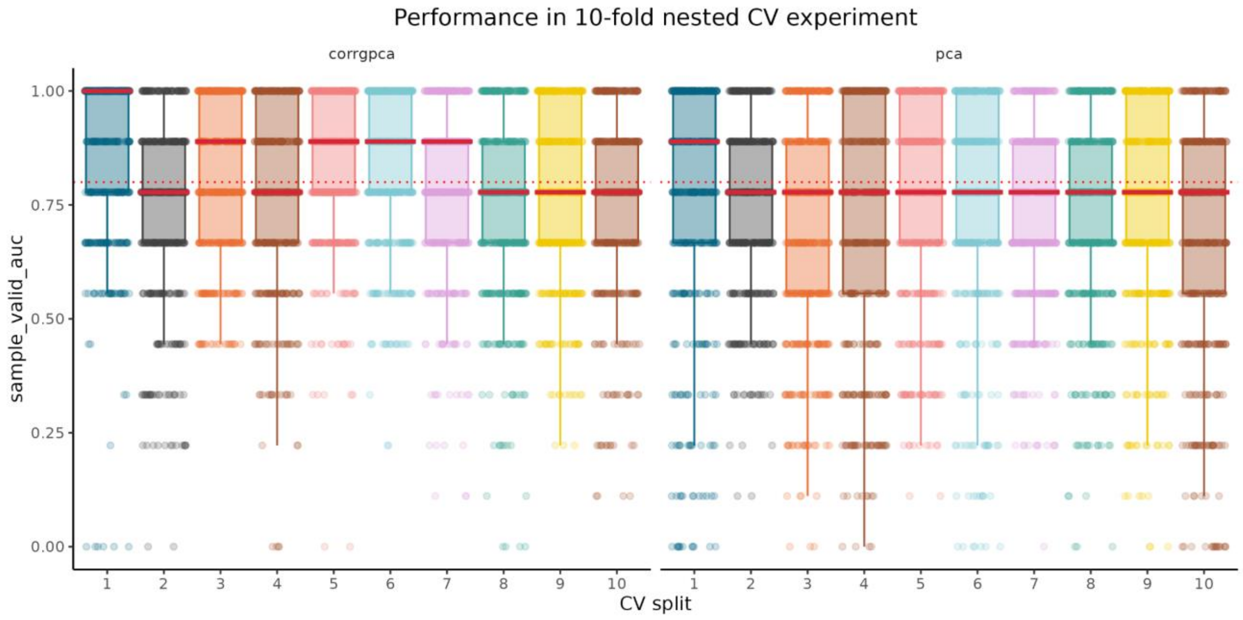

Figure 3. Model-wise AUCs on validation data, grouped by outer CV fold and dimension reduction method. Thick red lines indicate the median value across all models within an outer CV fold.

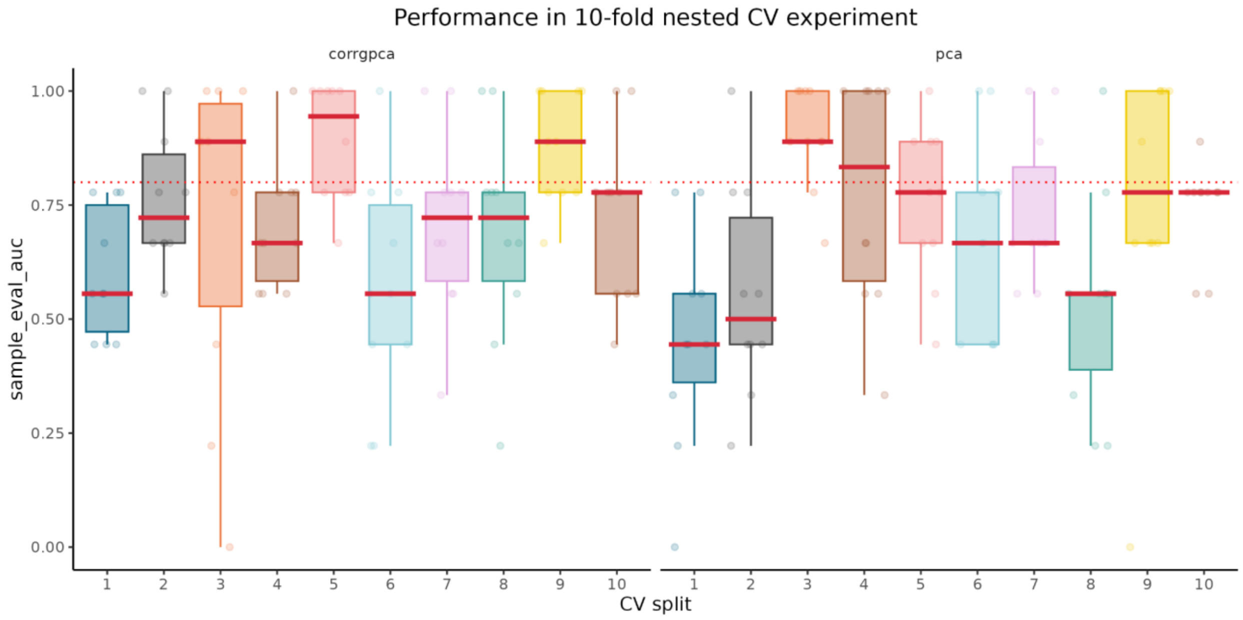

Figure 4. Model-wise AUCs on evaluation data, grouped by outer CV fold and dimension reduction method. Thick red lines indicate the median value across all models within an outer CV fold.

These results show that, as expected, the inter-fold variance does not change significantly when running more folds. On the validation data, the variance between folds is decreased when running 10 folds vs. 5 folds (measured by the standard deviation of the median AUC), from 0.061 to 0.035 for pca and from 0.12 to 0.78 for corrgpca. On the evaluation data, we see a reduction from 0.32 to 0.13 for corrgpca; however, for pca, the values remain similar, at approximately 0.15 in both cases. It is our opinion that the apparent reduction in inter-fold variance for the validation data is deceiving, however. This is because the same number of samples were used for training and validation in each outer fold in both experiments (n = 22), however, in one case they were sampled 5 times to generate the validation data for the inner folds, while in the other case they were sampled 10 times. Hence, necessarily, the validation datasets in the 10-fold CV experiment have a greater overlap in terms of samples than the validation folds in the 5-fold CV experiment, which would be expected to lead to more similar results.

**Dimension reduction method**	**Fold**	**Validation AUC s.d.**	**Validation AUC median**	**Evaluation AUC s.d.**	**Evaluation AUC median**
**Manuscript (5-fold CV):**					
corrgpca	1	0.152	0.778	0.170	0.556
corrgpca	2	0.132	0.667	0.000	1.000
corrgpca	3	0.173	0.778	0.064	0.889
corrgpca	4	0.226	0.778	0.357	0.444
corrgpca	5	0.080	1.000	0.000	0.222
pca	1	0.148	0.778	0.111	0.889
pca	2	0.143	0.667	0.064	0.889
pca	3	0.176	0.778	0.064	1.000
pca	4	0.175	0.667	0.192	0.667
pca	5	0.124	0.778	0.064	0.667
**New experiment (10-fold CV):**					
corrgpca	1	0.181	1.000	0.141	0.556
corrgpca	2	0.202	0.778	0.152	0.722
corrgpca	3	0.170	0.889	0.360	0.889
corrgpca	4	0.199	0.778	0.139	0.667

corrgpca	5	0.162	0.889	0.128	0.944
corrgpca	6	0.168	0.889	0.248	0.556
corrgpca	7	0.180	0.889	0.204	0.722
corrgpca	8	0.199	0.778	0.239	0.722
corrgpca	9	0.184	0.778	0.122	0.889
corrgpca	10	0.198	0.778	0.191	0.778
pca	1	0.258	0.889	0.237	0.444
pca	2	0.198	0.778	0.234	0.500
pca	3	0.237	0.778	0.110	0.889
pca	4	0.268	0.778	0.251	0.833
pca	5	0.220	0.778	0.172	0.778
pca	6	0.231	0.778	0.222	0.667
pca	7	0.191	0.778	0.167	0.667
pca	8	0.218	0.778	0.239	0.556
pca	9	0.222	0.778	0.309	0.778
pca	10	0.259	0.778	0.105	0.778

Table 1. Summary of within-fold variance (s.d.) and median AUCs for both validation and evaluation datasets, for original 5-fold CV experiment and new 10-fold CV experiment.

**Dimension reduction method**	**Interfold validation AUC s.d. s.d.**	**Interfold validation AUC s.d. median**	**Interfold evaluation AUC s.d. s.d.**	**Interfold evaluation AUC s.d. median**
**Manuscript (5-fold CV):**				
corrgpca	0.054	0.122	0.151	0.320
pca	0.022	0.061	0.056	0.149
**New experiment (10-fold CV):**				
corrgpca	0.015	0.078	0.074	0.134
pca	0.026	0.035	0.065	0.149

Table 2. Summary of inter-fold variance, calculated as both the s.d. of the per-fold AUC s.d. and the s.d. of the per-fold median AUC, for both validation and evaluation datasets, for original 5-fold CV experiment and new 10-fold CV experiment.

To examine how hyperparameter selection contributes to intra-fold variance, we analysed all models from the new 10-fold CV experiment by grouping them according to their unique hyperparameter combinations (n_filters, dropout_prob, weight_decay, learning_rate, l1_filter_regularisation, l2_output_regularisation, percentage_pool) and calculating the median validation loss and inter-quartile range (IQR) per combination. This confirmed that hyperparameter choice affects performance; however, the IQRs remain substantial, indicating that hyperparameters alone do not explain the full variance. The top 40 combinations, each comprising 200 models, ranked by median validation loss for each dimension reduction method are shown in the bar plots in **Figure 5.**

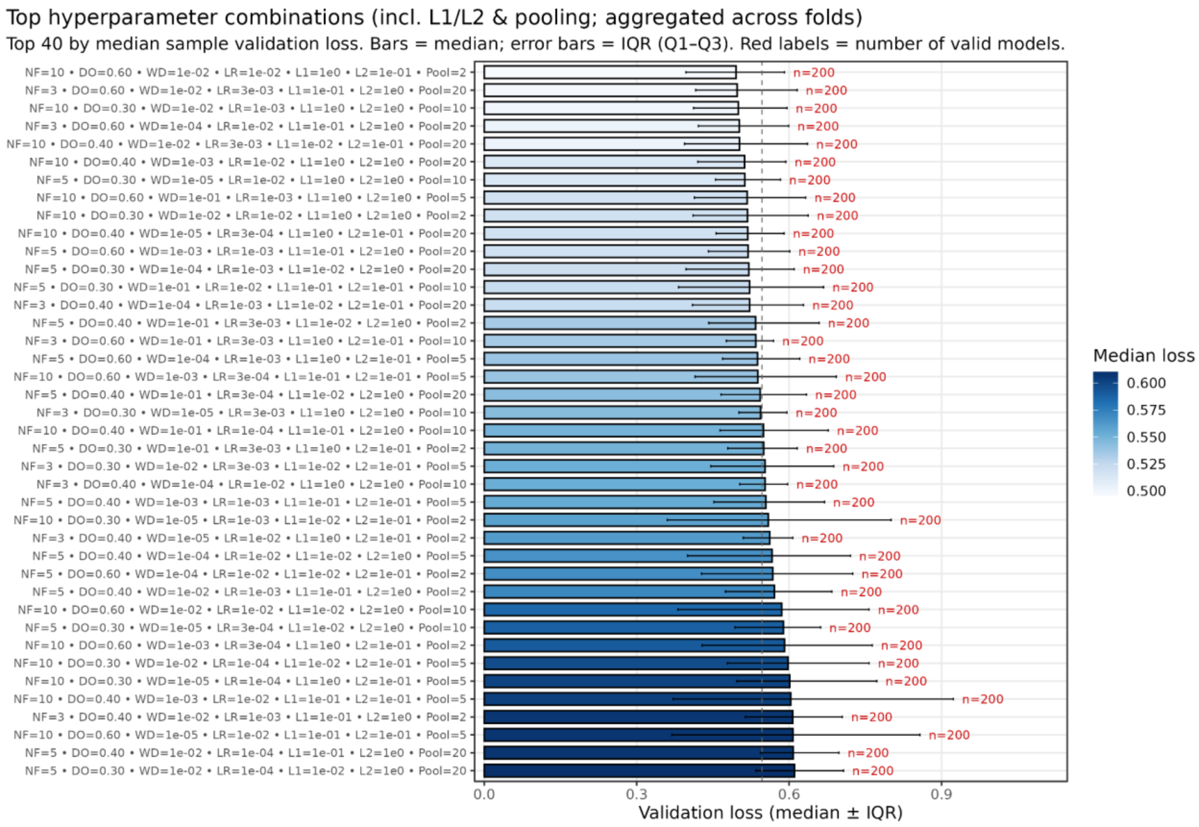

Figure 5. Barplots showing the median validation loss for models from the 10-fold CV experiment, grouped by hyperparameter combination and ranked by increasing loss. Error bars show the interquartile range for each combination.

Next, we fixed the hyperparameters to the best-performing combination identified in the search (n_filters = 10, dropout_prob = 0.6, weight_decay = 1e-02, learning_rate = 1e-02, l1_filter_regularisation = 1, l2_output_regularisation = 1e-01, percentage_pool = 2) and retrained 50 models using the training and validation datasets from outer CV Fold 1 and inner CV Fold 1 from the full experiment. We then compared the variability of models trained with the same data but variable hyperparameters in the full experiment with the variability under fixed hyperparameters by calculating the standard deviation of the validation AUC and loss. The results are shown in **Table 3**.

**CorrGPCA (Fold 1), n = 50:**	Variable hyperparameters	Fixed hyperparameters
Validation AUC s.d.	0.181	0.141
Validation loss s.d.	0.203	0.343
**PCA (Fold 1), n = 50:**		
Validation AUC s.d.	0.258	0.153
Validation loss s.d.	0.413	0.205

Table 3. Within-fold variance, calculated as both the s.d. of the validation AUC and the s.d. of the validation loss, for data from outer and inner fold 1, in the setting of variable hyperparameters or fixed hyperparameters across models.

In both dimension-reduction settings, fixing the hyperparameters reduces the AUC variance, indicating that part of the intra-fold variance originates from hyperparameter fluctuations. However, a substantial amount of variance remains even under fixed hyperparameters, particularly for validation loss in the CorrGPCA models. Of note, the CorrGPCA models displayed a similar amount of variability with variable hyperparameters as the PCA models did with fixed hyperparameters, indicating that this dimension reduction method may be less sensitive to the exact hyperparameter choice. This confirms that stochastic factors such as the random ordering of samples during training contribute meaningfully to the observed intra-fold variability. Although using variable hyperparameters across models increases the intra-fold variability in performance, this is by design, as it allows us to sample the hyperparameter space during the model tuning stage and gives a better chance to find hyperparameter settings that yield good performance.

In summary, we have made a good-faith effort to address the concerns of both Reviewer 3 and the editor regarding inter- and intra-fold variability in model performance. We showed that, while the choice of hyperparameters does affect performance, the

magnitude of this effect can vary across dimension reduction methods, and other factors such as the random order in which the data is presented to the model during training can also contribute to intra-fold variability. We also performed the requested 10-fold nested CV experiment, and while this did show reduced inter-fold variability, we again stress the importance of the increased overlap between samples in validation sets across folds, which was mathematically necessary in order to generate twice the number of folds with the same number of samples. We recognize the limitations imposed on our models by the small sample size available. Nonetheless, we believe that the procedure employed in the manuscript of not relying on the output of any one single model, but rather deriving a consensus signature from multiple high-performing models, allows our results to gain robustness even in this limited setting. We sincerely hope that the analyses and results presented herein satisfy the concerns previously raised.

Reviewer #4

Thank you for the positive feedback. Based on the received editorial guidance, we have updated the text of the previous point-by-point response to explicitly describe the additional analysis as a repeated random-split (Monte Carlo) nested CV experiment and to clarify how the sampling was performed. In addition, we have fixed the discrepancy in the reported variance values which you spotted; this was indeed a typo and should have read “0.078” instead of “0.78” in the text.

Updated text:

In order to address the question of inter-fold variance, we have repeated the nested CV-split experiment (training, validation and evaluation) from Figure 4, increasing the number of both inner and outer splits from 5 to 10, and focusing on the pca and corrgpca dimension reduction methods. As before, 50 models were trained per inner random split and dimension reduction method, leading to a total of 10,000 models being trained (10 outer splits * 10 inner splits * 2 dimension reductions * 50 models). Given the number of samples (n = 28), we assessed that a true k-fold split with k=10 was not feasible, as each evaluation fold would only contain 2-3 samples, which would not be sufficient to robustly assess model performance. Moreover, while in the manuscript we attempted to balance the evaluation folds with regard to endometriosis grade (severe or mild), this was not possible with 10 folds, as each grade comprises less than 10 samples (7 mild and 8 severe). Therefore, we applied a repeated random-split Monte-Carlo nested cross-validation scheme, randomly choosing 6 samples to be held out for evaluation while stratifying on disease status (ENDO vs CTL). This random process was repeated 10 times, replacing the previously-chosen evaluation samples between each sampling. The resulting distribution of samples across outer splits can be seen in **Figure 1**. We note that while 5 samples were never selected for evaluation using this procedure, this number is well within the expected outcome for random sampling.

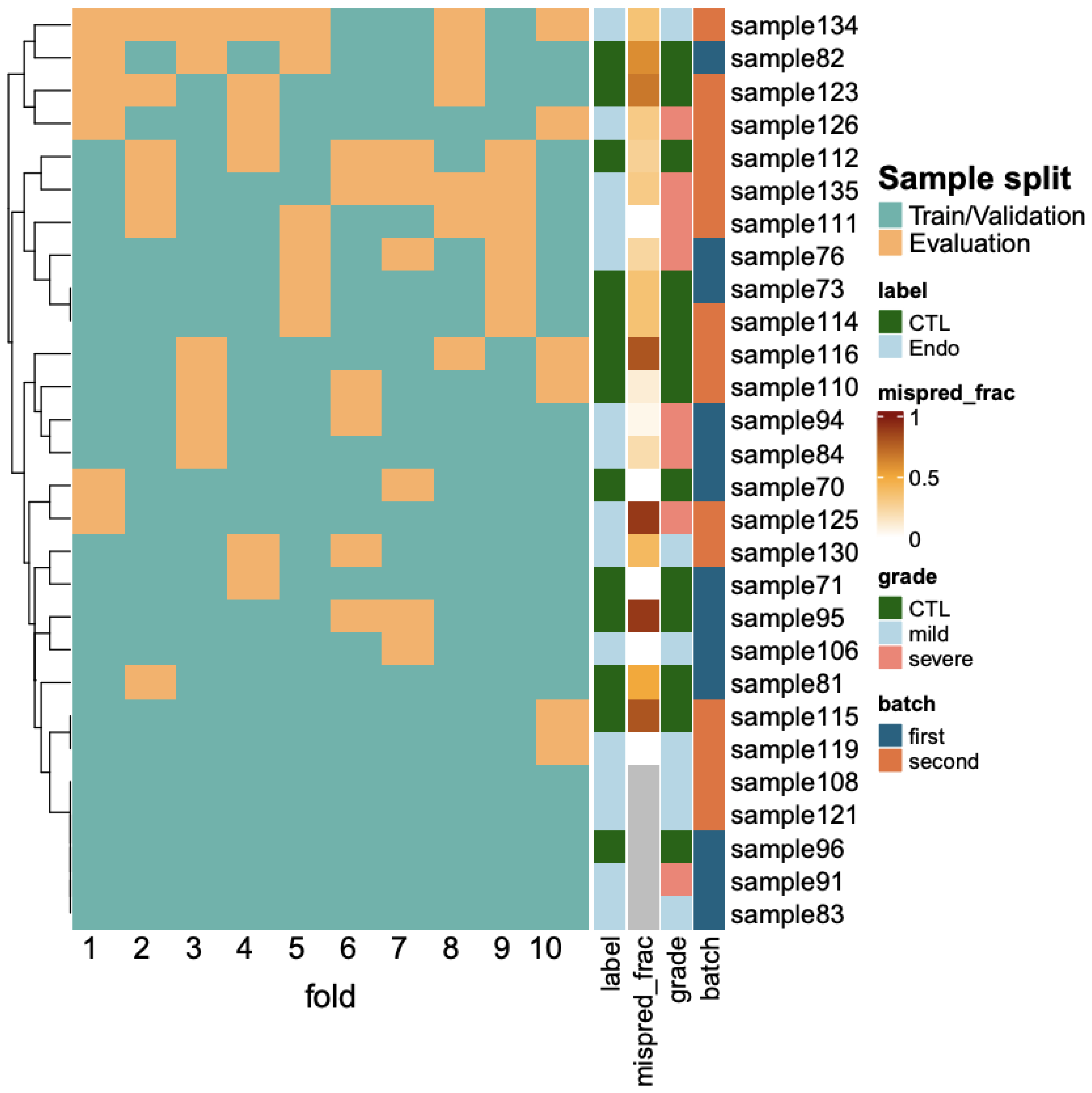

Figure 1. Distribution of samples by evaluation or training/validation split across outer splits. Annotation columns show disease status (label column, Endo vs. CTL), the fraction of models in which each sample was mispredicted across all evaluation splits, endometriosis grade, and sequencing batch.

The results of model training and validation can be seen in **Figures 2 and 3,** which show the model-wise AUCs grouped by dimension reduction method and outer split. The thick red line in each boxplot indicates the median AUC per outer split. For each inner split, the

model with the best performance on the validation data (measured by loss) was evaluated on the evaluation data from the corresponding outer split; hence for each outer split, 10 models were evaluated. The results on the evaluation splits can be seen in **Figure 4**. In order to quantitatively compare the inter- and intra-fold variance between this experiment and the original experiment from the manuscript, we calculated the standard deviation of the validation and evaluation AUCs within each outer split, the standard deviation of the median AUC values across splits, and the standard deviation of the standard deviations across splits. The results are presented in **Tables 1-2**.

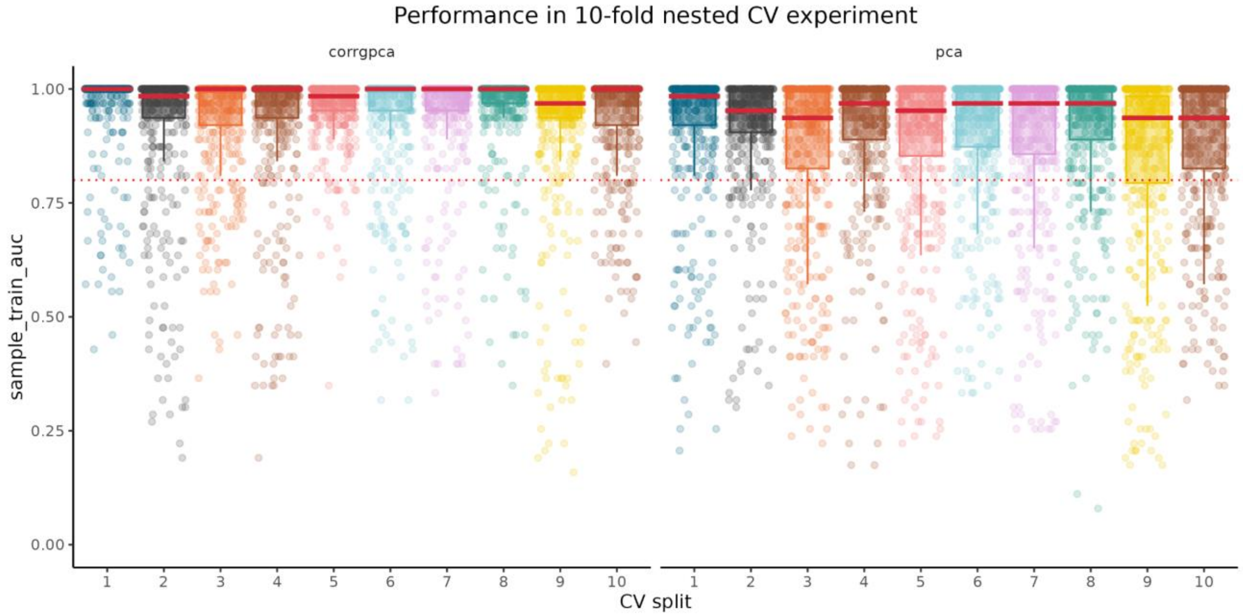

Figure 2. Model-wise AUCs on training data, grouped by outer CV split and dimension reduction method. Thick red lines indicate the median value across all models within an outer CV split.

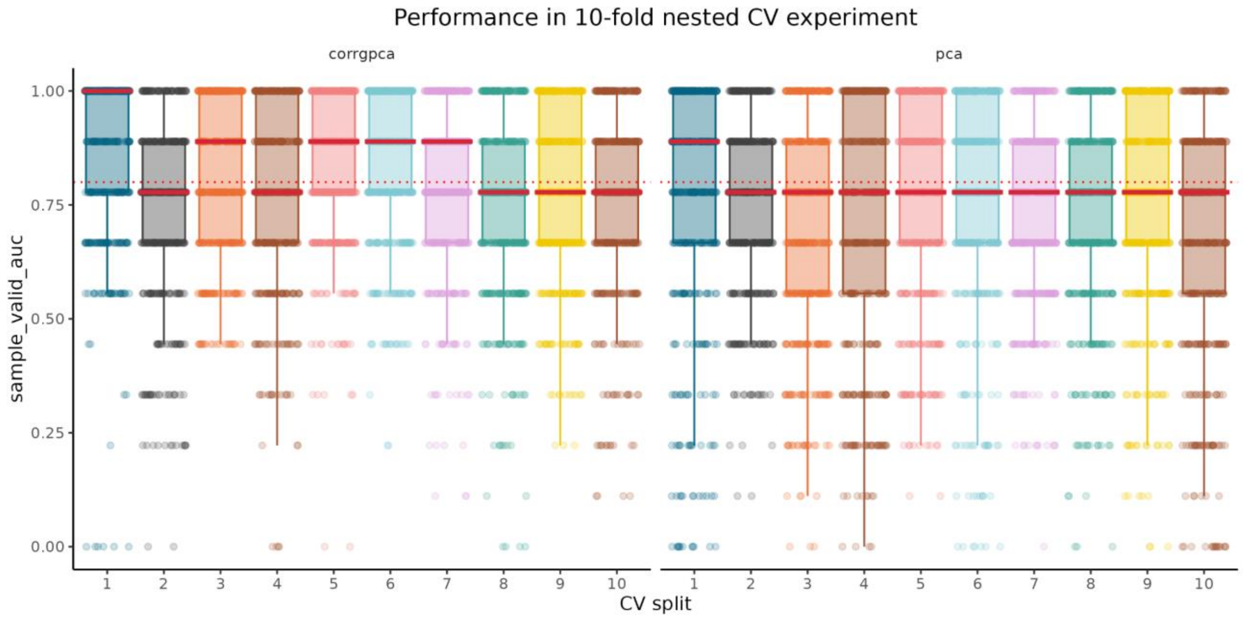

Figure 3. Model-wise AUCs on validation data, grouped by outer CV split and dimension reduction method. Thick red lines indicate the median value across all models within an outer CV split.

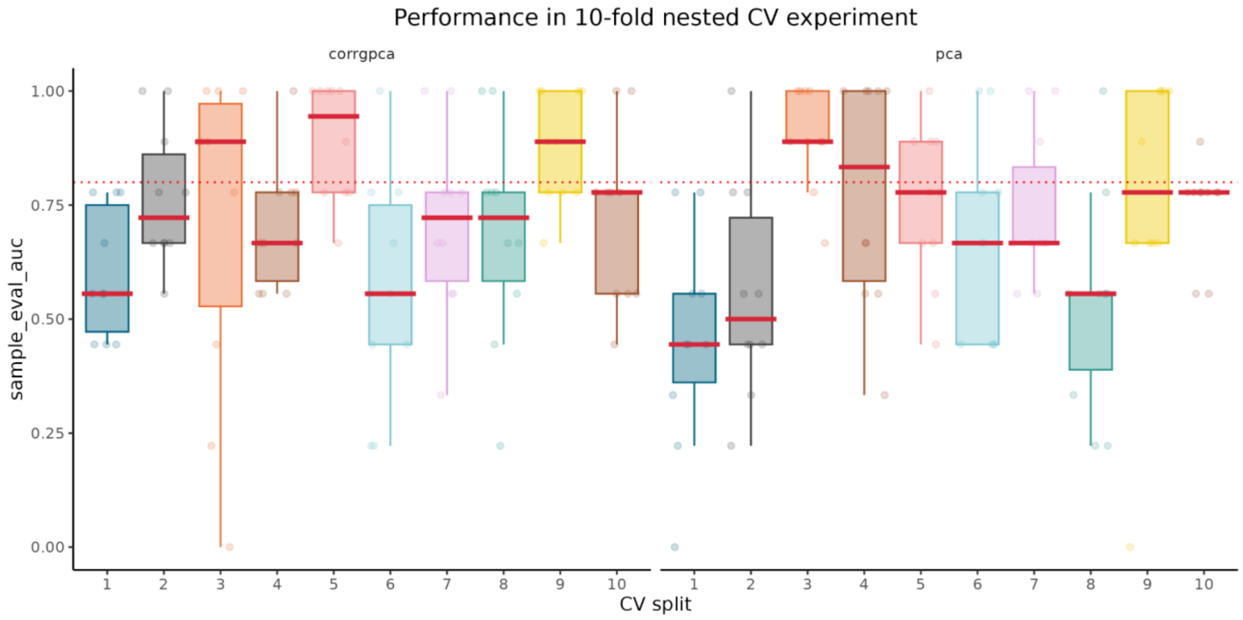

Figure 4. Model-wise AUCs on evaluation data, grouped by outer CV split and dimension reduction method. Thick red lines indicate the median value across all models within an outer CV split.

These results show that, as expected, the inter-fold variance does not change significantly when running more splits. On the validation data, the variance between splits is decreased when running 10 splits vs. 5 splits (measured by the standard deviation of the median AUC), from 0.061 to 0.035 for pca and from 0.12 to 0.078 for corrgpca. On the evaluation data, we see a reduction from 0.32 to 0.13 for corrgpca; however, for pca, the values remain similar, at approximately 0.15 in both cases. It is our opinion that the apparent reduction in inter-fold variance for the validation data is deceiving, however. This is because the same number of samples were used for training and validation in each outer split in both experiments (n = 22), however, in one case they were sampled 5 times to generate the validation data for the inner splits, while in the other case they were sampled 10 times. Hence, necessarily, the validation datasets in the 10-fold nested MCCV experiment have a greater overlap in terms of samples than the validation splits in the 5-fold nested MCCV experiment, which would be expected to lead to more similar results.

Dimension reduction method	Outer split	Validation AUC s.d.	Validation AUC median	Evaluation AUC s.d.	Evaluation AUC median
**Manuscript (5-fold MCCV):**					
corrgpca	1	0.152	0.778	0.170	0.556
corrgpca	2	0.132	0.667	0.000	1.000
corrgpca	3	0.173	0.778	0.064	0.889
corrgpca	4	0.226	0.778	0.357	0.444
corrgpca	5	0.080	1.000	0.000	0.222
pca	1	0.148	0.778	0.111	0.889
pca	2	0.143	0.667	0.064	0.889
pca	3	0.176	0.778	0.064	1.000
pca	4	0.175	0.667	0.192	0.667
pca	5	0.124	0.778	0.064	0.667
**New experiment (10-fold MCCV):**					
corrgpca	1	0.181	1.000	0.141	0.556
corrgpca	2	0.202	0.778	0.152	0.722
corrgpca	3	0.170	0.889	0.360	0.889

corrgpca	4	0.199	0.778	0.139	0.667
corrgpca	5	0.162	0.889	0.128	0.944
corrgpca	6	0.168	0.889	0.248	0.556
corrgpca	7	0.180	0.889	0.204	0.722
corrgpca	8	0.199	0.778	0.239	0.722
corrgpca	9	0.184	0.778	0.122	0.889
corrgpca	10	0.198	0.778	0.191	0.778
pca	1	0.258	0.889	0.237	0.444
pca	2	0.198	0.778	0.234	0.500
pca	3	0.237	0.778	0.110	0.889
pca	4	0.268	0.778	0.251	0.833
pca	5	0.220	0.778	0.172	0.778
pca	6	0.231	0.778	0.222	0.667
pca	7	0.191	0.778	0.167	0.667
pca	8	0.218	0.778	0.239	0.556
pca	9	0.222	0.778	0.309	0.778
pca	10	0.259	0.778	0.105	0.778

Table 1. Summary of within-fold variance (s.d.) and median AUCs for both validation and evaluation datasets, for original 5-fold nested MCCV experiment and new 10-fold nested MCCV experiment.

**Dimension reduction method**	**Interfold validation AUC s.d. s.d.**	**Interfold validation AUC s.d. median**	**Interfold evaluation AUC s.d. s.d.**	**Interfold evaluation AUC s.d. median**
**Manuscript (5-fold nested MCCV):**				
corrgpca	0.054	0.122	0.151	0.320
pca	0.022	0.061	0.056	0.149
**New experiment (10-fold nested MCCV):**				
corrgpca	0.015	0.078	0.074	0.134
pca	0.026	0.035	0.065	0.149

Table 2. Summary of inter-fold variance, calculated as both the s.d. of the per-fold AUC s.d. and the s.d. of the per-fold median AUC, for both validation and evaluation datasets, for original 5-fold nested MCCV experiment and new 10-fold nested MCCV experiment.

To examine how hyperparameter selection contributes to intra-fold variance, we analysed all models from the new 10-fold nested MCCV experiment by grouping them according to their unique hyperparameter combinations (n_filters, dropout_prob, weight_decay, learning_rate, l1_filter_regularisation, l2_output_regularisation, percentage_pool) and calculating the median validation loss and inter-quartile range (IQR) per combination. This confirmed that hyperparameter choice affects performance; however, the IQRs remain substantial, indicating that hyperparameters alone do not explain the full variance. The top 40 combinations, each comprising 200 models, ranked by median validation loss for each dimension reduction method are shown in the bar plots in **Figure 5.**

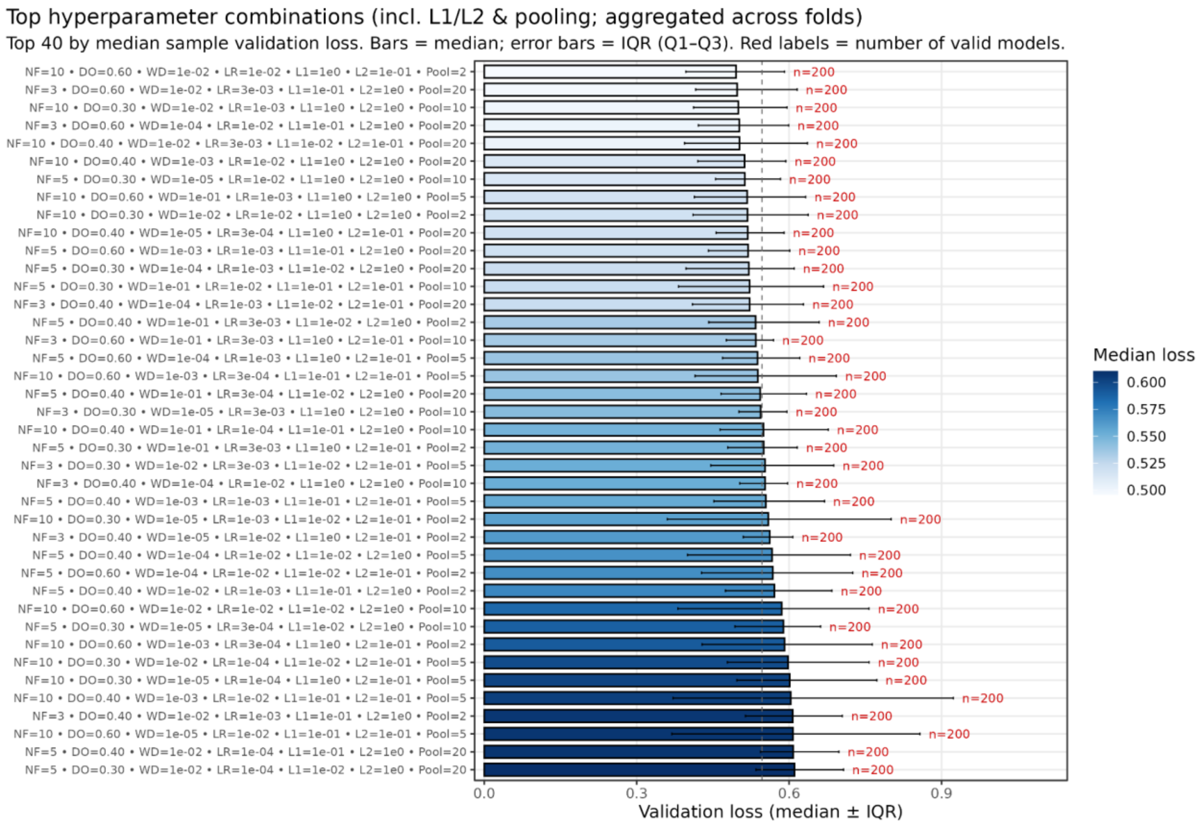

Figure 5. Barplots showing the median validation loss for models from the 10-fold nested MCCV experiment, grouped by hyperparameter combination and ranked by increasing loss. Error bars show the interquartile range for each combination.

Next, we fixed the hyperparameters to the best-performing combination identified in the search (n_filters = 10, dropout_prob = 0.6, weight_decay = 1e-02, learning_rate = 1e-02, l1_filter_regularisation = 1, l2_output_regularisation = 1e-01, percentage_pool = 2) and retrained 50 models using the training and validation datasets from outer Monte-Carlo split 1 and inner Monte-Carlo split 1 from the full experiment. We then compared the variability of models trained with the same data but variable hyperparameters in the full experiment with the variability under fixed hyperparameters by calculating the standard deviation of the validation AUC and loss. The results are shown in **Table 3**.

**CorrGPCA (outer split 1), n = 50:**	Variable hyperparameters	Fixed hyperparameters
Validation AUC s.d.	0.181	0.141
Validation loss s.d.	0.203	0.343
**PCA (outer split 1), n = 50:**		
Validation AUC s.d.	0.258	0.153
Validation loss s.d.	0.413	0.205

Table 3. Within-fold variance, calculated as both the s.d. of the validation AUC and the s.d. of the validation loss, for data from outer and inner Monte-Carlo split 1, in the setting of variable hyperparameters or fixed hyperparameters across models.

In both dimension-reduction settings, fixing the hyperparameters reduces the AUC variance, indicating that part of the intra-fold variance originates from hyperparameter fluctuations. However, a substantial amount of variance remains even under fixed hyperparameters, particularly for validation loss in the CorrGPCA models. Of note, the CorrGPCA models displayed a similar amount of variability with variable hyperparameters as the PCA models did with fixed hyperparameters, indicating that this dimension reduction method may be less sensitive to the exact hyperparameter choice. This confirms that stochastic factors such as the random ordering of samples during training contribute meaningfully to the observed intra-fold variability. Although using variable hyperparameters across models increases the intra-fold variability in performance, this is by design, as it allows us to sample the hyperparameter space during the model tuning

stage and gives a better chance to find hyperparameter settings that yield good performance.

In summary, we have made a good-faith effort to address the concerns of both Reviewer 3 and the editor regarding inter- and intra-fold variability in model performance. We showed that, while the choice of hyperparameters does affect performance, the magnitude of this effect can vary across dimension reduction methods, and other factors such as the random order in which the data is presented to the model during training can also contribute to intra-fold variability. We also performed the requested 10-fold repeated random-split (Monte Carlo) nested CV experiment, and while this did show reduced inter-fold variability, we again stress the importance of the increased overlap between samples in validation sets across folds, which was mathematically necessary in order to generate twice the number of outer splits with the same number of samples. We recognize the limitations imposed on our models by the small sample size available. Nonetheless, we believe that the procedure employed in the manuscript of not relying on the output of any one single model, but rather deriving a consensus signature from multiple high-performing models, allows our results to gain robustness even in this limited setting. We sincerely hope that the analyses and results presented herein satisfy the concerns previously raised.
